# Synthesis of benzannelated sultams by intramolecular Pd-catalyzed arylation of tertiary sulfonamides

**DOI:** 10.3762/bjoc.13.187

**Published:** 2017-09-12

**Authors:** Valentin A Rassadin, Mirko Scholz, Anastasiia A Klochkova, Armin de Meijere, Victor V Sokolov

**Affiliations:** 1 St. Petersburg State University, 7/9 Universitetskaya nab, St. Petersburg 199034, Russia; 2 Institut für Organische und Biomolekulare Chemie der Georg-August-Universität Göttingen, Tammannstrasse 2, D-37077 Göttingen, Germany

**Keywords:** arylation, fused-ring systems, indole formation, palladium catalysis, sultams

## Abstract

A new and efficient approach to five- and six-membered benzannelated sultams by intramolecular *C*-arylation of tertiary 1-(methoxycarbonyl)methanesulfonamides under palladium catalysis is described. In case of the α-toluenesulfonamide derivative, an unexpected formation of a 2,3-diarylindole was observed under the same conditions.

## Introduction

The sulfonamide functional group stands out as one of the most important pharmacophores. At the same time, cyclic sulfonamides (sultams) have also received significant attention due to their biological activities and medicinal uses [1–7]. On the other hand, especially fused sultams are among the most commonly used therapeutic agents owing to the broad spectrum of their activities [8–13]. Up to now, sultams have been prepared employing Diels–Alder reactions, radical cyclizations, reductions of sulfonylimines, ring-closing metatheses, nucleophilic aromatic substitutions and Heck cyclizations [14–16].

**Scheme 1:**
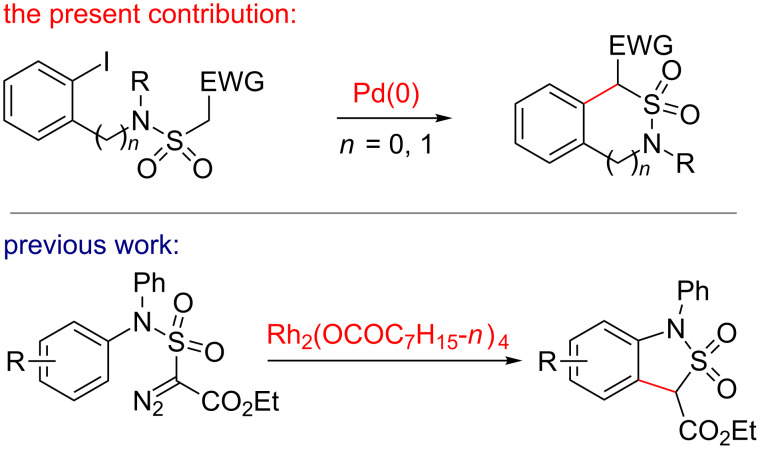
A previous and a new approach to arene-annelated sultams.

Earlier on, our group has developed versatile syntheses of sultams based on the transformation of methanesulfonamides bearing an additional α-acceptor (EWG) group [17–20]. Thus, intermolecular cyclodialkylation of α-substituted methanesulfonamides, upon treatment with α,ω-dibromoalkanes and bases, provides a rather simple and efficient route to five-, six- and seven-membered sultams. Moreover, bridged sultams with a pyramidal nitrogen atom and a sulfur atom in the apex positions were prepared from the same kind of starting materials in good to excellent yields [20]. Quite recently, Xu et al. employed 1-diazo-1-ethoxycarbonylmethanesulfonamides for the synthesis of benzo-γ-sultams. The key step in the latter transformation was a rhodium octanoate-catalyzed intramolecular carbenoid insertion into an *ortho* C_Ar_–H bond (Scheme 1) [21–22], which proceeded with good yields in most cases. However, with non-equivalent aryl *ortho*-positions in the starting diazo compounds, mixtures of regioisomers – virtually in 1:1 ratios – were always formed, which is a serious drawback of this approach. Another well-known method based on retrosynthetic disconnections at the same C–C bond employed intramolecular vicarious nucleophilic substitution of hydrogen in the substituted *N*-(3-nitrophenyl)chloromethylsulfonamides [23–24]. However, this method is limited to sultams bearing a strong electron-withdrawing group on the aromatic ring. A similar approach utilizes a nucleophilic aromatic substitution and leads to benzo-γ-sultams, yet this reaction requires harsh conditions and has a limited scope with only few examples [25–26]. More recently, Zard et al. have developed a sequence of lauroyl peroxide-catalyzed radical additions of xanthate to substituted *N*-aryl vinyl sulfonamides and subsequent intramolecular cyclization to yield benzo-annelated γ-sultams [27]. Quite interestingly, the obtained sultams were efficiently used for the preparation of *ortho*-functionalized anilines.

This contribution deals with a new access to ring-annelated sultams by an intramolecular Pd(0)-catalyzed arylation of tertiary sulfonamides bearing an additional C–H acidic center (Scheme 1). From several potentially suitable electron-withdrawing groups (EWG), e.g., alkoxycarbonyl, aryl, cyano, or trifluoromethyl, we have chosen methoxycarbonyl (CO_2_Me) and aryl (Ar), since the corresponding starting sulfonyl chlorides are the most easily accessible ones.

## Results and Discussion

**Scheme 2:**
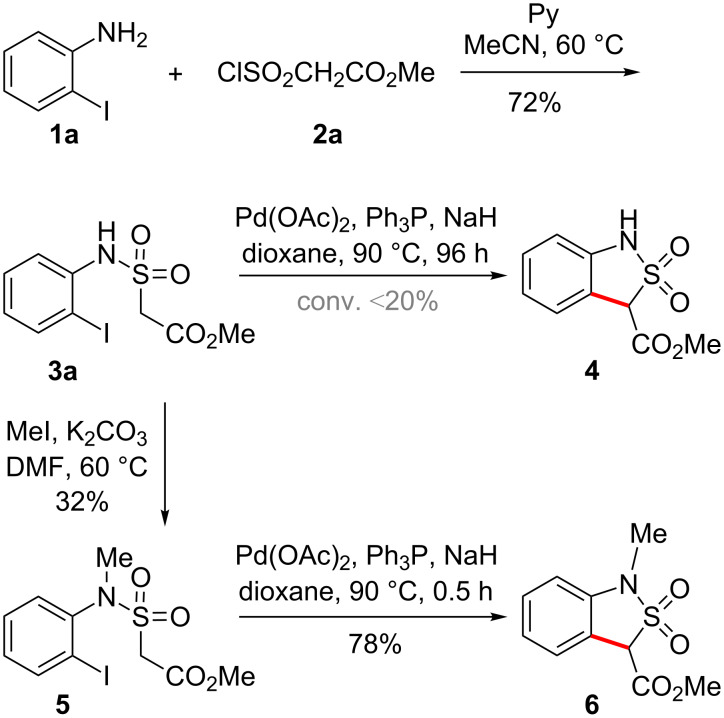
Pd-catalyzed cyclization of (2-iodophenyl)sulfonamides **3** and **5**.

To begin with, methyl 2-[*N*-(2-iodophenyl)sulfamoyl]acetate (**3a**) was prepared from commercially available 2-iodoaniline (**1a**) and methyl (chlorosulfonyl)acetate (**2a**) according to a previously published protocol (Scheme 2) [18–19].

The initial reaction conditions were chosen based on several reports on intermolecular arylations of “classic” weakly C–H acidic compounds under palladium catalysis [28–35]. Disappointingly, an attempted cyclization of **3a** employing one of the established precatalyst cocktails – Pd(OAc)_2_, Ph_3_P, NaH in dioxane – virtually failed. The target product **4** could be detected in the reaction mixture by ^1^H NMR spectroscopy, but the conversion of the starting material was less than 20% after 4 days, and **4** could not be isolated in pure form (Scheme 2). A possible reason for this failure could be the presence of the N–H acidic fragment in **3a**, and therefore the tertiary analogue **5** was prepared by *N*-alkylation of **3a** with methyl iodide (Scheme 2). Unfortunately, this apparently simple transformation yielded a mixture of *C*-, *N*- and *C*,*N*-methylation products due to similar reactivities of the *C*- and *N*-nucleophilic centers in **3a**. At best, the tertiary sulfonamide **5** was isolated in 32% yield. In contrast to the behavior of **3a**, the tertiary analogue **5** underwent cyclization smoothly and rapidly (1 h) under the same conditions to furnish the benzannelated sultam **6** in 78% yield (Scheme 2).

Thus, in order to enable the preparation of arene-annelated tertiary sultams of type **6** along this route, an effective and versatile access to the *N*-alkylated precursors of type **5** had to be found. In this context, the 4-methoxybenzyl (PMB) was chosen as a possible protective group for the nitrogen atom, since it can subsequently be removed under acidic [36–37] as well as oxidative conditions [38–39]. In view of the regioselectivety problem in the alkylation of the sulfonamide **3a**, the sequence of steps was reversed. Thus, 2-iodo-*N-*(4-methoxybenzyl)anilines **7a**–**g** were prepared by reduction of the Schiff bases obtained from 2-iodoanilines **1a**–**g** and 4-methoxybenzaldehyde with sodium cyanoborohydride in a mixture of glacial acetic acid and acetonitrile according to a previously published protocol (for more details see Supporting Information File 1) [40–42].

**Scheme 3:**
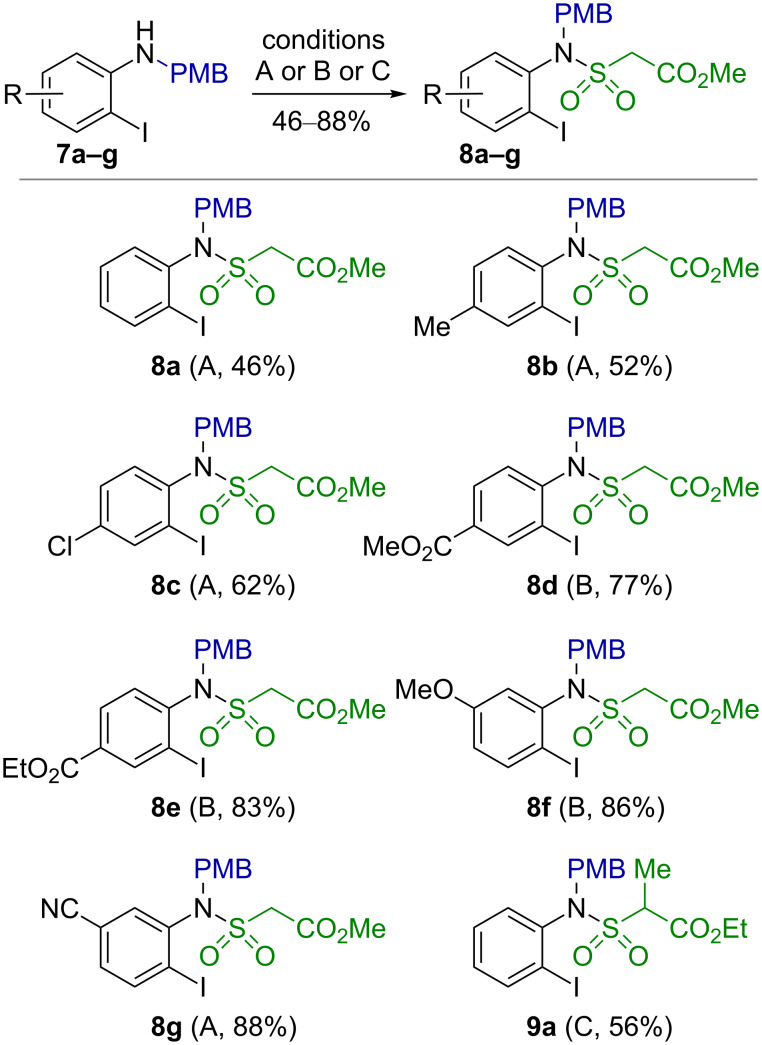
Preparation of 4-methoxybenzyl-protected methyl 2-(*N*-*o*-iodoarylsulfamoyl)acetates **8**. Reagents and conditions A: MeO_2_CCH_2_SO_2_Cl (**2a**), Py, MeCN, 60 °C, 48 h; conditions B: MeO_2_CCH_2_SO_2_Cl (**2a**), PhNEt_2_, CH_2_Cl_2_, rt, 20 h; conditions C: EtO_2_CCH(Me)SO_2_Cl (**2b**), Py, MeCN, 60 °C, 48 h.

Surprisingly, the obtained 2-iodo-*N*-(4-methoxybenzyl)aniline (**7a**) turned out to be rather sluggish in its reaction with the sulfonyl chloride **2**. Previously used conditions viz. carrying out the reaction in MeCN in the presence of pyridine at rt gave poor results [17–20]. Thus, in case of aniline **7a** the desired sulfonamide **8a** was obtained in only 7% yield. Employment of a stronger base such as triethylamine in various solvents (MeCN, DMF, CH_2_Cl_2_, THF) led to substantial decomposition of the sulfonyl chloride **2a** and did not improve the yield of the target product. However, sulfonylation of **7a** can be achieved with two equivalents of **2a** in MeCN in the presence of pyridine at 60 °C for 2 days to give the sulfonamide **8a** in an acceptable yield of 46%. Employing these conditions, methyl 2-[*N*-(2-iodo-4-methylphenyl)-*N*-(4-methoxybenzyl)sulfamoyl]acetate (**8b**) and methyl 2-[*N*-(4-chloro-2-iodophenyl)-*N*-(4-methoxybenzyl)sulfamoyl]acetate (**8c**) could be prepared in 52 and 62% yield, respectively (Scheme 3). In a recent paper Xu et al. report sulfonylation of sluggish anilines with sulfonyl chloride **2a** should be performed in the presence of 2 equivalents of aniline without an additional base [21]. However, we figured out that the second equivalent of aniline could efficiently be replaced with an inexpensive weak base such as *N,N-*diethylaniline and under such conditions obtained the sulfonamides **8d–g** in good yields (Scheme 3).

Next, the key step in the targeted synthesis, the intramolecular Pd-catalyzed arylation was optimized for the sulfonamide **8a** (Table 1).

**Table 1:** Optimization of the reaction conditions for the Pd-catalyzed cyclization of the sulfonamide **8a**.

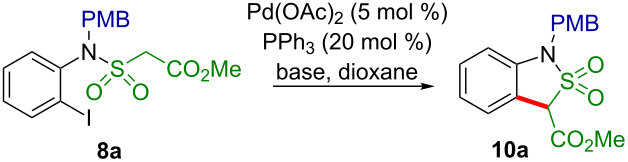		

entry	base (equiv)	conditions^a^ (yield/conversion %)^b^

1	NaH (4.0)	90 °C, 1 h (89/100)
2	*t*-BuOK (4.0)	90 °C, 1 h (49/94)
3	MeONa (4.0)	90 °C, 1 h (44/80)
4	K_2_CO_3_ (4.0)	90 °C, 1 h (0/16)
5^c^	K_2_CO_3_ (4.0)	90 °C, 1 h (10/58)
6	NaH (2.5)	90 °C, 1 h (48/52)
7^d^	NaH (4.0)	90 °C, 1 h (4/28)
8	NaH (4.0)	90 °C, 30 min (98/100)
9	NaH (4.0)	90 °C, 15 min (91/96)
10	NaH (4.0)	25 °C, 1 h (0/10)

^a^Reactions were performed with 0.25 mmol of **8a**. ^b^Yield and conversion were estimated on the basis of ^1^H NMR spectra (1,2-diphenylethane was used as an internal standard). ^c^Reaction performed in DMAA. ^d^1 mol % and 4 mol % of Pd(OAc)_2_ and Ph_3_P, respectively, were used.

Initially the studied reaction was performed in dioxane at 90 °C employing 5 mol % of Pd(OAc)_2_, 20 mol % of Ph_3_P, and 4.0 equivalents of NaH. Although full conversion of **8a** was achieved and the NMR-determined yield of sultam **10a** was 89% (Table 1, entry 1), attempts were made to further improve the reaction conditions by testing different bases such as potassium *tert*-butoxide and sodium methoxide in dioxane and potassium carbonate in dioxane as well as in DMAA (Table 1, entries 2–5). Unfortunately, in all cases the yields of the target sultam **10a** were poorer. Since NaH in dioxane had given the best results, various parameters of these conditions were tested. With a reduced amount of NaH (2.5 instead of 4.0 equiv) the conversion was lower and so was the yield. With a lower amount of the precatalyst (1 mol % of Pd(OAc)_2_ and 4 mol % of Ph_3_P) the sultam **10a** was formed in an extremely low yield of 4% (Table 1, entry 7). Finally, when keeping the mixture at 90 °C for only 30 min instead of 1 h, a virtually quantitative transformation of **8a** to the sultam **10a** was achieved (Table 1, entries 8, 9). To demonstrate the scalability of this synthesis of the sultam **10a**, the reaction was carried out with 7.1 g of **8a** and just 2 mol % of Pd(OAc)_2_, to furnish an isolated yield of 4.3 g (83%) of the sultam **10a**.

**Scheme 4:**
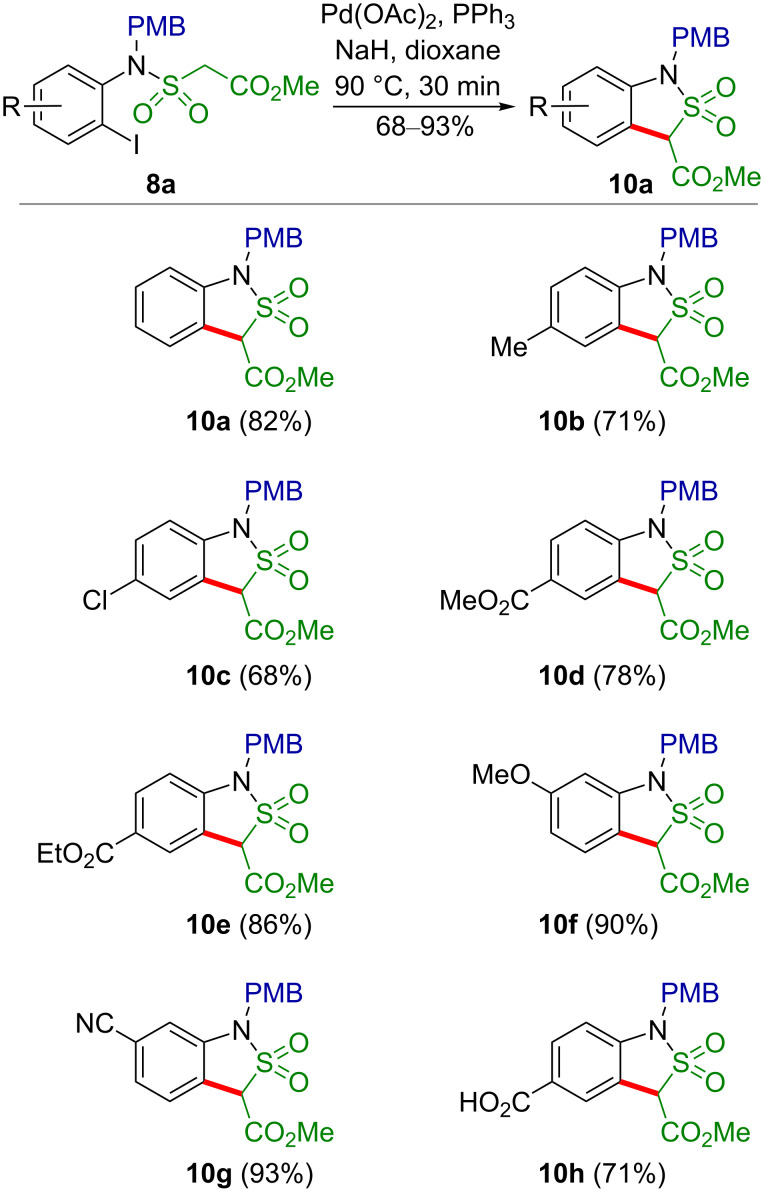
Synthesis of arene-annelated sultams **10** by Pd-catalyzed intramolecular arylation of a C–H acidic methylene group.

Under the optimized reaction conditions the whole series of sulfonamides **8b**–**g** was converted to the corresponding sultams **10b**–**g** in good to excellent yields (Scheme 4).

**Figure 1:**
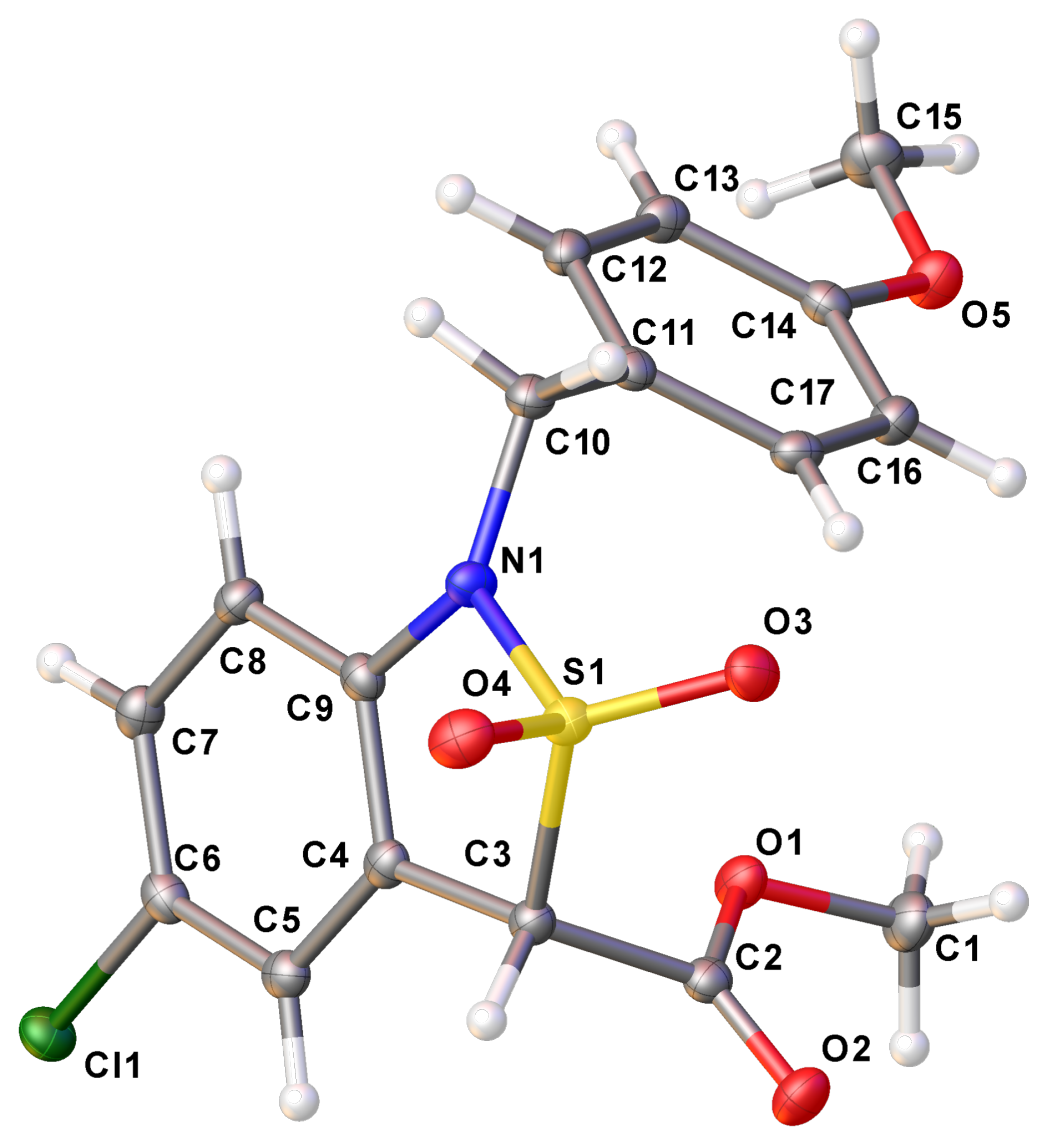
Structure of methyl 5-chloro-1-(4-methoxybenzyl)-1,3-dihydrobenzo[*c*]isothiazole-3-carboxylate-2,2-dioxide (**10c**) in the crystal [CCDC: 1520988].

No significant differences between the unsubstituted sulfonamide **8a**, and its derivatives bearing weak electron-donating (**8b**) and strong electron-withdrawing (**8d**,**e**) groups in the *para*-position relative to the nitrogen atom were detected. However, methyl 2-(*N*-(5-cyano-2-iodophenyl)-*N*-(4-methoxybenzyl)sulfamoyl)acetate (**8g**) and methyl 2-(*N*-(2-iodo-5-methoxyphenyl)-*N*-(4-methoxybenzyl)sulfamoyl)acetate (**8f**) behaved slightly differently. Sulfonamide **8g** was completely converted to the corresponding sultam **10g** within 5 min (checked by TLC), whereas **8f** underwent cyclization significantly more slowly than the unsubstituted analog **8a**, and was fully converted after 3 h at 90 °C. When the esters **8d**,**e** were treated with the precatalyst under the standard conditions in freshly distilled dioxane, the sultams **10d** and **10e** were isolated in 78 and 86% yield, respectively. However, when commercial dioxane was used, both sulfonamide **8d** as well as **8e** gave the same product, namely the benzoic acid derivative **10h** in 65 and 71% yield, respectively. Apparently, the commercial dioxane contained a certain amount of water, so that the benzoates **10d**,**e** underwent hydrolysis. Indeed, when the sultam **10e** was treated with sodium hydride in freshly distilled dioxane followed by addition of a small amount of water, the acid **10h** was isolated in an excellent yield of 93%. The relative stability of the methoxycarbonyl group at C-3 under these basic conditions must be due to its reduced electrophilicity in connection with the facile enolate formation at this position. The structures of all obtained compounds were assigned on the basis of ^1^H and ^13^C NMR spectra, as well as high resolution mass spectrometric data. The structure of the sultam **10c** was unequivocally confirmed by a single-crystal X-ray diffraction study (Figure 1).

While the intramolecular arylation of the C–H acidic CH_2_ group in sulfamoylacetates **8a–g** to yield 1,3-dihydrobenzo[*c*]isothiazole-2,2-dioxide derivatives **10a**–**h** works well, the sulfonamide **9a** with a tertiary C–H acidic group, which was prepared from the 2-iodoaniline derivative **7a** and ethyl 2-(chlorosulfonyl)propanoate (**2b**) according to the protocol employed for the sulfonamides **8a–c**, under the same conditions did not yield the corresponding cyclization product, but a complex and inseparable mixture.

**Scheme 5:**
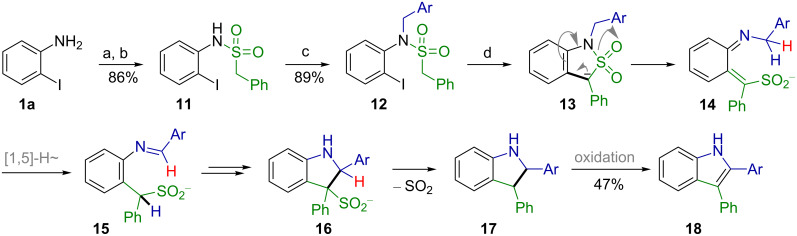
Palladium-catalyzed transformation of *N*-(2-iodophenyl)-*N*-(4-methoxybenzyl-benzylsulfonamide **12**. Ar = 4-MeOC_6_H_4_; a) BnSO_2_Cl, NMM, CH_2_Cl_2_, rt, 0.5 h; b) MeONa, MeOH, reflux, 3 h; c) 4-MeOC_6_H_4_CH_2_Cl, K_2_CO_3_, DMF, 60 °C, 2 h; d) Pd(OAc)_2_, Ph_3_P, NaH, dioxane, 70 °C, 30 min.

To further test the scope and limitations of this cyclization with respect to other C–H acidifying groups (EWG) on the tether, the α-toluenesulfonamide **12** was prepared in two steps from *o*-iodoaniline (**1a,** Scheme 5). The subsequent sulfonylation of **1a** with α-toluenesulfonyl chloride yielded a mixture of the sulfonamide **11** along with a side product resulting from twofold *N*-sulfonylation. Gratifyingly, subsequent treatment of the reaction mixture with a solution of sodium methoxide in methanol converted this side product to **11**, so that the overall yield of the latter was 86%. Alkylation of **11** with 4-methoxybenzyl chloride in DMF in the presence of potassium carbonate according to a previously published protocol [17] gave the *N*-PMB-protected tertiary sulfonamide **12** in 89% yield. The attempted palladium-catalyzed cyclization of **12** under the conditions employed above (Scheme 4), yet at 70 °C did not give **13**, but the unexpected 2,3-diarylindole **18** in 47% isolated yield (Scheme 5).

The formation of **18** can best be rationalized assuming a ring opening of the initially formed anionic cyclization product **13** to furnish the aza-*ortho*-quinodimethane sulfonyl anion **14** [43]. The formation of **14** can also proceed as a cheletropic extrusion of SO_2_ from **13** followed by recombination of SO_2_ with the formed carbanionic center. Similar fragmentations of such 1,3-dihydro-2,1-benzothiazol-2,2-dioxides and related compounds are known, but usually require heating to above 200 °C to yield aza-*ortho*-quinodimethane derivatives [44], which can rearrange into the corresponding Schiff bases by sigmatropic [1,5]-hydrogen shift [45]. In the case of **13**, the extrusion of SO_2_ must be drastically accelerated due to its carbanionic nature, so that it can occur at 70 °C. The resulting anionic aza-*ortho*-quinodimethane intermediate **14** then, by [1,5]-hydrogen shift gives the *ortho*-benzylaniline-derived Schiff base **15**, which undergoes cyclization to give the indoline derivative **16**, and this by subsequent elimination of SO_2_ and protonation yields **17**. The latter, like many indolines [46–48] probably is susceptible to very rapid oxidation upon work-up in air to give the corresponding indole **18**.

**Scheme 6:**
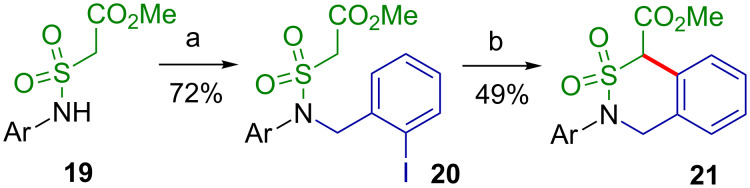
Palladium-catalyzed intramolecular arylation to yield a benzannelated six-membered sultam **21**. Ar = 4-MeOC_6_H_4_; a) 2-IC_6_H_4_CH_2_OH, Ph_3_P, DEAD, THF, rt, 12 h; b) Pd(OAc)_2_, Ph_3_P, NaH, dioxane, 90 °C, 20 h.

In order to test the applicability of the palladium-catalyzed intramolecular arylation of a sulfonamide with a C–H acidic group for the preparation of benzannelated six-membered sultams like **21**, the appropriate precursor **20** was prepared in one step from the respective sulfonamide **19** and 2-iodobenzyl alcohol employing a Mitsunobu protocol (Scheme 6) [49].

Treatment of **20** with palladium acetate, Ph_3_P, and sodium hydride in dioxane at 90 °С for 20 h furnished the target sultam **21** in 49% yield. Apparently, the cyclization of **20** occurs significantly more slowly than those of the sulfonamides **8a–g**, which is in accordance with the well-known fact that an intermediate six-membered palladacycle forms more rapidly than a seven-membered analogue in similar transformations.

**Scheme 7:**
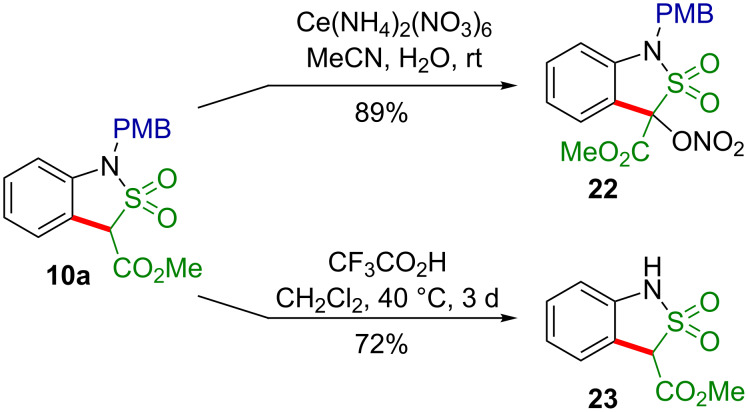
An attempted and a successful removal of the PMB group from the sultam **10a**.

**Figure 2:**
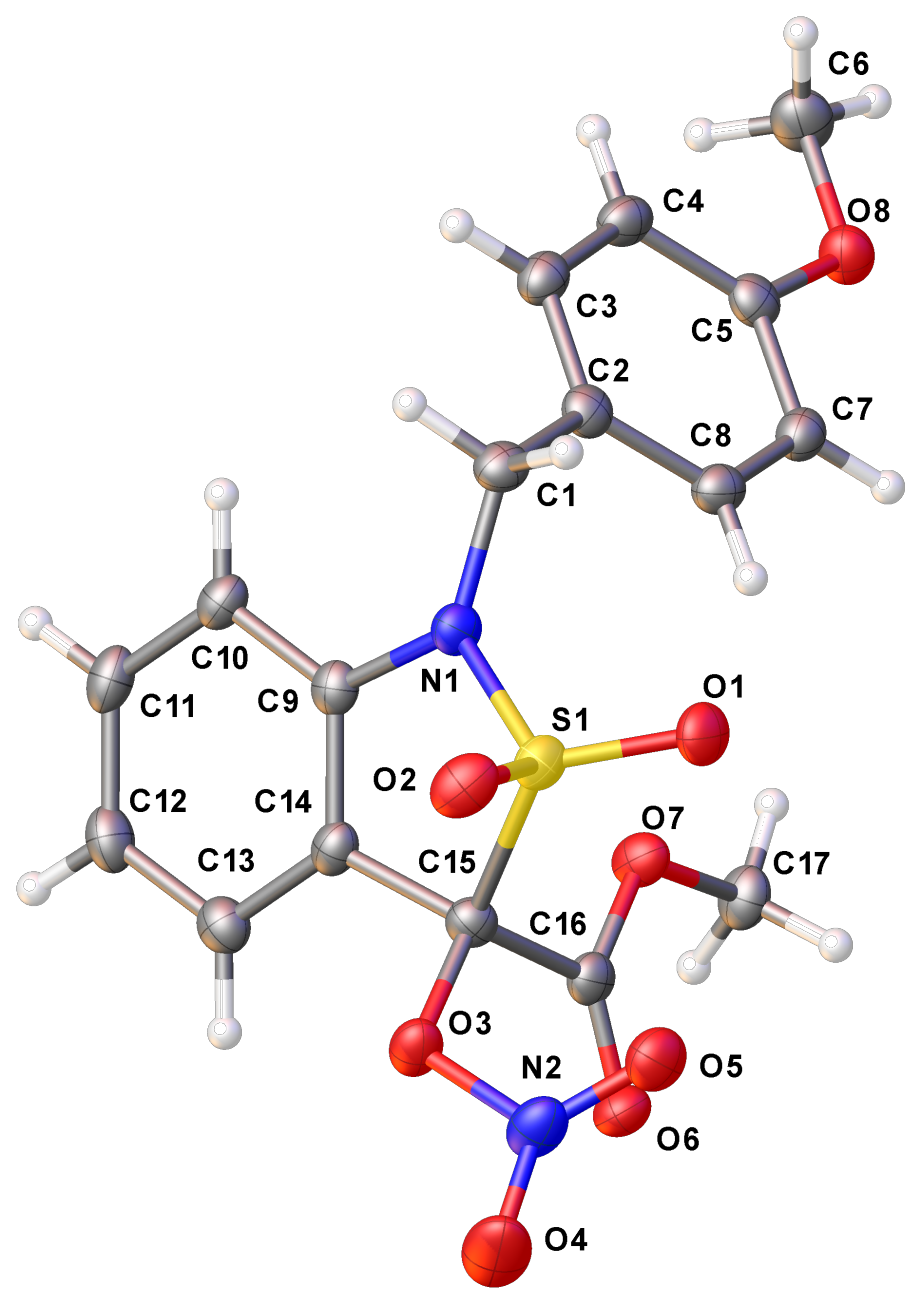
Structure of methyl 1-(4-methoxybenzyl)-3-(nitrooxy)-1,3-dihydrobenzo[c]isothiazole-3-carboxylate-2,2-dioxide (**22**) in the crystal [CCDC: 1520989].

In an attempt to remove the *p*-methoxybenzyl group (PMB) from the nitrogen, the methyl 1-(4-methoxybenzyl)-1,3-dihydrobenzo[*c*]isothiazole-3-carboxylate-2,2-dioxide (**10a**) was treated with cerium(IV) ammonium nitrate (CAN) according to a previously developed protocol [18,20]. Unexpectedly, this PMB resisted its removal, and the only formed product was the nitrooxy derivative **22**, presumbaly via the corresponding benzyl-type free radical (Scheme 7). The structure of **22** was proven by single-crystal X-ray diffraction (Figure 2).

According to a publication by Metz et al., a *N*-(4-methoxybenzyl) group can be removed from sultams by treatment with trifluoroacetic acid [36]. Indeed, when **10a** was treated with CF_3_CO_2_H in refluxing dichloromethane for 3 days, the deprotected sultam **23** was isolated in a good yield of 72% (Scheme 7).

## Conclusion

In conclusion, the intramolecular palladium-catalyzed *C*-arylation of tertiary 1-(methoxycarbonyl)methanesulfonanilides can be considered as a viable approach to five- and six-membered benzannelated sultams. The 4-methoxybenzyl group is a suitable protective group for the sultam synthesis since it is stable under the reaction conditions and can be easily removed by acidolysis with trifluoroacetic acid.

## Supporting Information

File 1 Experimental procedure and analytical data.
