# Supplementary material for: Synthesis of benzannelated sultams by intramolecular Pd-catalyzed arylation of tertiary sulfonamides
Source: Beilstein J Org Chem. 2017 Sep 12;13:1932–9. doi: 10.3762/bjoc.13.187 (PMC5629375; doi:10.3762/bjoc.13.187)
Supplement: File 1 — Experimental procedure and analytical data. [file Beilstein_J_Org_Chem-13-1932-s001.pdf]

Supporting Information

for

Synthesis of benzannelated sultams by intramolecular Pd-catalyzed arylation of tertiary sulfonamides

Valentin A. Rassadin[1], Mirko Scholz[1], Anastasiia A. Klochkova[1], Armin de Meijere[*2], Victor V. Sokolov[*1]

Address: [1]St. Petersburg State University, 7/9 Universitetskaya nab, St. Petersburg, 199034 Russia and [2]Institut für Organische und Biomolekulare Chemie der Georg-August-Universität Göttingen, Tammannstrasse 2, D-37077 Göttingen, Germany.

Email: Victor V. Sokolov[*] - vsokolo@mail.ru; Armin de Meijere[*] - ameijer1@gwdg.de

[*]Corresponding author

Table of contents

1 General remarks S2
2 Experimental procedures S3
2.1. Synthesis of methyl 2-[*N*-(2-iodophenyl)-*N*-methylsulfamoyl]acetate (**5**) S3
2.2. Synthesis of *o*-iodoanilines **1** S4
2.3. Synthesis of *N*-PMB substituted *o*-iodoanilines S5
2.4. Synthesis of *N*-PMB-protected α-substituted methanesulfonamides S8
2.5. General procedure for Pd-catalyzed cyclization of *N*-alkyl-*N*-arylsulfonamides (**5**, **8a**–**g**) (**GP3**) S14
2.6. Synthesis of methyl 3-(4-methoxyphenyl)-1*H*-3,4-dihydrobenzo[d][1,2]thiazine-1-carboxylate-2,2-dioxide (**21**) S18
2.7. Miscellaneous transformations S19
3 NMR spectra S22
3.1. ^{1}H and ^{13}C NMR spectra of *N*-PMB substituted *o*-iodoanilines S22
3.2. ^{1}H and ^{13}C NMR spectra of sulfonamides S27
3.3. ^{1}H and ^{13}C NMR spectra of sultams S40
4 X-ray crystallographic data S53
4.1. Structure analysis of crystal of methyl 5-chloro-1-(4-methoxybenzyl)-1,3-dihydrobenzo[c]isothiazole-3-carboxylate-2,2-dioxide (**10c**) S53
4.2. Structure analysis of crystal of methyl 1-(4-methoxybenzyl)-3-(nitrooxy)-1,3-dihydrobenzo[c]isothiazole-3-carboxylate-2,2-dioxide (**22**) S55

1 General remarks

NMR spectra were recorded at ambient temperature with a Bruker DPX 300 instrument at 300.13 (^{1}H) and 75.47 (^{13}C, DEPT-135) MHz; a Bruker Avance 400 instrument at 400.13 MHz (^{1}H NMR) and 100.61 MHz (^{13}C NMR, DEPT-135); Bruker Avance III 500 instrument at at 125.73 MHz (^{13}C NMR and DEPT-135). Chemical shifts (δ) are given in ppm relative to resonances of solvents (^{1}H: δ = 7.26 for the residual $CHCl_3$ peak, δ = 2.50 for the residual DMSO-d_5 peak; ^{13}C: δ = 77.0 for $CDCl_3$, δ = 39.5 for DMSO-d_6). Spin-spin coupling constants (J) are given in Hz. Multiplicities of signals are described as follows: s = singlet, d = doublet, t = triplet, q = quartet, m = multiplet, br = broad. The multiplicities of signals in ^{13}C NMR spectra were determined by the DEPT-135 and APT technique. Mass spectra were recorded with a Finnigan MAT 95 (EI), a Bruker MicroTOF (ESI), and an Agilent 6538 UHD instrument. Analytical TLC was performed on unmodified Merck ready-to-use plates (TLC silica gel 60 F254). Detection was achieved with a UV lamp or by development with molybdatophosphoric acid solution (5% in EtOH). Melting points were determined in capillaries with a Stuart SMP 30 apparatus.

Methyl 2-(chlorosulfonyl)acetate (**2a**),[1] ethyl 2-(chlorosulfonyl)propionate (**2b**),[2] phenylmethanesulfonyl chloride (**2c**),[2] 2-iodo-*N*-(4-methoxybenzyl)-4-methylaniline (**7b**),[3] and 4-chloro-2-iodo-*N*-(4-methoxybenzyl)aniline (**7c**)[3] were previously prepared in our research group. 2-Iodoaniline (**1a**), 1-iodo-4-methoxy-2-nitrobenzene, and (2-iodophenyl)methanol were purchased from ABCR GmbH. 4-Iodo-3-nitrobenzonitrile was purchased from FluoroChem Ltd. Ethyl 4-amino-3-iodobenzoate (**1e**) was kindly donated by Dr. Alexandra Kulyashova.[4]

Crystal structure analyses:

Crystals suitable for X-ray diffractometry of compounds **9c** and **22** were obtained by slow evaporation of their solutions in hexane/EtOAc and EtOAc respectively.

X-ray diffraction data were collected at an Xcalibur Eos diffractometer at 100 K using Cu-Kα (λ = 0.154184 nm) radiation. The structures were solved by direct methods using the SHELXS and refined with the SHELXL[5] incorporated in the OLEX2 program package.[6]

---

[1] a) Rassadin, V. A.; Tomashevskiy, A. A.; Sokolov, V. V.; Poteknin, A. A. *Khimiya Geterotsiklicheskikh Soedinenii*, **2008**, 605–617, *Chem. Heterocycl. Compd.* **2008**, *44*, 474–485.
[2] Rassadin, V. A.; Grosheva, D. S.; Arefeva, I. A.; Tomashevskiy, A. A.; Sokolov, V. V.; de Meijere, A. *Eur. J. Org. Chem.* **2012**, *2012*, 5028–5037.
[3] Grosheva, D. S.; Rassadin, V. A.; Sokolov, V. V. *European J. Org. Chem.* **2015**, 1355–1363.
[4] Kulyashova, A. E.; Mikheeva, E. V.; Danilkina, N. A.; Balova, I. A. *Mendeleev Comm.* **2014**, *24*, 102–104.
[5] G. M. Sheldrick, *Acta Crystallogr. Sect. A.* **2008**, *64*, 112–122.
[6] Dolomanov, O. V.; Bourhis, L. J.; Gildea, R. J.; Howard, J. A. K.; Puschmann, H. *J. Appl. Cryst.*, **2009**, *42*, 339–341.

2 Experimental procedures

2.1. *Synthesis of methyl 2-[N-(2-iodophenyl)-N-methylsulfamoyl]acetate (**5**)*

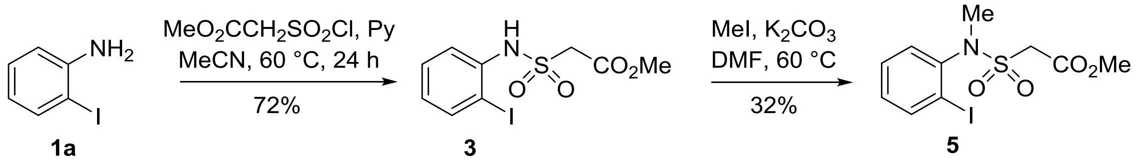

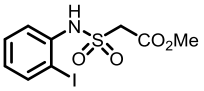

Methyl 2-[*N*-(2-iodophenyl)sulfamoyl]acetate (3): A solution of methyl (chlorosulfonyl)acetate (**2a**) (5.18 g, 30.0 mmol) in anhydrous MeCN (30.0 mL) was slowly added to a stirred solution of 2-iodaniline (**1a**) (7.23 g, 33.0 mmol) and pyridine (2.84 g, 35.9 mmol) in anhydrous MeCN (75 mL) at 10 °C within 30 min. The reaction mixture was then warmed up to 30 °C and stirred for another 1 h. Water (250 mL) was then added, the mixture was acidified with conc. HCl to pH 2, and extracted with CH_2Cl_2 (3 × 75 mL). The combined organic fractions were washed with 5% HCl (2 × 50 mL), brine (50 mL), and dried over anhydrous Na_2SO_4. The solvents were removed, and the crude product was purified by recrystallization from a mixture of Et_2O and hexane to give **3** (8.44 g, 72%) as a colorless solid, m.p. 99–100 °C. **^{1}H NMR** (400 MHz, $CDCl_3$): δ = 3.77 (s, 3 H, OCH_3), 4.10 (s, 2 H, SO_2CH_2), 6.92 (td, *J* = 1.5, 7.8 Hz, 1 H, H-Ar), 7.07 (br. s, 1 H, NH), 7.36 (td, *J* = 1.5, 7.8 Hz, 1 H, H-Ar), 7.66 (dd, *J* = 1.4, 8.2 Hz, 1 H, H-Ar), 7.83 (dd, *J* = 1.4, 8.0 Hz, 1 H, H-Ar) ppm. **^{13}C NMR** (100 MHz, $CDCl_3$): δ = 53.3 (OCH_3), 55.4 (SO_2CH_2), 91.1 (C-Ar), 121.1 (CH-Ar), 127.1 (CH-Ar), 129.8 (CH-Ar), 137.2 (C-Ar), 139.7 (CH-Ar), 163.0 (CO) ppm. **HRMS** (ESI), *m/z*: $[M + H]^+$ calcd. for $C_9H_{11}INO_4S^+$: 355.9448; found: 355.9443.

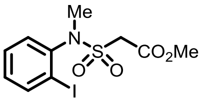

Methyl 2-[*N*-(2-iodophenyl)-*N*-methylsulfamoyl]acetate (5): A solution of MeI (740 mg, 5.21 mmol) in DMF (30 mL) was added dropwise to a mixture of sulfonamide **3** (1.59 g, 4.48 mmol), K_2CO_3 (1.00 g, 7.25 mmol) and DMF (30 mL) at 60 °C within 1 h. The reaction mixture was then stirred at 60 °C for 1 h, DMF was removed, and the residue was carefully dissolved in a mixture of CH_2Cl_2 (40 mL) and 5% HCl (30 mL). The organic phase was separated and washed with 3% aq. HCl (3 × 30 mL), water (30 mL), brine (30 mL), dried over Na_2SO_4 and concentrated. The crude product was purified by recrystallization from a mixture of Et_2O and hexane to give **5** (529 mg, 32%) as a colorless solid, m.p. 93–95 °C. **^{1}H NMR** (300 MHz, $CDCl_3$): δ = 3.30 (s, 3 H, NCH_3), 3.84 (s, 3 H, OCH_3), 4.17 (s, 2 H, SO_2CH_2), 7.08 (td, *J* = 1.2, 7.8 Hz, 1 H, H-Ar), 7.41 (td, *J* = 1.2, 7.8 Hz, 1 H, H-Ar), 7.56 (dd, *J* = 1.2, 7.9 Hz, 1 H, H-Ar), 7.93 (dd, *J* = 1.2, 7.9 Hz, 1 H, H-Ar) ppm. **^{13}C NMR** (75 MHz, $CDCl_3$): δ = 39.8 (NCH_3), 53.1 (OCH_3), 56.9 (SO_2CH_2), 100.9 (C-Ar), 129.7 (CH-Ar), 130.2 (CH-Ar), 130.4 (CH-Ar), 140.5 (CH-Ar), 142.9 (C-Ar), 163.7 (CO) ppm. **HRMS** (ESI), *m/z*: $[M + Na]^+$ calcd. for $C_{10}H_{12}INNaO_4S^+$: 391.9424; found: 391.9424.

2.2. Synthesis of o-iodoanilines ***1***

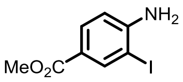

Methyl 4-amino-3-iodobenzoate (1e): From methyl 4-aminobenzoate (15.1 g, 99.9 mmol), iodine (15.2 g, 60 mmol), and 30% aq. H_2O_2 (12.2 mL), compound **1e** (13.9, 50%) was obtained according to a previously published protocol[7] as a colorless solid, m.p. 87–89 °C, lit.[8] m.p. 84–87 °C.

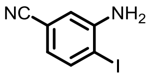

3-Amino-4-iodobenzonitrile (1g): From 4-iodo-3-nitrobenzonitrile (13.7 g, 50.0 mmol), iron powder (8.38 g, 150 mmol), and NH_4Cl (13.4 g, 250 mmol) in 70% (v/v) aq. EtOH (150 mL), compound **1g** (10.2, 84%) was obtained according to a previously published protocol[9] as a colorless solid, m.p. 114–115 °C, lit.[10] m.p. 119–121 °C.

---

[7] Li, F. N.; Kim, N. J.; Nam, Y. H.; Kim, S. H.; Seo, S. Y.; Jeong, Y. S.; Kim, S. Y.; Park, Y. H.; Suh, Y. G. *Arch. Pharm. Res.* **2009**, *32*, 1201–1210.
[8] Hill, M. L.; Raphael, R. A. *Tetrahedron* **1990**, *46*, 4587–4594.
[9] Barton, J. W.; Lapham, D. J.; Rowe, D. J. *J. Chem. Soc. Perkin. Trans. 1* **1985**, *1985*, 131–133.
[10] Zoraghi, R.; Campbell, S.; Kim, C.; Dullaghan, E. M.; Blair, L. M.; Gillard, R. M.; Reiner, N. E.; Sperry, J. *Bioorg. Med. Chem. Lett.* **2014**, *24*, 5059–5062.

2.3. *Synthesis of N-PMB substituted o-iodoanilines*

General procedure for the benzylation of *o*-iodoanilines (**GP1**)

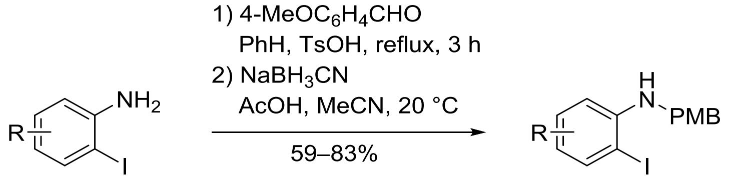

A solution of the respective aniline **1** (100 mmol), 4-methoxybenzaldehyde (13.6 g, 100 mmol), and TsOH (5 mg, 30 µmol) in benzene (120 mL) was heated under reflux with a Dean–Stark trap for 3 h. Benzene was then removed on a rotary evaporator and the residue was dissolved in a mixture of MeCN (40 mL) and glacial AcOH (20 mL). The resulting solution was cooled to 0 °C, $NaBH_3CN$ (2.52 g, 40.1 mmol) was then added gradually at 5–10 °C within 10 min, and stirring was continued for 1 h. The reaction mixture was worked up in two different ways.

a) The reaction mixture was diluted with cold water (150 mL), and the formed precipitate was filtered off, washed with water (200 mL) and dissolved in CH_2Cl_2 (150 mL). The resulting solution was washed with water (100 mL), 5% NaOH (3 × 35 mL), brine (50 mL) and dried over Na_2SO_4. The solvents were removed, and the crude product was purified by recrystallization from a mixture of EtOAc and hexane.

b) The reaction mixture was concentrated, and the residue was taken up in CH_2Cl_2 (150 mL). The resulting suspension was washed with water (100 mL), the 5% NaOH (3 × 35 mL), brine (50 mL) and finally dried over Na_2SO_4. The solvents were removed, and the crude product was purified by column chromatography (eluent: hexane/EtOAc, gradient from 0 to 40% of EtOAc).

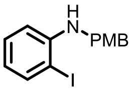

2-Iodo-*N*-(4-methoxybenzyl)aniline (7a): From **1a** (21.9 g, 100 mmol) and 4-methoxybenzaldehyde (13.6 g, 100 mmol), compound **7a** (28.2 g, 83%) was obtained according to **GP1a** as a colorless solid, m.p. 89–90 °C, lit.[11] m.p. = 89–90 °C. **^{1}H NMR** (400 MHz, $CDCl_3$): δ = 3.81 (s, 3 H, OCH_3), 4.33 (s, 2 H, NCH_2), 4.57 (br. s, 1 H, NH), 6.45 (td, *J* = 1.5, 7.6 Hz, 1 H, H-Ar), 6.56 (dd, *J* = 1.4, 8.2 Hz, 1 H, H-Ar), 6.88–6.92 (m, 2 H, H-Ar), 7.17 (td, *J* = 1.5, 7.6 Hz, 1 H, H-Ar), 7.27–7.31 (m, 2 H, H-Ar), 7.68 (dd, *J* = 1.5, 7.8 Hz, 1 H, H-Ar) ppm. **^{13}C NMR** (100 MHz, $CDCl_3$): δ = 47.8 (NCH_2), 55.2 (OCH_3), 85.3 (C-I), 110.9 (CH-Ar), 114.1 (2 × CH-Ar), 118.7 (CH-Ar), 128.4 (2 × CH-Ar), 129.4 (CH-Ar), 130.5 (C-Ar), 138.9 (CH-Ar), 147.1 (C-Ar),

[11] Hiroya, K.; Itoh, S.; Sakamoto, T. *J. Org. Chem.* **2004**, *69*, 1126–1136.

158.9 (C-Ar) ppm. **HRMS** (ESI), *m/z*: $[M + Na]^+$ calcd. for $C_{14}H_{14}INNaO^+$: 362.0012; found: 362.0006.

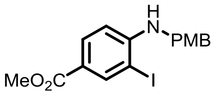

Methyl 3-iodo-4-((4-methoxybenzyl)amino)benzoate (7d): From **1d** (2.50 g, 9.02 mmol) and 4-methoxybenzaldehyde (1.23 g, 9.03 mmol), compound **7d** (2.10 g, 59%) was obtained according to **GP1a** as a colorless solid, m.p. 87–88 °C. **^{1}H NMR** (400 MHz, $CDCl_3$): δ = 3.81 (s, 3 H, OCH_3), 3.84 (s, 3 H, OCH_3), 4.37 (d, *J* = 5.3 Hz, 2 H, NCH_2), 5.00 (t, *J* = 5.3 Hz, 1 H, NH), 6.51 (d, *J* = 8.6 Hz, 1 H, H-Ar), 6.88–6.92 (m, 2 H, H-Ar), 7.24–7.28 (m, 2 H, H-Ar), 7.85 (dd, *J* = 8.6, 1.9 Hz, 1 H, H-Ar), 8.36 (d, *J* = 1.9 Hz, 1 H, H-Ar) ppm. **^{13}C NMR** (100 MHz, $CDCl_3$): δ = 47.5 (NCH_2), 51.7 (OCH_3), 55.3 (OCH_3), 83.6 (C-I), 109.3 (CH-Ar), 114.2 (2 × CH-Ar), 120.0 (C-Ar), 128.5 (2 × CH-Ar), 129.5 (C-Ar), 131.4 (CH-Ar), 140.7 (CH-Ar), 150.3 (C-Ar), 159.1 (C-Ar), 165.9 (CO) ppm. **HRMS** (ESI), *m/z*: $[M + Na]^+$ calcd. for $C_{16}H_{16}INNaO_3^+$: 420.0067; found: 420.0052.

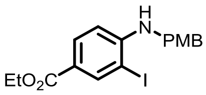

Ethyl 3-iodo-4-((4-methoxybenzyl)amino)benzoate (7e): From **1e** (2.65 g, 9.10 mmol) and 4-methoxybenzaldehyde (1.24 g, 9.11 mmol), compound **7e** (2.79 g, 75%) was obtained according to **GP1b** as a colorless oil, which spontaneously crystallized in when stored a freezer, R_f = 0.46 (hexane/EtOAc, 2:1), m.p. 47–48 °C. **^{1}H NMR** (400 MHz, $CDCl_3$): δ = 1.36 (t, *J* = 7.1 Hz, 3 H, CH_2CH_3), 3.81 (s, 3 H, OCH_3), 4.31 (q, *J* = 7.1 Hz, 2 H, OCH_2), 4.38 (d, *J* = 5.3 Hz, 2 H, NCH_2), 4.99 (t, *J* = 5.3 Hz, 1 H, NH), 6.51 (d, *J* = 8.6 Hz, 1 H, H-Ar), 6.88–6.92 (m, 2 H, H-Ar), 7.24–7.28 (m, 2 H, H-Ar), 7.86 (dd, *J* = 8.6, 1.8 Hz, 1 H, H-Ar), 8.36 (d, *J* = 1.8 Hz, 1 H, H-Ar) ppm. **^{13}C NMR** (100 MHz, $CDCl_3$): δ = 14.4 (OCH_2CH_3), 47.5 (NCH_2), 55.3 (OCH_3), 60.5 (OCH_2), 83.6 (C-I), 109.3 (CH-Ar), 114.2 (2 × CH-Ar), 120.4 (C-Ar), 128.5 (2 × CH-Ar), 129.5 (C-Ar), 131.4 (CH-Ar), 140.6 (CH-Ar), 150.3 (C-Ar), 159.1 (C-Ar), 165.4 (CO) ppm. **HRMS** (ESI), *m/z*: $[M + Na]^+$ calcd. for $C_{17}H_{18}INNaO_3^+$: 434.0224; found: 434.0209.

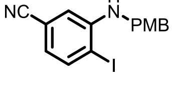

4-Iodo-3-((4-methoxybenzyl)amino)benzonitrile (7g): From **1g** (2.44 g, 10.0 mmol) and 4-methoxybenzaldehyde (1.36 g, 10.0 mmol), compound **7g** (2.46 g, 68%) was obtained according to **GP1b** as a colorless solid, R_f = 0.55 (hexane/EtOAc, 2:1), m.p. 116–117 °C. **^{1}H NMR** (400 MHz, $CDCl_3$): δ = 3.82 (s, 3 H, OCH_3), 4.32 (d, *J* = 5.3 Hz, 2 H, NCH_2), 4.77 (t, *J* = 5.3 Hz, 1 H, NH), 6.67–6.69 (m, 2 H, H-Ar), 6.89–6.93 (m, 2 H, H-Ar), 7.25–7.28 (m, 2 H, H-Ar), 7.75 (d, *J* = 8.5 Hz, 1 H, H-Ar) ppm. **^{13}C NMR** (100 MHz, $CDCl_3$): δ = 47.6 (NCH_2), 55.3 (OCH_3), 90.6 (C-I), 112.6 (CH-Ar), 113.1 (C), 114.3 (2 × CH-Ar), 118.9 (C), 121.3 (CH-Ar), 128.5 (2 × CH-Ar), 129.1 (C-Ar), 139.7 (CH-Ar), 147.5 (C-Ar), 159.2 (C-Ar) ppm. **HRMS** (ESI), *m/z*: $[M + Na]^+$ calcd. for $C_{15}H_{13}IN_2NaO^+$: 386.9965; found: 386.9947.

Synthesis of 2-iodo-5-methoxy-*N*-(4-methoxybenzyl)aniline (**7f**)

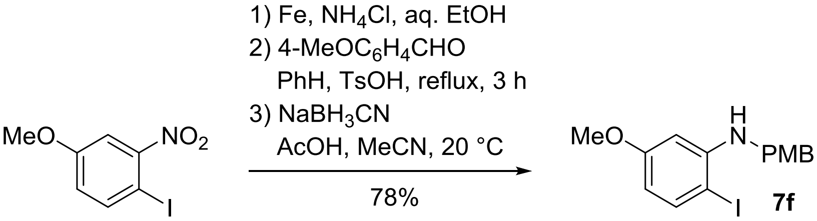

7f

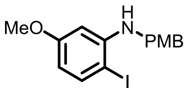

2-Iodo-5-methoxy-*N*-(4-methoxybenzyl)aniline (7f):[12] Iron (1.68 g, 30.0 mmol) was added in one portion to a stirred solution of NH_4Cl (2.67 g, 500 mmol) in H_2O (50 mL), and the resulting suspension was stirred at rt for 5 min. A solution of 1-iodo-4-methoxy-2-nitrobenzene (2.79 g, 10.0 mmol) in EtOH (100 mL) was then added, the resulting mixture was slowly warmed up to 60 °C, and stirring was continued at this temperature for 2 h. The reaction mixture was filtered through a short pad of Celite, and the filtrate was extracted with EtOAc (3 × 50 mL). The combined organic fractions were washed with brine (100 mL), dried over anhydrous Na_2SO_4 and concentrated to give crude 2-iodo-5-methoxyaniline (2.35 g) as a brown oil, which was used in the next step without additional purification.

From this material and 4-methoxybenzaldehyde (1.28 g, 9.44 mmol), compound **7f** (2.89 g, 78% over two steps) was obtained according to **GP1b** as a colorless oil, which spontaneously crystallized when stored in a freezer, R_f = 0.42 (hexane/EtOAc, 4:1), m.p. 55–57 °C. **^{1}H NMR** (400 MHz, $CDCl_3$): δ = 3.72 (s, 3 H, OCH_3), 3.81 (s, 3 H, OCH_3), 4.30 (d, *J* = 5.3 Hz, 2 H, NCH_2), 4.50 (t, *J* = 5.3 Hz, 1 H, NH), 6.10 (dd, *J* = 8.6, 2.8 Hz, 1 H, H-Ar), 6.16 (d, *J* = 2.8 Hz, 1 H, H-Ar), 6.88–6.91 (m, 2 H, H-Ar), 7.27–7.31 (m, 2 H, H-Ar), 7.53 (d, *J* = 8.6 Hz, 1 H, H-Ar) ppm. **^{13}C NMR** (100 MHz, $CDCl_3$): δ = 47.9 (NCH_2), 55.2 (OCH_3), 55.3 (OCH_3), 74.5 (C-I), 98.0 (CH-Ar), 104.1 (CH-Ar), 114.1 (2 × CH-Ar), 128.6 (2 × CH-Ar), 130.4 (C-Ar), 138.9 (CH-Ar), 148.0 (C-Ar), 159.0 (C-Ar), 161.3 (C-Ar) ppm. **HRMS** (ESI), *m/z*: $[M + Na]^+$ calcd. for $C_{15}H_{16}INNaO_2^+$: 392.0118; found: 392.0116. The NMR data agree with the previously published ones.[12]

[12] Zhang, H.; Ben Hay, E.; Geib, S. J.; Curran, D. P. *Beilstein J. Org. Chem.* **2015**, *11*, 1649–1655.

2.4. Synthesis of N-PMB-protected α-substituted methanesulfonamides

General procedures for the sulfonylation of *N*-PMB substituted *o*-iodoanilines (**GP2a**,**b**)

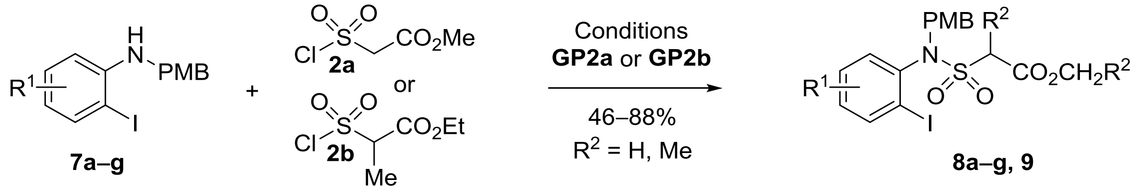

GP2a:

A solution of the corresponding sufonyl chloride **2a** or **2b** (40.0 mmol) in anhydrous MeCN (5 mL) was added in one portion to a stirred solution of the respective amine **7a–c** (20.0 mmol) and pyridine (3.16 g, 40.0 mmol) in anhydrous MeCN (20 mL). The reaction mixture was then warmed up to 60 °C and stirred at this temperature for 48 h. Water (50 mL) was then added, the mixture was acidified with conc. HCl to pH 2 and extracted with CH_2Cl_2 (3 × 75 mL). The combined organic fractions were washed with 5% aq. HCl (2 × 50 mL), brine (50 mL) and dried over anhydrous Na_2SO_4. The solvents were removed, and the crude product was purified by column chromatography (eluent: hexane/EtOAc, gradient from 0 to 50% of EtOAc).

GP2b:

A solution of methyl 2-(chlorosulfonyl)acetate (**2a**) (1.04 g, 6.00 mmol) in CH_2Cl_2 (5 mL) was slowly added to a stirred solution of the respective amine **7d–g** (5.00 mmol) and *N*,*N*-diethylaniline (1.12 g, 7.50 mmol) in CH_2Cl_2 (20 mL), and the mixture was stirred at r.t. for 20 h. The reaction mixture was washed with 5% aq. HCl (2 × 25 mL), brine (30 mL) and dried over anhydrous Na_2SO_4. The solvents were removed, and the crude product was purified by column chromatography (eluent: hexane/EtOAc, gradient from 0 to 100% of EtOAc).

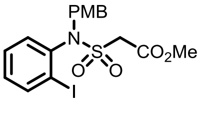

Methyl 2-[*N*-(2-iodophenyl)-*N*-(4-methoxybenzyl)sulfamoyl]acetate (8a): From **2a** (6.90 g, 40.0 mmol) and **7a** (6.78 g, 20.0 mmol), compound **8a** (4.37 g, 46%) was obtained according to **GP2a** as a colorless solid, R_f = 0.23 (hexane/EtOAc, 3:1), m.p. 94–95 °C. **^{1}H NMR** (400 MHz, $CDCl_3$): δ = 3.76 (s, 3 H, OCH_3), 3.82 (s, 3 H, OCH_3), 4.17 (AB system, δ_A = 4.14, δ_B = 4.21, *J* = 14.2 Hz, SO_2CH_2), 4.62 (d, *J* = 14.5 Hz, 1 H, NC*H*H'), 5.01 (d, *J* = 14.5 Hz, 1 H, NCH*H'*), 6.74–6.78 (m, 2 H, H-Ar), 7.00 (td, *J* = 7.7, 1.5 Hz, 1 H, H-Ar), 7.08 (dd, *J* = 7.9, 1.5 Hz, 1 H, H-Ar), 7.08–7.12 (m, 2 H, H-Ar), 7.24 (td, *J* = 7.7, 1.5 Hz, 1 H, H-Ar), 7.90 (dd, *J* =7.9, 1.5 Hz, 1 H, H-Ar) ppm. **^{13}C NMR** (100 MHz, $CDCl_3$): δ = 53.0 (OCH_3), 55.1 (OCH_3), 55.4 (NCH_2), 58.1 (SO_2CH_2), 100.9 (C-I), 113.7 (2 × CH-Ar), 127.0 (C-Ar), 128.9 (CH-Ar), 130.2 (CH-

Ar), 131.2 (2 × CH-Ar), 133.2 (CH-Ar), 140.0 (C-Ar), 140.3 (CH-Ar), 159.4 (C-Ar), 163.5 (CO) ppm. **HRMS** (ESI), *m/z*: $[M + Na]^+$ calcd. for $C_{17}H_{18}INNaO_5S^+$: 497.9843; found: 497.9852.

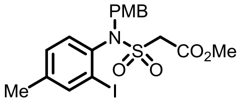

Methyl 2-[*N*-(2-iodo-4-methylphenyl)-*N*-(4-methoxybenzyl) sulfamoyl] acetate (8b): From **2a** (6.90 g, 40.0 mmol) and **7b** (7.06 g, 20.0 mmol), compound **8b** (5.09 g, 52%) was obtained according to **GP2a** as a colorless solid, R_f = 0.26 (hexane/EtOAc, 3:1), m.p. 90–91 °C. **^{1}H NMR** (300 MHz, $CDCl_3$): δ = 2.27 (s, 3 H, CCH_3), 3.76 (s, 3 H, OCH_3), 3.81 (s, 3 H, OCH_3), 4.16 (AB system, δ_A = 4.12, δ_B = 4.19, *J* = 14.2 Hz, SO_2CH_2), 4.59 (d, *J* = 14.5 Hz, 1 H, NC*H*H'), 4.98 (d, *J* = 14.5 Hz, 1 H, NCH*H'*), 6.74–6.79 (m, 2 H, H-Ar), 6.94 (d, *J* = 8.1 Hz, 1 H, H-Ar), 7.03 (dd, *J* = 1.7, 8.1 Hz, 1 H, H-Ar), 7.09–7.14 (m, 2 H, H-Ar), 7.70 (d, *J* = 1.7 Hz, 1 H, H-Ar) ppm. **^{13}C NMR** (75 MHz, $CDCl_3$): δ = 20.5 (CH_3), 53.1 (OCH_3), 55.1 (OCH_3), 55.5 (NCH_2), 58.0 (SO_2CH_2), 100.6 (C-I), 113.7 (2 × CH-Ar), 127.2 (C-Ar), 129.7 (CH-Ar), 131.2 (2 × CH-Ar), 132.7 (CH-Ar), 137.4 (C-Ar), 140.66 (C-Ar), 140.74 (CH-Ar), 159.4 (C-Ar), 163.6 (CO) ppm. **HRMS** (ESI), *m/z*: $[M + Na]^+$ calcd. for $C_{18}H_{20}INNaO_5S^+$: 511.9999; found: 512.0012.

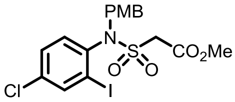

Methyl 2-[*N*-(4-chloro-2-iodophenyl)-*N*-(4-methoxybenzyl) sulfamoyl] acetate (8c): From **2a** (6.90 g, 40.0 mmol) and **7c** (7.47 g, 20.0 mmol), compound **8c** (6.32 g, 62%) was obtained according to **GP2a** as a colorless oil, R_f = 0.20 (hexane/EtOAc, 3:1). **^{1}H NMR** (400 MHz, $CDCl_3$): δ = 3.77 (s, 3 H, OCH_3), 3.83 (s, 3 H, OCH_3), 4.16 (AB system, δ_A = 4.13, δ_B = 4.19, *J* = 14.3 Hz, SO_2CH_2), 4.60 (d, *J* = 14.5 Hz, 1 H, NC*H*H'), 4.98 (d, *J* = 14.5 Hz, 1 H, NCH*H'*), 6.76–6.79 (m, 2 H, H-Ar), 6.99 (d, *J* = 8.5 Hz, 1 H, H-Ar), 7.08–7.12 (m, 2 H, H-Ar), 7.21 (dd, *J* = 2.5, 8.5 Hz, 1 H, H-Ar), 7.87 (d, *J* = 2.5 Hz, 1 H, H-Ar) ppm. **^{13}C NMR** (100 MHz, $CDCl_3$): δ = 53.2 (OCH_3), 55.2 (OCH_3), 55.6 (NCH_2), 58.1 (SO_2CH_2), 101.2 (C-I), 113.8 (2 × CH-Ar), 126.7 (C-Ar), 129.2 (CH-Ar), 131.2 (2 × CH-Ar), 133.8 (CH-Ar), 135.3 (C-Ar), 138.9 (C-Ar), 139.7 (CH-Ar), 159.6 (C-Ar), 163.5 (CO) ppm. **HRMS** (ESI), *m/z*: $[M + Na]^+$ calcd. for $C_{17}H_{17}ClINNaO_5S^+$: 531.9453; found: 531.9457.

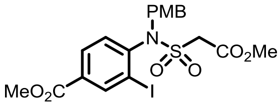

Methyl 3-iodo-(*N*-(4-methoxybenzyl)-*N*-((methoxycarbonylmethyl) sulfonyl)aminobenzoate (8d): From **2a** (1.04 g, 6.00 mmol) and **7d** (1.99 g, 5.00 mmol), compound **8d** (2.05 g, 77%) was obtained according to **GP2a** as a colorless solid, R_f = 0.23 (hexane/EtOAc, 2:1), m.p. 139–140 °C. **^{1}H NMR** (400 MHz, $CDCl_3$): δ = 3.76 (s, 3 H, OCH_3), 3.83 (s, 3 H, OCH_3), 3.90 (s, 3 H, OCH_3), 4.18 (AB system, δ_A = 4.15, δ_B = 4.22, *J* = 14.3 Hz, SO_2CH_2), 4.66 (d, *J* = 14.5 Hz, 1 H, NC*H*H'), 5.02 (d, *J* = 14.4 Hz, 1 H, NCH*H'*), 6.74–6.77 (m, 2 H, H-Ar), 7.07–7.11 (m, 2 H, H-Ar), 7.13 (d, *J* = 8.3 Hz, 1 H, H-Ar), 7.87 (dd, *J* = 8.3, 1.9 Hz, 1 H, H-Ar), 8.52 (d, *J* = 1.9 Hz, 1 H, H-Ar) ppm. **^{13}C NMR** (100 MHz, $CDCl_3$): δ = 52.5 (OCH_3),

53.1 (OCH_3), 55.1 (OCH_3), 55.4 (NCH_2), 58.3 (SO_2CH_2), 100.6 (C-I), 113.8 (2 × CH-Ar), 126.5 (C-Ar), 129.9 (CH-Ar), 131.1 (2 × CH-Ar), 131.5 (C-Ar), 133.2 (CH-Ar), 141.4 (CH-Ar), 144.2 (C-Ar), 159.6 (C-Ar), 163.4 (CO), 164.7 (CO) ppm. **HRMS** (ESI), *m/z*: $[M + Na]^+$ calcd. for $C_{19}H_{20}INNaO_7S^+$: 555.9897; found: 555.9894.

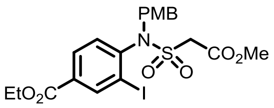

Ethyl 3-iodo-(*N*-(4-methoxybenzyl)-*N*-((methoxycarbonylmethyl) sulfonyl)aminobenzoate (8e): From **2a** (735 mg, 4.38 mmol) and **7e** (1.50 g, 3.65 mmol), compound **8d** (1.66 g, 83%) was obtained according to **GP2a** as a colorless oil, R_f = 0.29 (hexane/EtOAc, 2:1). **^{1}H NMR** (400 MHz, $CDCl_3$): δ = 1.37 (t, *J* = 7.1 Hz, 3 H, CH_2CH_3), 3.75 (s, 3 H, OCH_3), 3.82 (s, 3 H, OCH_3), 4.19 (AB system, δ_A = 4.15, δ_B = 4.22, *J* = 14.3 Hz, SO_2CH_2), 4.35 (q, *J* = 7.1 Hz, 2 H, OCH_2), 4.66 (d, *J* = 14.5 Hz, 1 H, NC*H*H'), 5.00 (d, *J* = 14.5 Hz, 1 H, NCH*H*'), 6.73–6.77 (m, 2 H, H-Ar), 7.07–7.10 (m, 2 H, H-Ar), 7.13 (d, *J* = 8.3 Hz, 1 H, H-Ar), 7.87 (dd, *J* = 8.3, 1.9 Hz, 1 H, H-Ar), 8.51 (d, *J* = 1.9 Hz, 1 H, H-Ar) ppm. **^{13}C NMR** (100 MHz, $CDCl_3$): δ = 14.2 (CH_2CH_3), 53.1 (OCH_3), 55.1 (OCH_3), 55.4 (NCH_2), 58.3 (SO_2CH_2), 61.6 (CH_2CH_3), 100.5 (C-I), 113.8 (2 × CH-Ar), 126.6 (C-Ar), 129.9 (CH-Ar), 131.2 (2 × CH-Ar), 131.9 (C-Ar), 133.1 (CH-Ar), 141.3 (CH-Ar), 144.1 (C-Ar), 159.6 (C-Ar), 163.4 (CO), 164.2 (CO) ppm. **HRMS** (ESI), *m/z*: $[M + Na]^+$ calcd. for $C_{20}H_{22}INNaO_7S^+$: 570.0054; found: 570.0058.

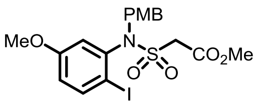

Methyl 2-(*N*-(2-iodo-5-methoxyphenyl)-*N*-(4-methoxybenzyl)sulfamoyl) acetate (8f): From **2a** (1.18 g, 6.84 mmol) and **7f** (2.10 g, 5.69 mmol), compound **8d** (2.46 g, 86%) was obtained according to **GP2a** as a colorless solid, R_f = 0.34 (hexane/EtOAc, 2:1), m.p. 127–128 °C. **^{1}H NMR** (400 MHz, $CDCl_3$): δ = 3.62 (s, 3 H, OCH_3), 3.77 (s, 3 H, OCH_3), 3.82 (s, 3 H, OCH_3), 4.18 (AB system, δ_A 4.15, δ_B 4.21, *J* = 14.2 Hz, SO_2CH_2), 4.59 (d, *J* = 14.5 Hz, 1 H, NC*H*H'), 5.01 (d, *J* = 14.5 Hz, 1 H, NCH*H*'), 6.61–6.64 (m, 2 H, H-Ar), 6.76–6.80 (m, 2 H, H-Ar), 7.11–7.15 (m, 2 H, H-Ar), 7.70 (d, *J* = 8.5 Hz, 1 H, H-Ar) ppm. **^{13}C NMR** (100 MHz, $CDCl_3$): δ = 53.1 (OCH_3), 55.2 (OCH_3), 55.5 (OCH_3), 55.5 (NCH_2), 58.2 (SO_2CH_2), 88.8 (C-I), 113.7 (2 × CH-Ar), 117.1 (CH-Ar), 119.1 (CH-Ar), 127.1 (C-Ar), 131.2 (2 × CH-Ar), 140.2 (CH-Ar), 140.9 (C-Ar), 159.5 (C-Ar), 160.1 (C-Ar), 163.6 (CO) ppm. **HRMS** (ESI), *m/z*: $[M + Na]^+$ calcd. for $C_{18}H_{20}INNaO_6S^+$: 527.9948; found: 527.9950.

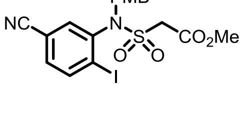

Methyl 2-(*N*-(5-cyano-2-iodophenyl)-*N*-(4-methoxybenzyl)sulfamoyl) acetate (8g): From **2a** (1.05 g, 6.08mmol) and **7g** (1.85 g, 5.08 mmol), compound **8g** (2.23 g, 88%) was obtained according to **GP2a** as a colorless solid, R_f = 0.32 (hexane/EtOAc, 2:1), m.p. 133–134 °C. **^{1}H NMR** (400 MHz, $CDCl_3$): δ = 3.77 (s, 3 H, OCH_3), 3.83 (s, 3 H, OCH_3), 4.17 (AB system, δ_A = 4.15, δ_B = 4.19, *J* = 14.4 Hz, SO_2CH_2), 4.66 (d, *J* = 14.4 Hz, 1 H, NC*H*H'), 4.96 (d, *J* = 14.4 Hz, 1 H, NCH*H*'), 6.76–6.80 (m, 2 H, H-Ar), 7.07–7.10 (m, 2 H, H-Ar),

7.24 (dd, J = 8.2, 1.8 Hz, 1 H, H-Ar), 7.36 (d, J = 1.8 Hz, 1 H, H-Ar), 8.00 (d, J = 8.2 Hz, 1 H, H-Ar) ppm. **^{13}C NMR** (100 MHz, $CDCl_3$): δ = 53.3 (OCH_3), 55.2 (OCH_3), 55.6 (NCH_2), 58.2 (SO_2CH_2), 108.2 (C-I), 113.0 (C), 114.0 (2 × CH-Ar), 117.0 (CN), 126.0 (C), 131.1 (2 × CH-Ar), 132.6 (CH-Ar), 136.0 (CH-Ar), 141.5 (CH-Ar), 141.8 (C-Ar), 159.8 (C-Ar), 163.3 (CO) ppm. **HRMS** (ESI), *m/z*: $[M + Na]^+$ calcd. for $C_{18}H_{17}IN_2NaO_5S^+$: 522.9795; found: 522.9818.

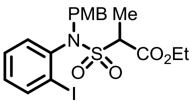

Ethyl 2-[*N*-(2-iodophenyl)-*N*-(4-methoxybenzyl)sulfamoyl]propanoate (9a): From **2b** (8.02 g, 40.0 mmol) and **7a** (6.78 g, 20.0 mmol), compound **9a** (5.64 g, 56%) was obtained according to **GP2a** as a pale yellow oil, R_f = 0.20 (hexane/EtOAc, 3:1). The compound exhibited two sets of signals in its ^{1}H and ^{13}C NMR spectra. *Major diastereomer:* **^{1}H NMR** (300 MHz, $CDCl_3$): δ = 1.31 (t, J = 7.1 Hz, 3 H, CH_2CH_3), 1.79 (d, J = 7.1 Hz, 3 H, $CHCH_3$), 3.75 (s, 3 H, OCH_3), 4.11–4.31 (m, 3 H, C*H*CH_3 + C$H_2$$CH_3$), 4.68 (d, J = 14.2 Hz, 1 H, NC*H*H'), 4.87 (d, J = 14.2 Hz, 1 H, NCH*H'*), 6.71–6.76 (m, 2 H, H-Ar), 6.92–7.26 (m, 5 H, H-Ar), 7.88 (d, J = 7.9 Hz, 1 H, H-Ar) ppm. **^{13}C NMR** (75 MHz, $CDCl_3$): δ = 13.74 (CH_2CH_3), 13.93 ($CHCH_3$), 55.13 (OCH_3), 56.18 (OCH_2), 62.26 (NCH_2), 63.49 (*C*HCH_3), 101.02 (C-I), 113.56 (2 × CH-Ar), 127.12 (C-Ar), 128.82 (CH-Ar), 130.05 (CH-Ar), 131.30 (2 × CH-Ar), 133.71 (CH-Ar), 140.20 (CH-Ar), 140.35 (C-Ar), 159.43 (C-Ar), 166.50 (CO) ppm. *Minor diastereomer:* **^{1}H NMR** (300 MHz, $CDCl_3$): δ = 1.33 (t, J = 7.1 Hz, 3 H, CH_2CH_3), 1.64 (d, J = 7.1 Hz, 3 H, $CHCH_3$), 3.75 (s, 3 H, OCH_3), 4.21–4.31 (m, 3 H, C*H*CH_3 + C$H_2$$CH_3$), 4.59 (d, J = 14.5 Hz, 1 H, NC*H*H'), 5.06 (d, J = 14.5 Hz, 1 H, NCH*H'*), 6.71–6.76 (m, 2 H, H-Ar), 6.92–7.26 (m, 5 H, H-Ar), 7.88 (d, J = 7.9 Hz, 1 H, H-Ar) ppm. **^{13}C NMR** (75 MHz, $CDCl_3$): δ = 13.77 (CH_2CH_3), 13.99 ($CHCH_3$), 55.13 (OCH_3), 55.28 (OCH_2), 62.15 (NCH_2), 64.69 (*C*HCH_3), 100.20 (C-I), 113.56 (2 × CH-Ar), 127.24 (C-Ar), 128.49 (CH-Ar), 129.89 (CH-Ar), 131.22 (2 × CH-Ar), 134.31 (CH-Ar), 140.01 (CH-Ar), 140.35 (C-Ar), 159.34 (C-Ar), 166.89 (CO) ppm. **HRMS** (ESI), *m/z*: $[M + H]^+$ calcd. for $C_{19}H_{23}INO_5S^+$: 504.0336; found: 504.0327.

Synthesis of *N*-(2-iodophenyl)-*N*-(4-methoxybenzyl)-1-phenylmethanesulfonamide (**12**)

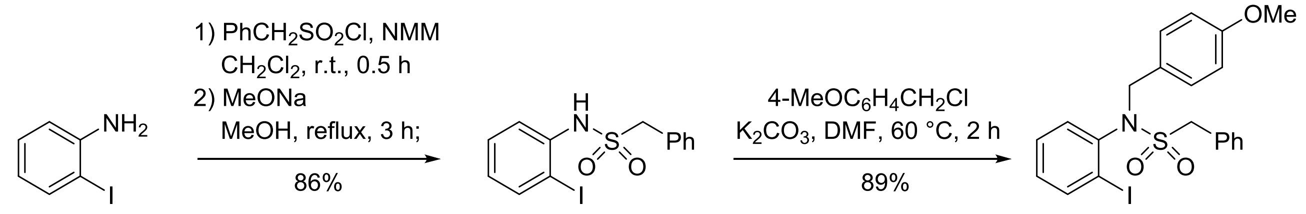

1a **11** **12**

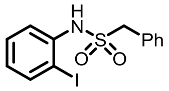

***N*-(2-Iodophenyl)-1-phenylmethanesulfonamide (11)**: A solution of α-toluenesulfonyl chloride (19.1 g, 100 mmol) in CH_2Cl_2 (50 mL) was added in one portion to a stirred solution of 2-iodoaniline (11.0 g, 50.2 mmol) and *N*-methylmorpholine (10.1 g, 100 mmol) in CH_2Cl_2 (100 mL) at rt, and the resulting solution was stirred at rt for 1 h. The reaction mixture was then diluted with CH_2Cl_2 (100 mL), washed with 5% aq. HCl (3 × 50 mL), water (50 mL), dried over anhydrous Na_2SO_4 and concentrated. The residue was dissolved in MeOH (50 mL), MeONa (16.2 g, 300 mmol) was then added in one portion, and the mixture was stirred under reflux for 3 h. The solvents were removed, and the residue was dissolved in a mixture of CH_2Cl_2 (100 mL) and water (50 mL). The mixture was neutralized with 15% aq. HCl, the organic phase was separated, washed with water (2 ×50 mL), brine (30 mL), dried over anhydrous Na_2SO_4 and concentrated on a rotary evaporator. The crude product was purified by recrystallization from hexane/EtOAc to give **11** (16.1 g, 86%) as a colorless solid, m.p. 83–84 °C. **^{1}H NMR** (300 MHz, $CDCl_3$): δ = 4.37 (s, 2 H, CH_2), 6.58 (br. s, 1 H, NH), 6.87 (td, *J* = 1.5, 7.7 Hz, 1 H, H-Ar), 7.20–7.24 (m, 2 H, H-Ar), 7.30–7.36 (m, 4 H, H-Ar), 7.64 (dd, *J* = 1.5, 8.2 Hz, 1 H, H-Ar), 7.79 (dd, *J* = 1.5, 8.0 Hz, 1 H, H-Ar) ppm. **^{13}C NMR** (75 MHz, $CDCl_3$): δ = 58.1 (SO_2CH_2), 90.2 (C-I), 119.4 (CH-Ar), 126.1 (CH-Ar), 127.8 (C-Ar), 128.9 (3 × CH-Ar), 129.8 (CH-Ar), 130.7 (2 × CH-Ar), 137.8 (C-Ar), 139.5 (CH-Ar) ppm. **HRMS** (ESI), *m/z*: $[M + Na]^+$ calcd. for $C_{13}H_{12}INNaO_2S^+$: 395.9526; found: 395.9526.

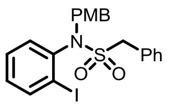

***N*-(2-Iodophenyl)-*N*-(4-methoxybenzyl)-1-phenylmethanesulfonamide (12)**: A solution of 4-methoxybenzyl chloride (3.44 g, 22.0 mmol) in DMF (10 mL) was added dropwise to a mixture of sulfonamide **11** (7.46 g, 20.0 mmol), K_2CO_3 (4.14 g, 30.0 mmol) and DMF (60 mL) at 60 °C within 0.5 h. The eaction mixture was stirred at 60 °C for 2 h, and DMF was then removed on a rotary evaporator. The residue was taken up in CH_2Cl_2 (150 mL) and 15% aq. HCl was carefully added until pH 2 was achieved. The organic phase was then washed with 5% aq. HCl (6 × 50 mL), water (50 mL), brine (30 mL) and dried over Na_2SO_4. The solvents were removed, and the crude product was purified by recrystallization from hexane/EtOAc to give **12** (8.78 g, 89%) as a colorless solid, m.p. 144–146 °C.

^{1}H NMR (300 MHz, $CDCl_3$): δ = 3.78 (s, 3 H, OCH_3), 4.37 (AB system, δ_A = 4.34, δ_B = 4.41, *J* = 13.5 Hz, SO_2CH_2), 4.50 (d, *J* = 14.5 Hz, 1 H, NC*H*H'), 4.96 (d, *J* = 14.5 Hz, 1 H, NCH*H*'), 6.78–6.81 (m, 3 H, H-Ar), 6.98 (td, *J* = 1.6, 7.9 Hz, 1 H, H-Ar), 7.11–7.156 (m, 2 H, H-Ar), 7.18 (td, *J* = 1.4, 7.9 Hz, 1 H, H-Ar), 7.36–7.39 (m, 5 H, H-Ar), 7.91 (dd, *J* = 1.6, 7.9 Hz, 1 H, H-Ar) ppm. **^{13}C NMR** (75 MHz, $CDCl_3$): δ = 54.9 (NCH_2), 55.2 (OCH_3), 60.7 (SO_2CH_2), 101.5 (C-I), 113.7 (2 × CH-Ar), 127.5 (C-Ar), 128.6 (3 × CH-Ar + C-Ar), 128.7 (CH-Ar), 129.9 (CH-Ar), 130.9 (2 × CH-Ar) 131.2 (2 × CH-Ar) 133.2 (CH-Ar), 140.2 (CH-Ar), 141.1 (C-Ar), 159.4 (C-Ar) ppm. **HRMS** (ESI), *m/z*: $[M + Na]^+$ calcd. for $C_{21}H_{20}INNaO_3S^+$: 516.0101; found: 516.0112.

2.5. General procedure for *Pd-catalyzed cyclization of N-alkyl-N-arylsulfonamides (**5**, **8a–g**) (**GP3**)*

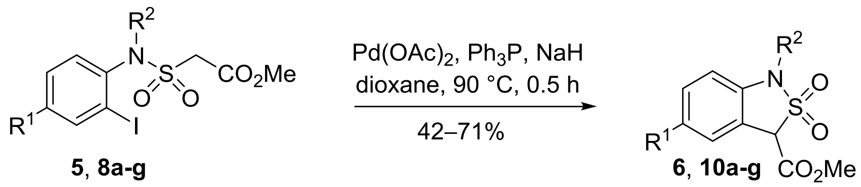

Sodium hydride (60% dispersion in oil, 320 mg, 8.0 mmol) was washed with anhydrous pentane (2 × 5 mL) and was then added to a stirred solution of the respective sulfonamide (2.00 mmol) and PPh_3 (105 mg, 400 µmol) in anhydrous dioxane (10 mL). Argon was bubbled through the resulting mixture for 15 min, and $Pd(OAc)_2$ (23 mg, 100 µmol) was then added in one portion. The mixture was stirred at 90 °C for 30 min, and concentrated down to a volume of 1 mL. The residue was carefully added to a vigorously stirred mixture of 5% aq. HCl (30 mL) and EtOAc (100 mL). The organic phase was separated, and the aqueous layer was extracted with EtOAc (2 × 50 mL). The combined organic fractions were washed with brine (20 mL), dried over anhydrous Na_2SO_4 and concentrated. The crude product was purified by column chromatography (eluent: hexane/EtOAc, gradient from 0 to 100% of EtOAc).

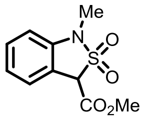

Methyl 1-methyl-1,3-dihydrobenzo[*c*]isothiazole-3-carboxylate 2,2-dioxide (6): From **5** (367 mg, 994 µmol), compound **6** (187 mg, 78%) was obtained according to **GP3** (reaction time was 1 h) as a colorless solid, R_f = 0.20 (hexane/EtOAc, 3:1), m.p. 122–123 °C. ^{1}**H NMR** (300 MHz, $CDCl_3$): δ = 3.17 (s, 3 H, NCH_3), 3.89 (s, 3 H, OCH_3), 5.13 (s, 1 H, SO_2CH), 6.78 (d, J = 8.0 Hz, 1 H, H-Ar), 7.07 (t, J = 7.6 Hz, 1 H, H-Ar), 7.35 (d, J = 7.6 Hz, 1 H, H-Ar), 7.40 (t, J = 8.0 Hz, 1 H, H-Ar) ppm. ^{13}**C NMR** (75 MHz, $CDCl_3$): δ = 27.6 (NCH_3), 53.9 (OCH_3), 64.5 (SO_2CH), 109.7 (CH-Ar), 117.7 (C-Ar), 122.4 (CH-Ar), 126.4 (CH-Ar), 130.6 (CH-Ar), 141.4 (C-Ar), 163.5 (CO) ppm. **HRMS** (ESI), *m/z*: $[M + H]^+$ calcd. for $C_{10}H_{12}NO_4S^+$: 242.0482; found: 242.0484.

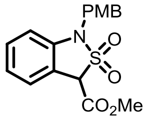

Methyl 1-(4-methoxybenzyl)-1,3-dihydrobenzo[*c*]isothiazole-3-carboxylate 2,2-dioxide (10a): From **8a** (950 mg, 2.00 mmol), compound **10a** (569 mg, 82%) was obtained according to **GP3** as a colorless solid, R_f = 0.28 (hexane/EtOAc, 2:1), m.p. 116–118 °C. ^{1}**H NMR** (400 MHz, $CDCl_3$): δ = 3.79 (s, 3 H, OCH_3), 3.91 (s, 3 H, OCH_3), 4.73 (s, 2 H, NCH_2), 5.18 (s, 1 H, SO_2CH), 6.61 (d, J = 8.0 Hz, 1 H, H-Ar), 6.88–6.90 (m, 2 H, H-Ar), 7.02 (t, J = 7.6 Hz, 1 H, H-Ar), 7.23 (t, J = 8.0 Hz, 1 H, H-Ar), 7.32 (d, J = 7.6 Hz, 1 H, H-Ar), 7.34–7.37 (m, 2 H, H-Ar) ppm. ^{13}**C NMR** (100 MHz, $CDCl_3$): δ = 45.6 (NCH_2), 53.8 (OCH_3), 55.2 (OCH_3), 64.8 (SO_2CH), 110.7 (CH-Ar), 114.2 (2 × CH-Ar), 117.6 (C-Ar), 122.3 (CH-Ar), 126.3 (CH-Ar), 126.7 (C-

Ar), 128.6 (2 × CH-Ar), 130.4 (CH-Ar), 140.6 (C-Ar), 159.3 (C-Ar), 163.6 (CO) ppm. **HRMS** (ESI), *m/z*: $[M + Na]^+$ calcd. for $C_{17}H_{17}NNaO_5S^+$: 370.0720; found: 370.0720.

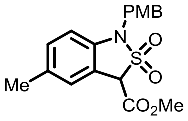

Methyl 1-(4-methoxybenzyl)-5-methyl-1,3-dihydrobenzo[*c*]isothiazole-3-carboxylate 2,2-dioxide (10b): From **8b** (979 mg, 2.00 mmol), compound **10b** (513 mg, 71%) was obtained according to **GP3** as a colorless solid, R_f = 0.26 (hexane/EtOAc, 3:1), m.p. 124–125 °C. **^{1}H NMR** (400 MHz, $CDCl_3$): δ = 2.28 (s, 3H, CCH_3), 3.79 (s, 3 H, O CH_3), 3.90 (s, 3 H, O CH_3), 4.70 (s, 2 H, NCH_2), 5.13 (s, 1 H, SO_2CH), 6.51 (d, *J* = 8.1 Hz, 1 H, H-Ar), 6.86–6.90 (m, 2 H, H-Ar), 7.03 (d, *J* = 8.1 Hz, 1 H, H-Ar), 7.13 (s, 1 H, H-Ar), 7.32–7.36 (m, 2 H, H-Ar) ppm. **^{13}C NMR** (100 MHz, $CDCl_3$): δ = 20.8 (CH_3), 46.1 (NCH_2), 53.8 (OCH_3), 55.3 (OCH_3), 65.0 (SO_2CH), 110.9 (CH-Ar), 114.2 (2 × CH-Ar), 117.8 (C-Ar), 126.7 (CH-Ar), 127.0 (C-Ar), 128.7 (2 × CH-Ar), 130.9 (CH-Ar), 132.2 (C-Ar), 138.4 (C-Ar), 159.3 (C-Ar), 163.8 (CO) ppm. **HRMS** (ESI), *m/z*: $[M + Na]^+$ calcd. for $C_{18}H_{19}NNaO_5S^+$: 384.0876; found: 384.0888.

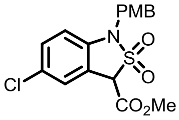

Methyl 5-chloro-1-(4-methoxybenzyl)-1,3-dihydrobenzo[*c*]isothiazole-3-carboxylate 2,2-dioxide (10c): From **8c** (1.02 g, 2.00 mmol), compound **10c** (519 mg, 68%) was obtained according to **GP3** as a colorless solid, R_f = 0.22 (hexane/EtOAc, 3:1), m.p. 117–118 °C. **^{1}H NMR** (400 MHz, $CDCl_3$): δ = 3.79 (s, 3 H, OCH_3), 3.92 (s, 3 H, OCH_3), 4.71 (s, 2 H, NCH_2), 5.16 (s, 1 H, SO_2CH), 6.53 (d, *J* = 8.5 Hz, 1 H, H-Ar), 6.88–6.90 (m, 2 H, H-Ar), 7.20 (dd, *J* = 2.0, 8.5 Hz, 1 H, H-Ar), 7.31–7.34 (m, 3 H, H-Ar) ppm. **^{13}C NMR** (100 MHz, $CDCl_3$): δ = 45.8 (NCH_2), 54.0 (OCH_3), 55.3 (OCH_3), 64.5 (SO_2CH), 111.9 (CH-Ar), 114.3 (2 × CH-Ar), 119.0 (C-Ar), 126.1 (C-Ar), 126.6 (CH-Ar), 127.6 (C-Ar), 128.7 (2 × CH-Ar), 130.4 (CH-Ar), 139.0 (C-Ar), 159.4 (C-Ar), 163.0 (CO) ppm. **HRMS** (ESI), *m/z*: $[M + Na]^+$ calcd. for $C_{17}H_{16}ClNNaO_5S^+$: 404.0330; found: 404.0331.

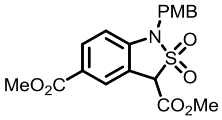

Dimethyl 1-(4-methoxybenzyl)-1,3-dihydrobenzo[c]isothiazole-3,5-dicarboxylate 2,2-dioxide (10d): From **8d** (1.07 g, 2.01 mmol), compound **10d** (634 mg, 78%) was obtained according to **GP3** as a colorless solid, R_f = 0.17 (hexane/EtOAc, 2:1), m.p. 168–169 °C. **^{1}H NMR** (400 MHz, $CDCl_3$): δ = 3.79 (s, 3H, OCH_3), 3.87 (s, 3H, OCH_3), 3.92 (s, 3H, OCH_3), 4.78 (AB system, δ_A = 4.76, δ_B = 4.80, *J* = 16.3 Hz, NCH_2), 5.23 (s, 1H, SO_2CH), 6.63 (d, *J* = 8.5 Hz, 1H, H-Ar), 6.87–6.91 (m, 2 H, H-Ar), 7.31–7.36 (m, 2 H, H-Ar), 7.95 (dd, *J* = 8.5, 1.3 Hz, 1H, H-Ar), 8.00 (br. s, 1H, H-Ar) ppm. **^{13}C NMR** (100 MHz, $CDCl_3$): δ = 45.2 (NCH_2), 52.1 (OCH_3), 54.0 (OCH_3), 55.2 (OCH_3), 64.5 (SO_2CH), 109.8 (CH-Ar), 114.4 (2 × CH-Ar), 117.1 (C-Ar), 124.0 (C-Ar), 125.9 (C-Ar), 127.8 (CH-Ar), 128.6 (2 × CH-Ar), 132.5 (CH-Ar), 143.8 (C-Ar), 159.5 (C-Ar), 163.0 (CO), 165.8 (CO) ppm. **HRMS** (ESI), *m/z*: $[M + Na]^+$ calcd. for $C_{19}H_{19}NNaO_7S$+: 428.0774; found: 428.0775.

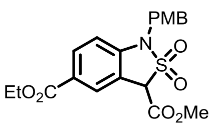

5-Ethyl 1-(4-methoxybenzyl)-3-(methoxycarbonyl)-1,3-dihydrobenzo[c]-isothiazole-5-carboxylate 2,2-dioxide (10e): From **8e** (125 mg, 228 µmol), compound **10e** (82.1 mg, 86%) was obtained according to **GP3** as a colorless solid, R_f = 0.26 (hexane/EtOAc, 2:1), m.p. 116–118 °C. **^{1}H NMR** (400 MHz, $CDCl_3$): δ = 1.34 (t, *J* = 7.1 Hz, 3 H, CH_2CH_3), 3.77 (s, 3 H, OCH_3), 3.90 (s, 3 H, OCH_3), 4.40–4.23 (m, 2 H, CH_2CH_3), 4.76 (AB system, δ_A = 4.73, δ_B = 4.78, *J* = 16.3 Hz, NCH_2), 5.24 (s, 1 H, SO_2CH), 6.62 (d, *J* = 8.5 Hz, 1 H, H-Ar), 6.83–6.91 (m, 2 H, H-Ar), 7.36–7.28 (m, 2 H, H-Ar), 7.94 (dd, *J* = 8.4, 1.4 Hz, 1 H, H-Ar), 8.00 (m, 1 H, H-Ar) ppm. **^{13}C NMR** (100 MHz, $CDCl_3$): δ = 14.2 (CH_2CH_3), 45.1 (NCH_2), 53.9 (OCH_3), 55.2 (OCH_3), 61.0 (CH_2CH_3), 64.5 (SO_2CH), 109.7 (CH-Ar), 114.3 (2 × CH-Ar), 117.0 (C-Ar), 124.3 (C-Ar), 125.8 (C-Ar), 127.7 (CH-Ar), 128.6 (2 × CH-Ar), 132.4 (CH-Ar), 143.7 (C-Ar), 159.4 (C-Ar), 163.0 (CO), 165.3 (CO) ppm. **HRMS** (ESI), *m/z*: $[M + Na]^+$ calcd. for $C_{20}H_{21}NNaO_7S^+$: 442.0931; found: 442.0922.

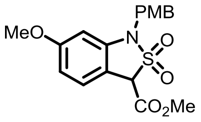

Methyl 6-methoxy-1-(4-methoxybenzyl)-1,3-dihydrobenzo[c]isothiazole-3-carboxylate 2,2-dioxide (10f): From **8f** (1.01 g, 2.00 µmol), compound **10f** (680 mg, 90%) was obtained according to **GP3** (reaction time was 3 h)as a colorless oil, which spontaneously crystallized when stored in a freezer, R_f = 0.27 (hexane/EtOAc, 2:1), m.p. 61–63 °C. **^{1}H NMR** (400 MHz, $CDCl_3$): δ = 3.66 (s, 3 H, OCH_3), 3.76 (s, 3 H, OCH_3), 3.87 (s, 3 H, OCH_3), 4.69 (AB system, δ_A = 4.67, δ_B = 4.70, *J* = 16.3 Hz, NCH_2), 5.14 (s, 1 H, SO_2CH), 6.17 (d, *J* = 2.4 Hz, 1 H, H-Ar), 6.52 (dd, *J* = 8.5, 2.4 Hz, 1 H, H-Ar), 6.85–6.90 (m, 2 H, H-Ar), 7.20 (dd, *J* = 8.5, 0.8 Hz, 1 H, H-Ar), 7.32–7.38 (m, 2 H, H-Ar) ppm. **^{13}C NMR** (100 MHz, $CDCl_3$): δ = 45.4 (NCH_2), 53.6 (OCH_3), 55.1 (OCH_3), 55.2 (OCH_3), 64.5 (SO_2CH), 97.8 (CH-Ar), 106.8 (CH-Ar), 109.2 (C-Ar), 114.1 (2 × CH-Ar), 126.6 (C-Ar), 127.1 (CH-Ar), 128.6 (2 × CH-Ar), 141.5 (C-Ar), 159.2 (C-Ar), 161.3 (C-Ar), 163.8 (CO) ppm. **HRMS** (ESI), *m/z*: $[M + Na]^+$ calcd. for $C_{18}H_{19}NNaO_6S^+$: 400.0825; found: 400.0812.

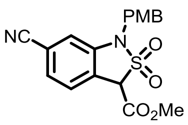

Methyl 6-cyano-1-(4-methoxybenzyl)-1,3-dihydrobenzo[c]isothiazole-3-carboxylate 2,2-dioxide (10g): From **8g** (1.00 g, 2.00 µmol), compound **10g** (693 mg, 93%) was obtained according to **GP3** (reaction time was 5 min) as a colorless solid, R_f = 0.30 (hexane/EtOAc, 1:2), m.p. 146–147 °C. **^{1}H NMR** (400 MHz, $CDCl_3$): δ = 3.81 (s, 3 H, OCH_3), 3.93 (s, 3 H, OCH_3), 4.75 (s, 2 H, NCH_2), 5.23 (s, 1 H, SO_2CH), 6.80 (d, *J* = 1.2 Hz, 1 H, H-Ar), 6.86–6.98 (m, 2 H, H-Ar), 7.32 (dd, *J* = 7.9, 1.2 Hz, 1 H, H-Ar), 7.31–7.35 (m, 2 H, H-Ar), 7.46 (d, *J* = 7.9 Hz, 1 H, H-Ar) ppm. **^{13}C NMR** (100 MHz, $CDCl_3$): δ = 45.4 (NCH_2), 54.1 (OCH_3), 55.2 (OCH_3), 64.3 (SO_2CH), 112.9 (CH-Ar), 114.1 (C), 114.5 (2 × CH-Ar), 117.8 (C), 122.1 (C),

125.2 (C-Ar), 126.0 (CH-Ar), 127.3 (CH-Ar), 128.6 (2 × CH-Ar), 140.9 (C-Ar), 159.6 (C-Ar), 162.4 (CO) ppm. **HRMS** (ESI), *m/z*: $[M + Na]^+$ calcd. for $C_{18}H_{16}N_2NaO_5S^+$: 395.6072; found: 395.0655.

*2.6. Synthesis of methyl 3-(4-methoxyphenyl)-1H-3,4-dihydrobenzo[d][1,2]thiazine-1-carboxylate 2,2-dioxide (**21**)*

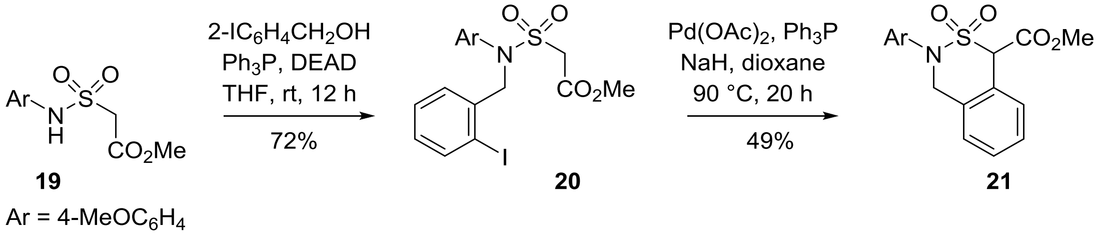

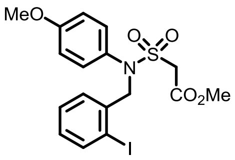

Methyl 2-[*N*-(2-iodobenzyl)-*N*-(4-methoxyphenyl)sulfamoyl]acetate (20): Diethyl azodicarboxylate (DEAD) (1.31 g, 7.52 mmol) was slowly added to a stirred solution of the sulfonamide **19** (1.30 g, 5.01 mmol), (2-iodophenyl)methanol (1.76 g, 7.52 mmol) and Ph_3P (1.97 g, 7.51 mmol) in anhydrous THF (10 mL) at 0 °C, and the resulting mixture was stirred at rt for 12 h. The solvents were then removed, and the product was isolated by column chromatography (hexane/EtOAc 6:1, R_f = 0.58) to give the sulfonamide **20** (1.95 g, 82%) as a colorless solid, m.p. 89–91 °C.

^{1}H NMR (400 MHz, $CDCl_3$): δ = 3.76 (s, 3 H, OCH_3), 3.89 (s, 3 H, OCH_3), 4.04 (s, 2 H, SO_2CH_2), 4.99 (s, 2 H, NCH_2), 6.81–6.85 (m, 2 H, H-Ar), 6.90 (td, *J* = 7.7, 1.4 Hz, 1 H, H-Ar), 7.31 (td, *J* = 7.7, 0.9 Hz, 1 H, H-Ar), 7.35–7.39 (m, 2 H, H-Ar), 7.57 (dd, *J* = 7.8, 1.4 Hz, 1 H, H-Ar), 7.70 (dd, *J* = 7.8, 0.9 Hz, 1 H, H-Ar) ppm. **^{13}C NMR** (100 MHz, $CDCl_3$): δ = 53.2 (OCH_3), 53.9 (CH_2), 55.4 (OCH_3), 60.9 (CH_2), 98.7 (C-I), 114.6 (2 × CH-Ar), 128.4 (CH-Ar), 129.3 (CH-Ar), 129.7 (CH-Ar), 130.4 (C-Ar), 130.5 (2 × CH-Ar), 138.4 (C-Ar), 139.3 (CH-Ar), 159.5 (C-Ar), 163.9 (CO) ppm.
HRMS (ESI), *m/z*: $[M + Na]^+$ calcd. for $C_{17}H_{18}INNaO_5S^+$: 497.9843; found: 497.9842.

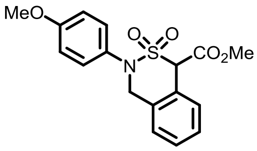

Methyl 3-(4-methoxyphenyl)-1*H*-3,4-dihydrobenzo[*d*][1,2]thiazine-1-carboxylate 2,2-dioxide (21): From **20** (310 mg, 652 µmol), compound **21** (111 mg, 49%) was obtained according to **GP3** (reaction time was 20 h) as a colorless solid, R_f = 0.10 (hexane/EtOAc, 6 : 1), m.p. 105–106 °C. **^{1}H NMR** (400 MHz, $CDCl_3$): δ = 3.78 (s, 3 H, OCH_3), 3.79 (s, 3 H, OCH_3), 5.03 (AB system, δ_A 4.97, δ_B 5.08, *J* = 16.6 Hz, NCH_2), 5.23 (s, 1 H, SO_2CH), 6.84–6.88 (m, 2 H, H-Ar), 7.15 (d, *J* = 7.6 Hz, 1 H, H-Ar), 7.22 (d, *J* = 8.4 Hz, 1 H, H-Ar), 7.24–7.28 (m, 2 H, H-Ar), 7.34 (td, *J* = 7.6, 1.3 Hz, 1 H, H-Ar), 7.40 (td, *J* = 7.6, 1.3 Hz, 1 H, H-Ar) ppm. **^{13}C NMR** (125 MHz, $CDCl_3$): δ = 53.4 (OCH_3), 55.4 (OCH_3), 56.0 (NCH_2), 64.8 (SO_2CH), 114.4 (2 × CH-Ar), 126.6 (CH-Ar), 127.8 (CH-Ar), 128.0 (2 × CH-Ar), 128.1 (C-Ar), 129.1 (CH-Ar), 129.9 (CH-Ar), 130.8 (C-Ar), 132.6 (C-Ar), 159.0 (C-Ar), 165.5 (CO) ppm. **HRMS** (ESI), *m/z*: $[M + Na]^+$ calcd. for $C_{17}H_{17}NNaO_5S^+$: 370.0720; found: 370.0701.

2.7. Miscellaneous transformations

Synthesis of the indole 2-(4-methoxyphenyl)-3-phenyl-*1H*-indole (**18**)

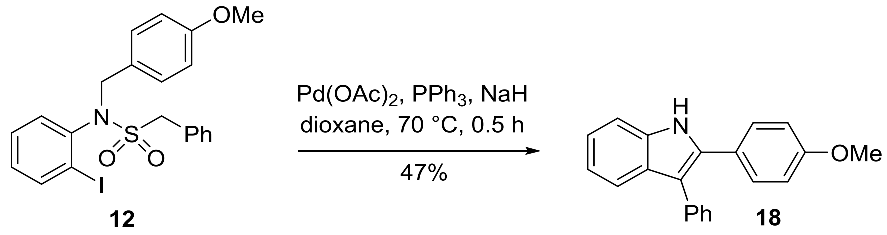

2-(4-Methoxyphenyl)-3-phenyl-1*H*-indole (18): From **12** (494 mg, 1.00 mmol) compound **18** (139 mg, 46%) was obtained according to **GP3** (reaction was carried out at 70 °C) as a yellowish solid m.p. 92–93 °C, lit.[13] m.p. 94–95 °C. **^{1}H NMR** (300 MHz, $CDCl_3$): δ = 3.82 (s, 3 H, OCH_3), 6.85–6.89 (m, 2 H, H-Ar), 7.12–7.46 (m, 10 H, H-Ar), 7.68 (d, *J* = 8.0 Hz, 1 H, H-Ar), 8.18 (br s, 1 H, NH) ppm. **HRMS** (ESI), *m/z*: $[M]^+$ calcd. for $C_{21}H_{17}NO^+$: 299.1305; found: 299.1300.

Synthesis of the methyl 1-(4-methoxybenzyl)-3-(methoxycarbonyl)-1,3-dihydrobenzo[*c*]isothiazole-5-carboxylic acid 2,2-dioxide (**10h**)

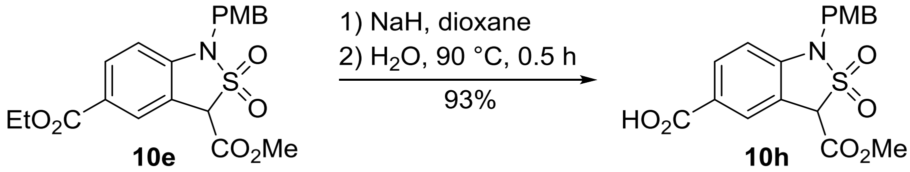

1-(4-Methoxybenzyl)-3-(methoxycarbonyl)-1,3-dihydrobenzo[c]isothiazole-5-carboxylic acid 2,2-dioxide (10h): Sodium hydride (60% dispersion in oil, 80.1 mg, 2.00 mmol) was washed with anhydrous pentane (2 × 1 mL) and was then added to a stirred solution of the sultam **10e** (210 mg, 501 µmol) in dioxane (3 mL). The resulting mixture was stirred at rt for 5 min, and H_2O (36.0 µL, 2.00 mmol) was carefully added. The reaction mixture was warmed up to 90 °C, stirred at this temperature for 30 min, and concentrated down to a volume of 1 mL. The residue was added to a vigorously stirred mixture of 5% aq. HCl (10 mL) and EtOAc (20 mL). The organic phase was separated, and the aqueous layer was extracted with EtOAc (2 × 10 mL). The combined organic fractions were dried over anhydrous Na_2SO_4 and concentrated to give the acid **10h** (183 mg, 93%) as a colorless solid, R_f = 0.31 (EtOAc), m.p. 210–215 °C (dec.). **^{1}H NMR** (400 MHz, DMSO-d_6): δ = 3.73 (s, 3 H, OCH_3), 3.84 (s, 3 H, OCH_3), 4.84 (s, 2 H, NCH_2), 6.12 (s, 1 H, SO_2CH), 6.88 (d, *J* = 8.4 Hz, 1 H, H-Ar), 6.91–6.95 (m, 2 H, H-Ar), 7.34–7.37 (m, 2 H, H-Ar), 7.90 (dd, *J* = 8.4, 1.8 Hz, 1 H, H-Ar), 8.00 (s, 1 H, H-Ar), 12.86 (br. s, 1 H, CO_2H) ppm. **^{13}C NMR** (100 MHz, DMSO-d_6): δ = 43.7 (NCH_2), 53.7 (OCH_3), 55.1 (OCH_3), 64.1 (SO_2CH), 109.6 (CH-Ar), 114.1 (2 × CH-Ar), 117.4 (C-Ar), 124.1 (C-Ar), 126.6 (C-Ar), 128.3 (CH-Ar), 128.8 (2 × CH-Ar), 131.7 (CH-Ar),

[13] Cacchi, S.; Fabrizi, G.; Goggiamani, A. *Adv. Synth. Catal.* **2006**, *348*, 1301–1305.

143.0 (C-Ar), 158.9 (C-Ar), 163.2 (CO), 166.5 (CO) ppm. **HRMS** (ESI), *m/z*: $[M + Na]^+$ calcd. for $C_{18}H_{17}NNaO_7S^+$ 414.0608; found: 414.0618.

Synthesis of the methyl 1-(4-methoxybenzyl)-3-(nitrooxy)-1,3-dihydrobenzo[*c*]isothiazole-3-carboxylate 2,2-dioxide (**22**)

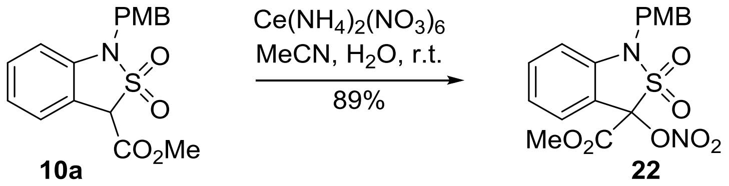

Methyl 1-(4-methoxybenzyl)-3-(nitrooxy)-1,3-dihydrobenzo[c]isothiazole-3-carboxylate-2,2-dioxide (22): A solution of $Ce(NH_4)_2(NO_3)_6$ (3.30 g, 6.02 mmol) in water (20 mL) was slowly added to a stirred solution of the sultam **10a** (696 mg, 2.00 mmol) in CH_3CN (30 mL) at 0 °C, and the resulting mixture was stirred at r.t. for 1 h. Acetonitrile was removed on a rotary evaporator, and the residue was extracted with EtOAc (3 × 20 mL). The combined organic fractions were dried over anhydrous Na_2SO_4 and concentrated. The crude product was subjected to column chromatography (eluent: hexane/EtOAc, gradient from 0 to 50% of EtOAc) to give **22** (726 mg, 89%) as a pale orange solid, R_f = 0.28 (hexane/EtOAc, 2:1), m.p. 110–115 °C (dec.). **^{1}H NMR** (400 MHz, $CDCl_3$): δ = 3.80 (s, 3 H, OCH_3), 3.90 (s, 3 H, OCH_3), 4.82 (AB system, δ_A = 4.75, δ_B = 4.88, *J* = 16.2 Hz, NCH_2), 6.66 (d, *J* = 8.0 Hz, 1 H, H-Ar), 6.89–6.93 (m, 2 H, H-Ar), 7.04 (t, *J* = 7.6 Hz, 1H, H-Ar), 7.27–7.48 (m, 4 H, H-Ar) ppm. **^{13}C NMR** (100 MHz, $CDCl_3$): δ = 46.8 (NCH_2), 54.6 (OCH_3), 55.3 (OCH_3), 92.6 (SO_2CO), 111.8 (CH-Ar), 114.3 (2 × CH-Ar), 116.6 (C-Ar), 122.9 (CH-Ar), 125.8 (CH-Ar), 126.4 (C-Ar), 128.8 (2 × CH-Ar), 132.9 (CH-Ar), 139.8 (C-Ar), 159.5 (C-Ar), 162.5 (CO) ppm. **HRMS** (ESI), *m/z*: $[M + Na]^+$ calcd. for $C_{17}H_{16}N_2NaO_8S^+$: 431.0520; found: 431.0525.

Synthesis of the methyl 1,3-dihydrobenzo[c]isothiazole-3-carboxylate-2,2-dioxide (**23**)

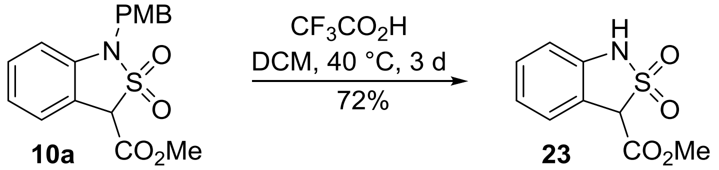

Methyl 1,3-dihydrobenzo[c]isothiazole-3-carboxylate-2,2-dioxide (23): A solution of CF_3CO_2H (570 mg, 5.00 mmol) in CH_2Cl_2 (1 mL) was added in one portion to a stirred solution of the sultam **10a** (347 mg, 1.00 mmol) in CH_2Cl_2 (30 mL), and the resulting mixture was stirred under reflux for 3 days. The solvent was evaporated, and the crude product was purified by column chromatography (eluent: hexane/EtOAc, gradient from 30 to 80% of EtOAc) to give sultam **23** (163 mg, 72%) as a

colorless oil, R_f = 0.39 (hexane/EtOAc, 1:1). **^{1}H NMR** (400 MHz, $CDCl_3$): δ = 3.87 (s, 3 H, OCH_3), 5.15 (s, 1 H, SO_2CH), 6.91 (br. s, 1 H, NH), 6.96 (d, *J* = 8.1 Hz, 1 H, H-Ar), 7.14 (t, *J* = 7.5 Hz, 1 H, H-Ar), 7.31–7.35 (m, 2 H, H-Ar) ppm. **^{13}C NMR** (100 MHz, $CDCl_3$): δ = 53.8 (OCH_3), 66.0 (SO_2CH), 114.8 (CH-Ar), 121.3 (C-Ar), 124.3 (CH-Ar), 126.5 (CH-Ar), 130.5 (CH-Ar), 138.6 (C-Ar), 163.9 (CO) ppm. **HRMS** (ESI), *m/z*: $[M + Na]^+$ calcd. for $C_9H_9NNaO_4S^+$: 250.0144; found: 250.0137.

3 NMR spectra

3.1. ^{1}H and ^{13}C NMR spectra of N-PMB substituted o-iodoanilines

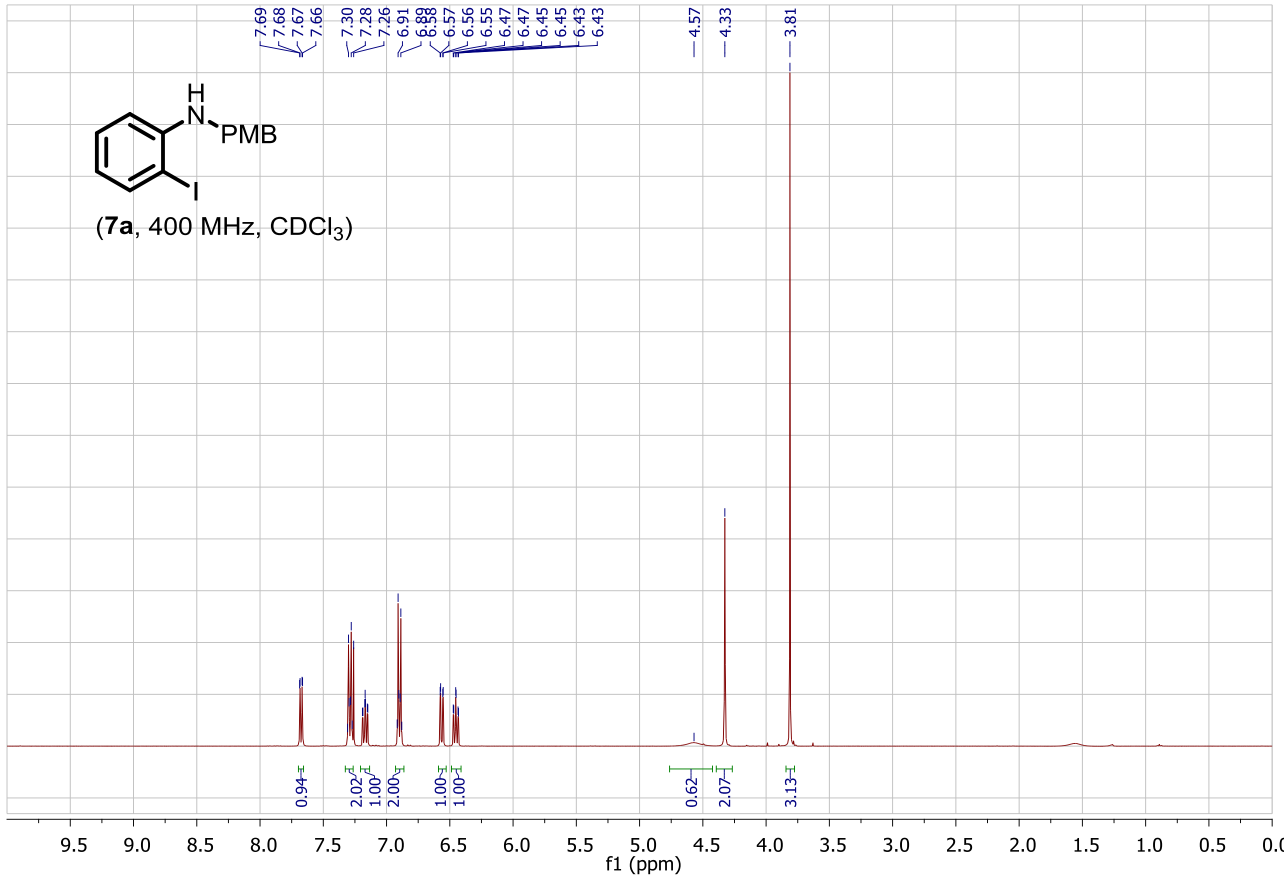

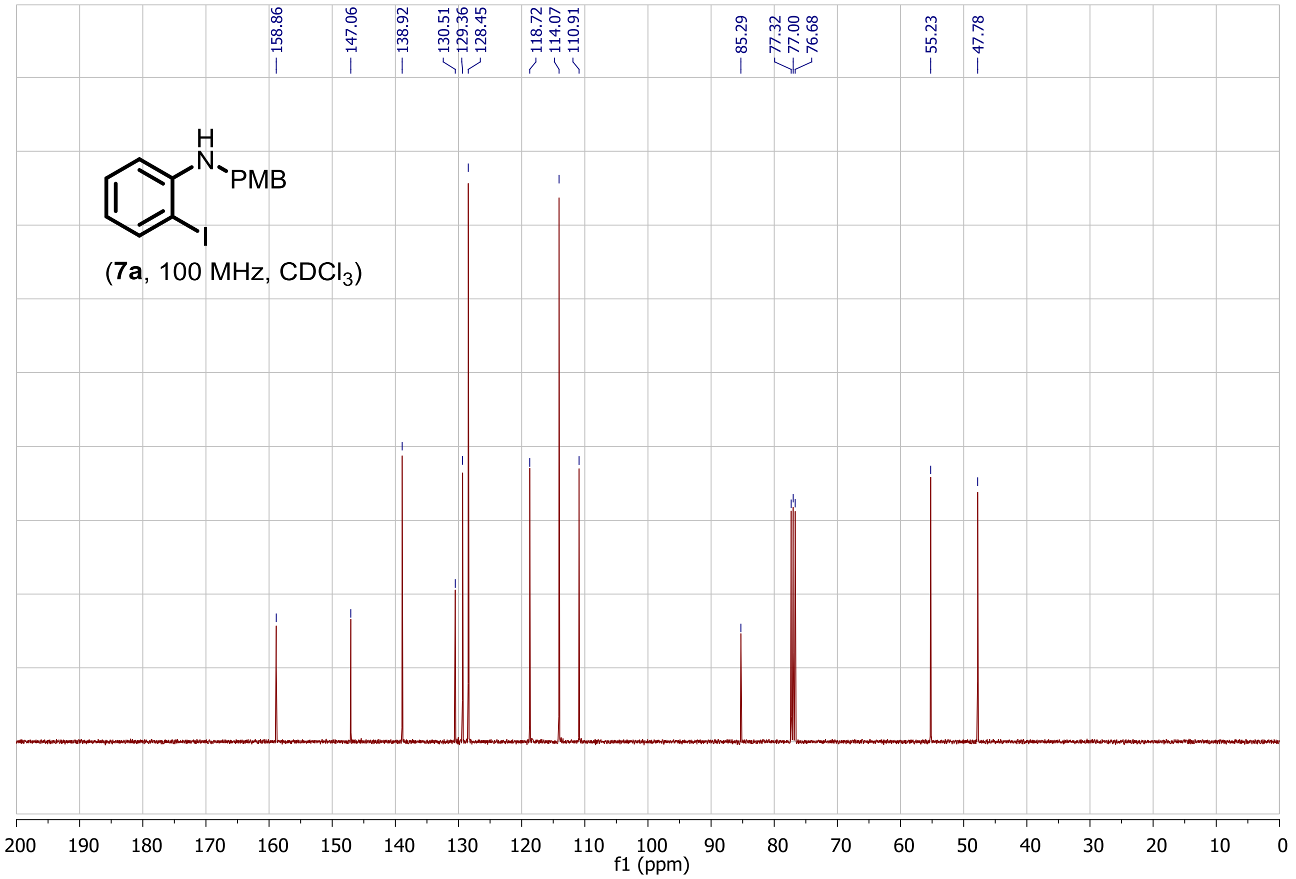

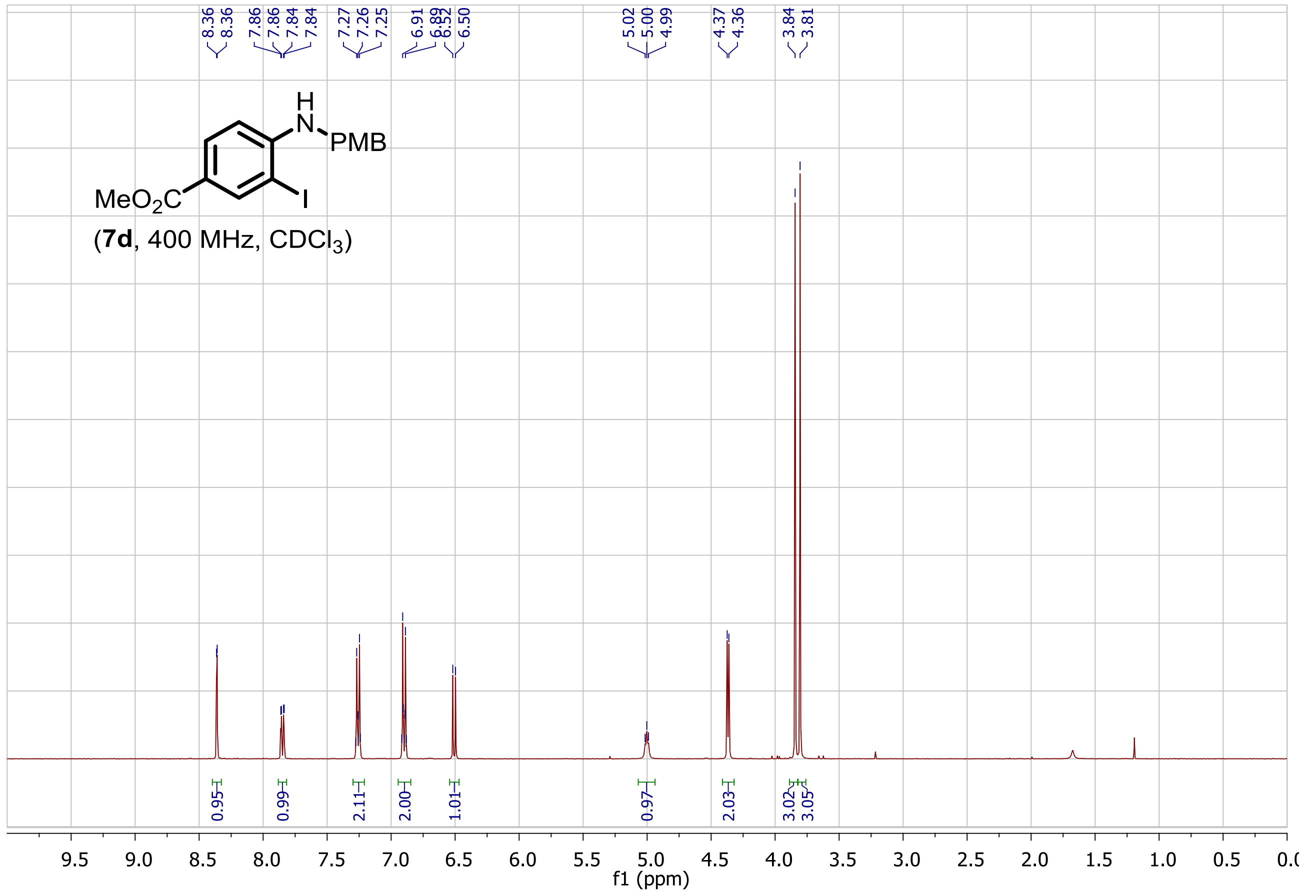
8.36
8.36
7.86
7.86
7.84
7.84
7.27
7.26
7.25
6.91
6.89
6.52
6.50
5.02
5.00
4.99
4.37
4.36
3.84
3.81
H
N
PMB
MeO2C
I
(7d, 400 MHz, CDCl3)
0.95
0.99
2.11
2.00
1.01
0.97
2.03
3.02
3.05
f1 (ppm)

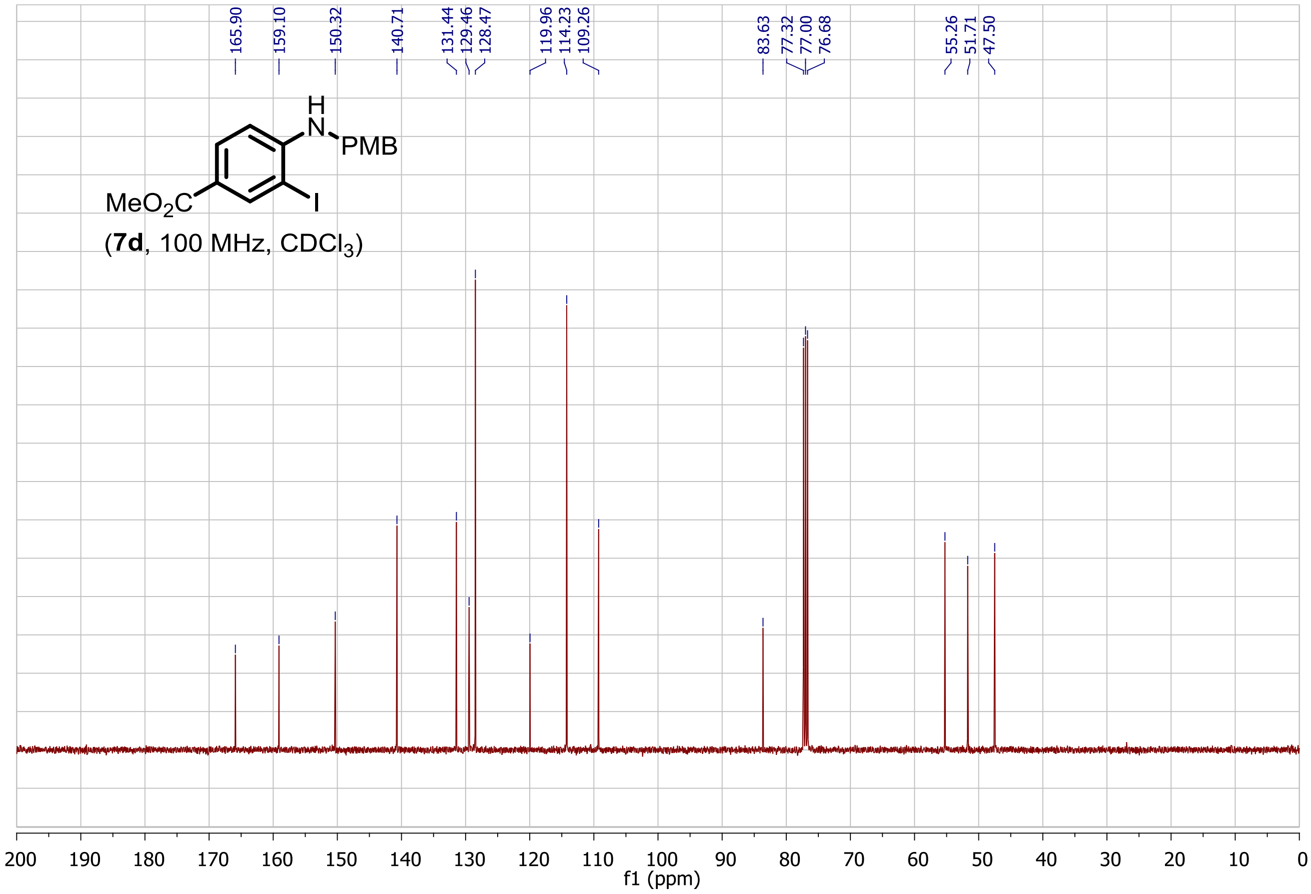
165.90
159.10
150.32
140.71
131.44
129.46
128.47
119.96
114.23
109.26
83.63
77.32
77.00
76.68
55.26
51.71
47.50
H
N
PMB
MeO2C
I
(7d, 100 MHz, CDCl3)
f1 (ppm)

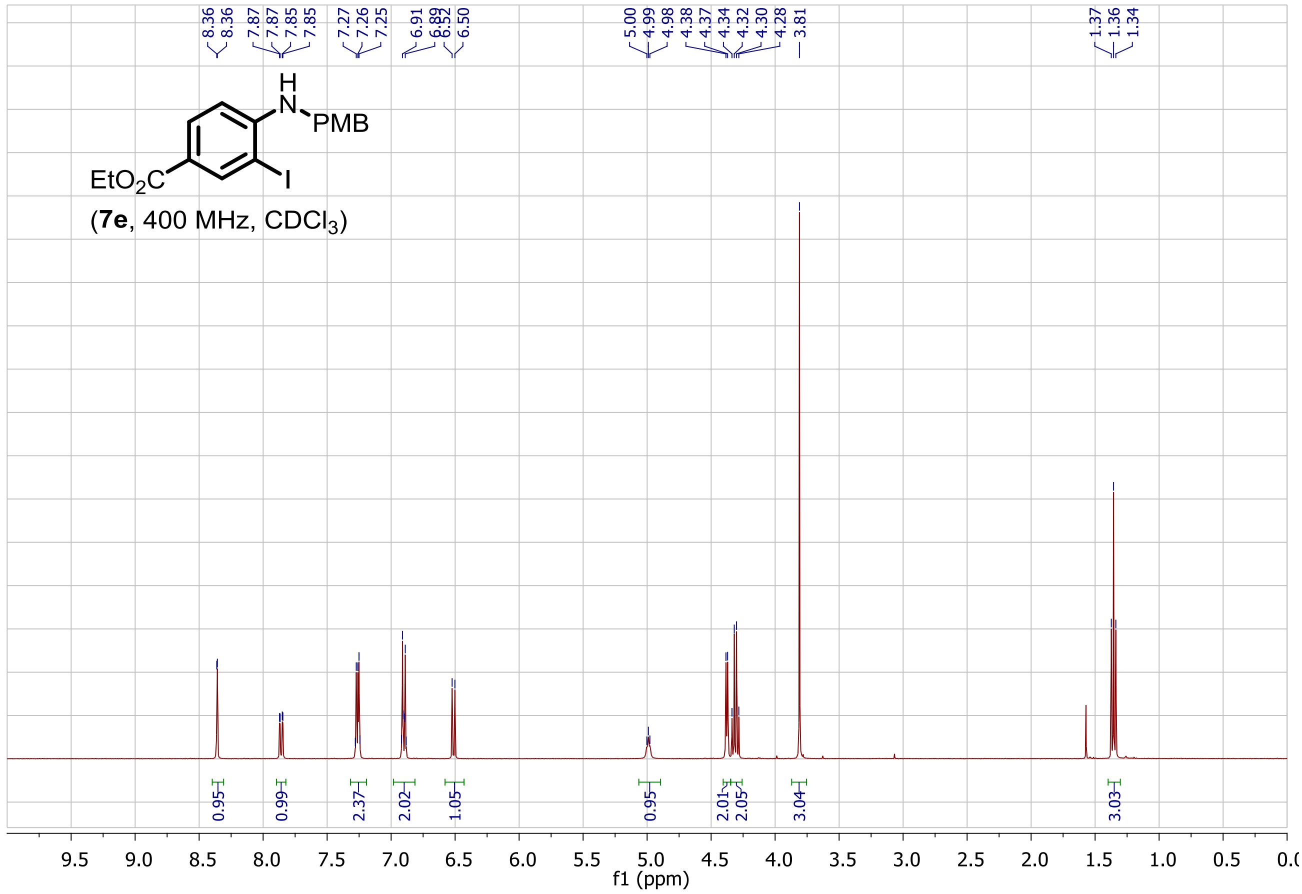

8.36
8.36
7.87
7.87
7.85
7.85
7.27
7.26
7.25
6.91
6.89
6.52
6.50
5.00
4.99
4.98
4.38
4.37
4.34
4.32
4.30
4.28
3.81
1.37
1.36
1.34
H
N
PMB
EtO2C
I
(7e, 400 MHz, CDCl3)
0.95
0.99
2.37
2.02
1.05
0.95
2.01
2.05
3.04
3.03
f1 (ppm)

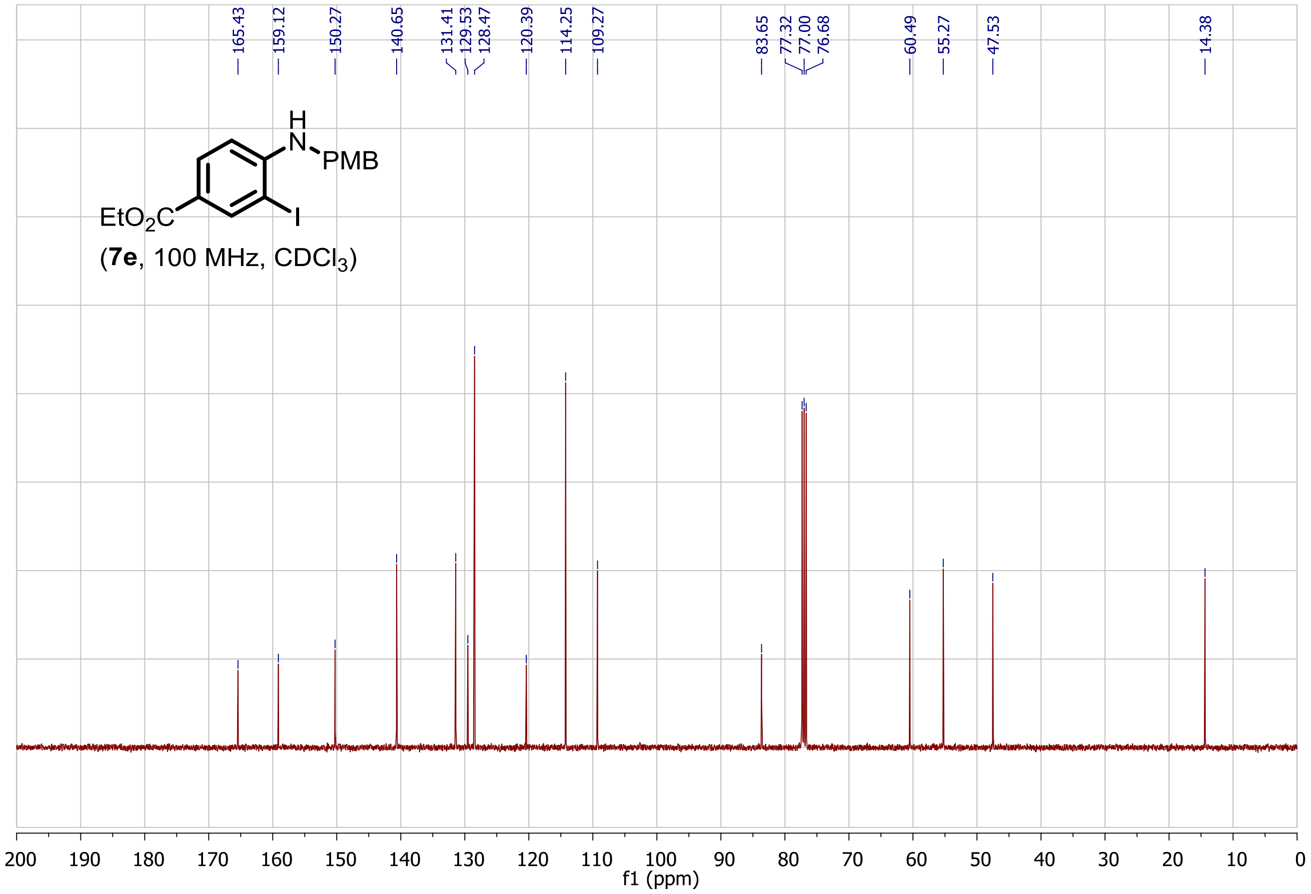

165.43
159.12
150.27
140.65
131.41
129.53
128.47
120.39
114.25
109.27
83.65
77.32
77.00
76.68
60.49
55.27
47.53
14.38
H
N
PMB
EtO2C
I
(7e, 100 MHz, CDCl3)
f1 (ppm)

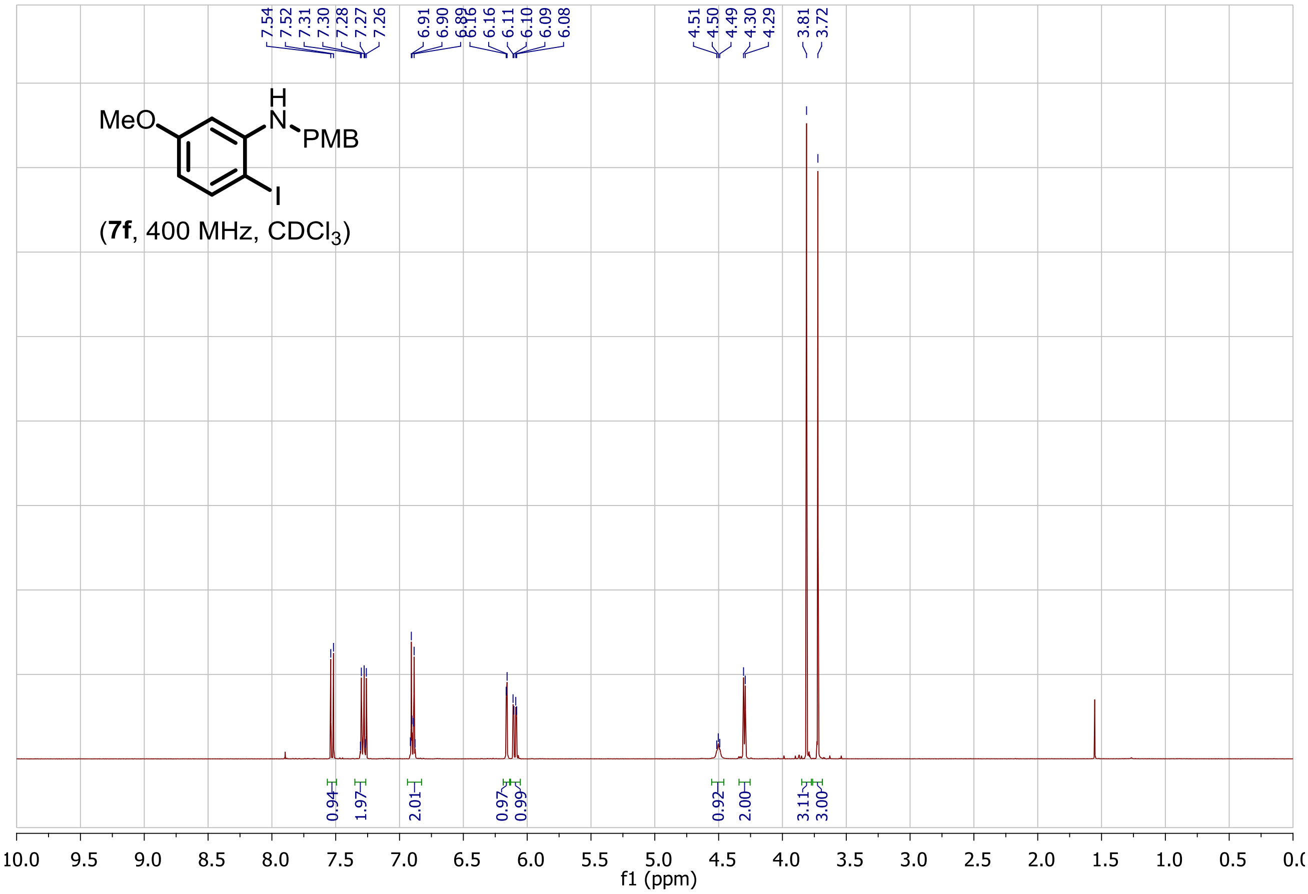
7.54
7.52
7.31
7.30
7.28
7.27
7.26
6.91
6.90
6.89
6.16
6.11
6.10
6.09
6.08
4.51
4.50
4.49
4.30
4.29
3.81
3.72
MeO
H
N
PMB
I
(**7f**, 400 MHz, $CDCl_3$)
0.94
1.97
2.01
0.97
0.99
0.92
2.00
3.11
3.00
f1 (ppm)

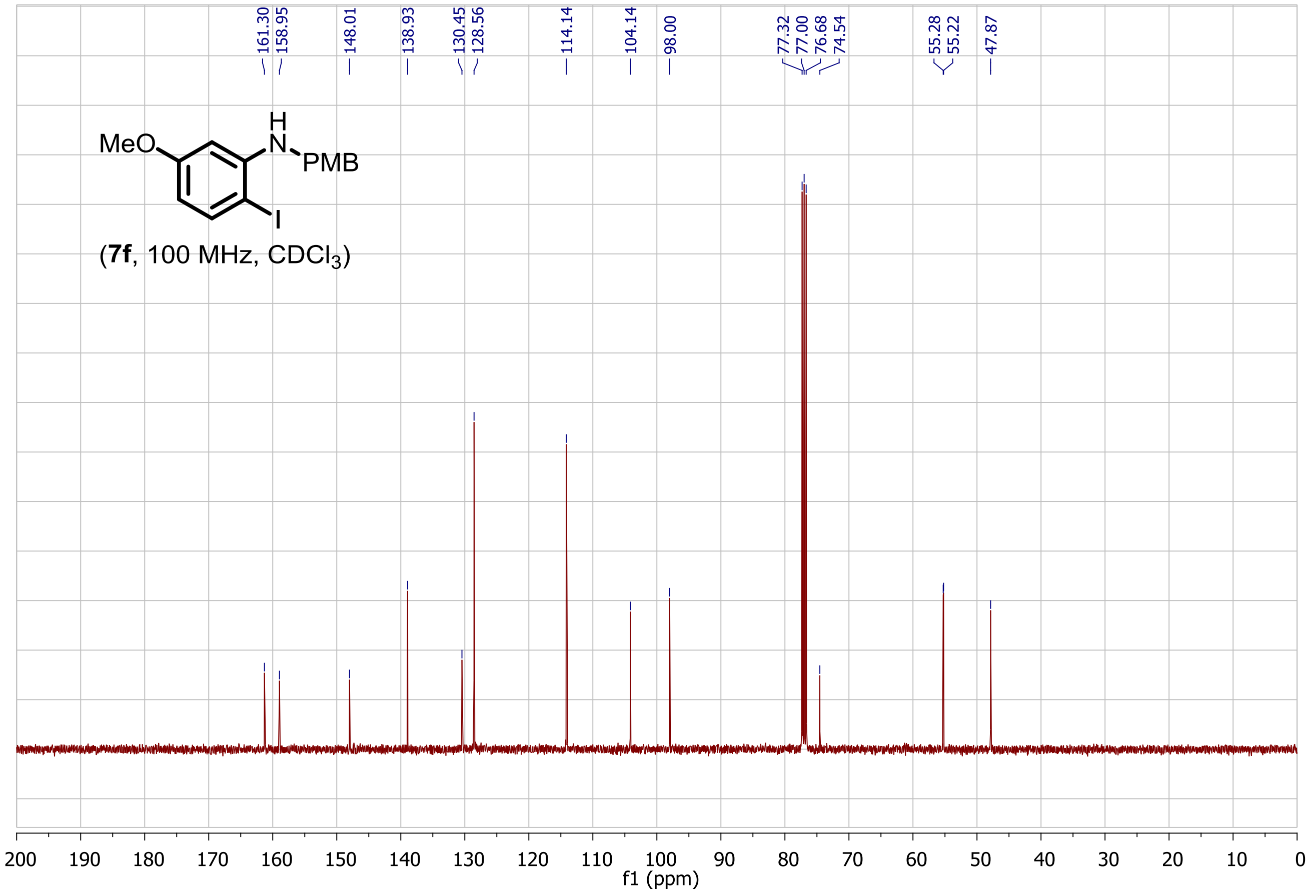
161.30
158.95
148.01
138.93
130.45
128.56
114.14
104.14
98.00
77.32
77.00
76.68
74.54
55.28
55.22
47.87
MeO
H
N
PMB
I
(**7f**, 100 MHz, $CDCl_3$)
f1 (ppm)

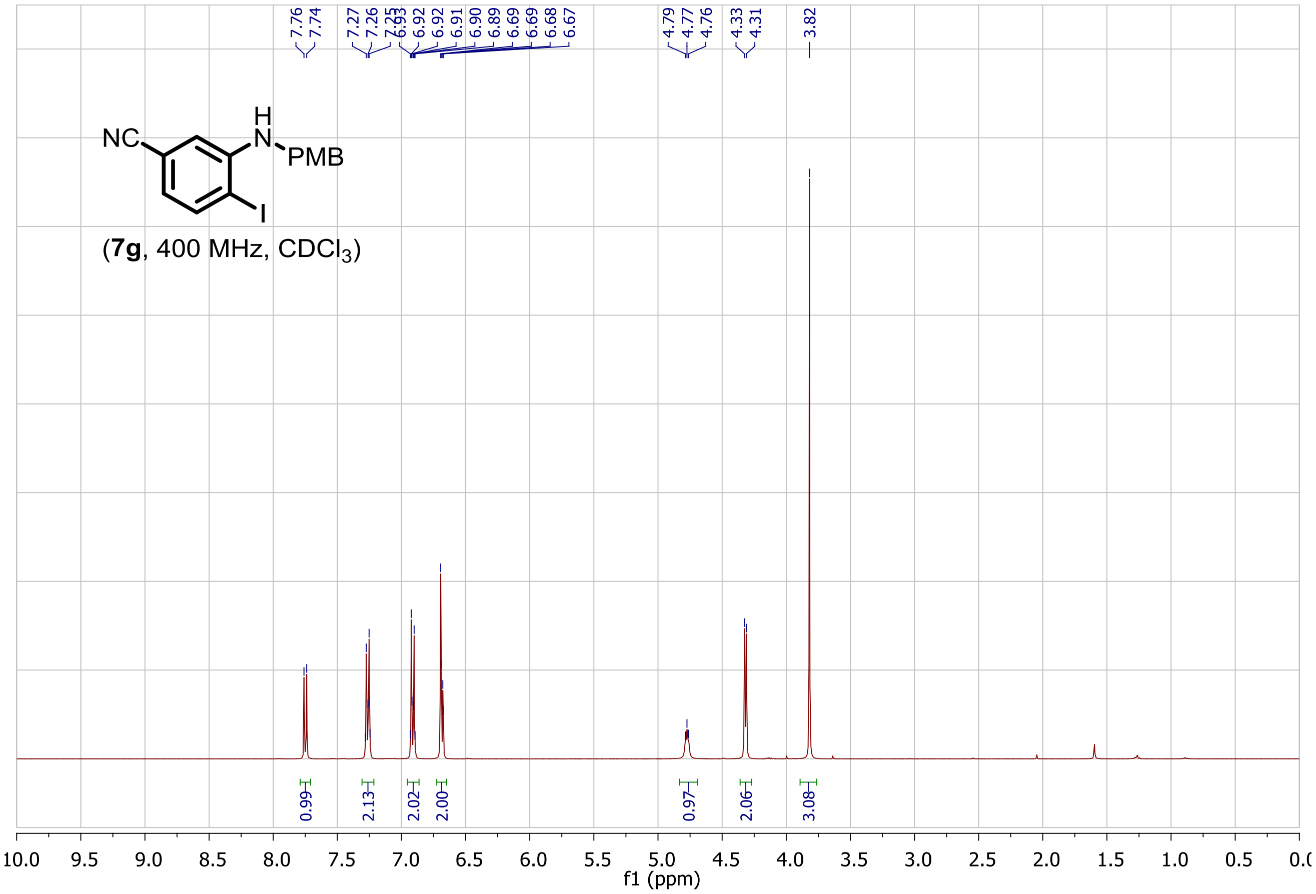
NC
H
N
PMB
I
(7g, 400 MHz, CDCl3)
7.76
7.74
7.27
7.26
7.25
6.93
6.92
6.92
6.91
6.90
6.89
6.69
6.69
6.68
6.67
4.79
4.77
4.76
4.33
4.31
3.82
0.99
2.13
2.02
2.00
0.97
2.06
3.08
f1 (ppm)

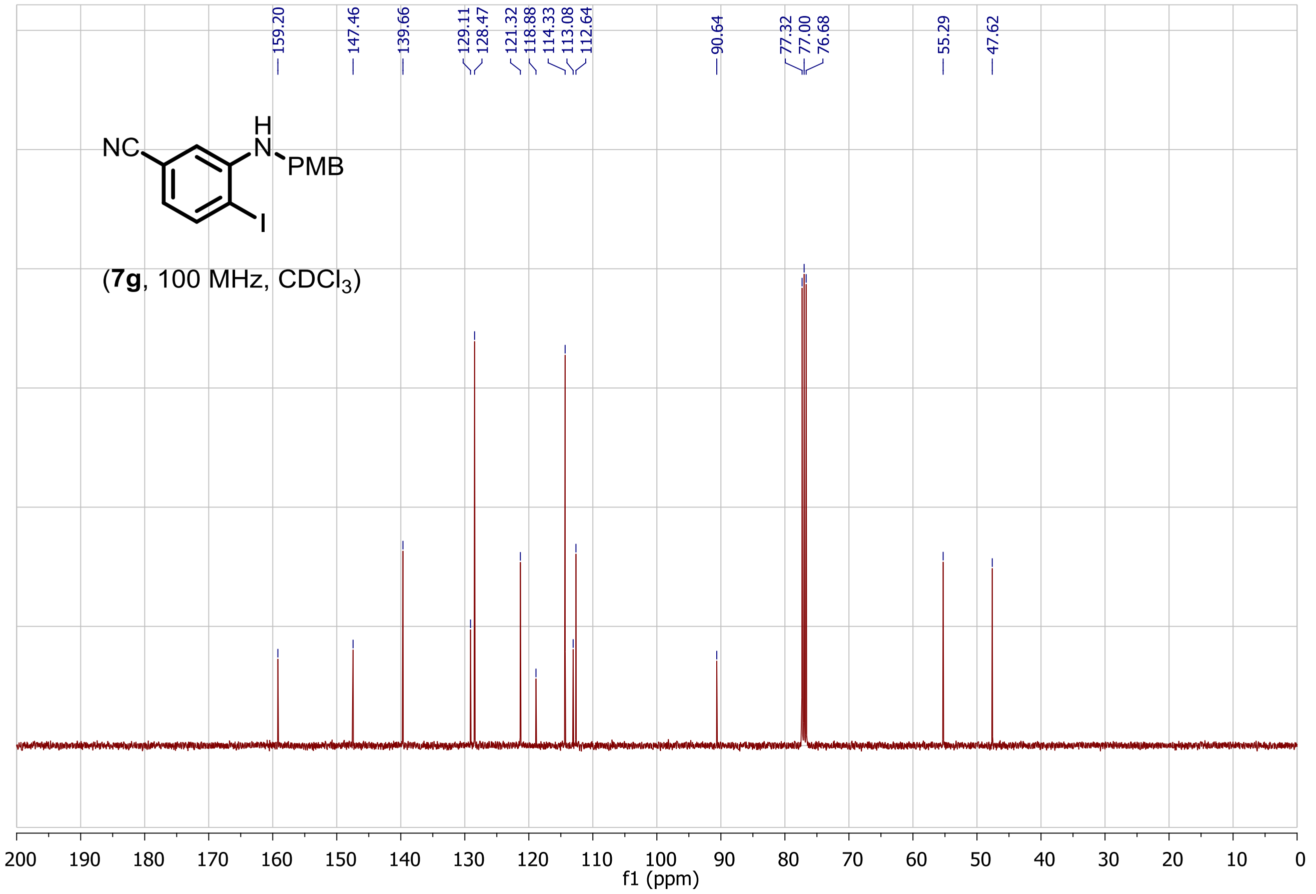
NC
H
N
PMB
I
(7g, 100 MHz, CDCl3)
159.20
147.46
139.66
129.11
128.47
121.32
118.88
114.33
113.08
112.64
90.64
77.32
77.00
76.68
55.29
47.62
f1 (ppm)

3.2. ^{1}H and ^{13}C NMR spectra of sulfonamides

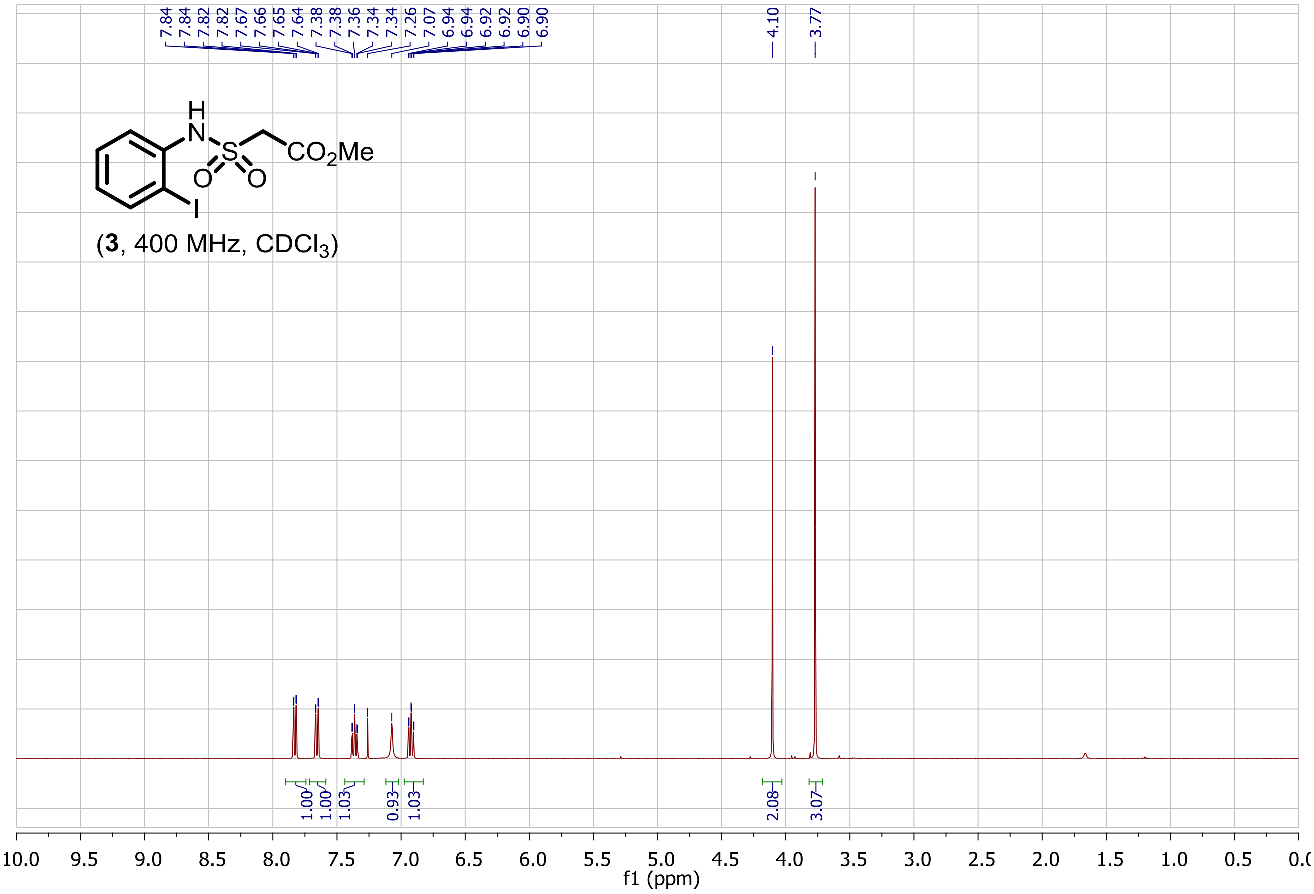

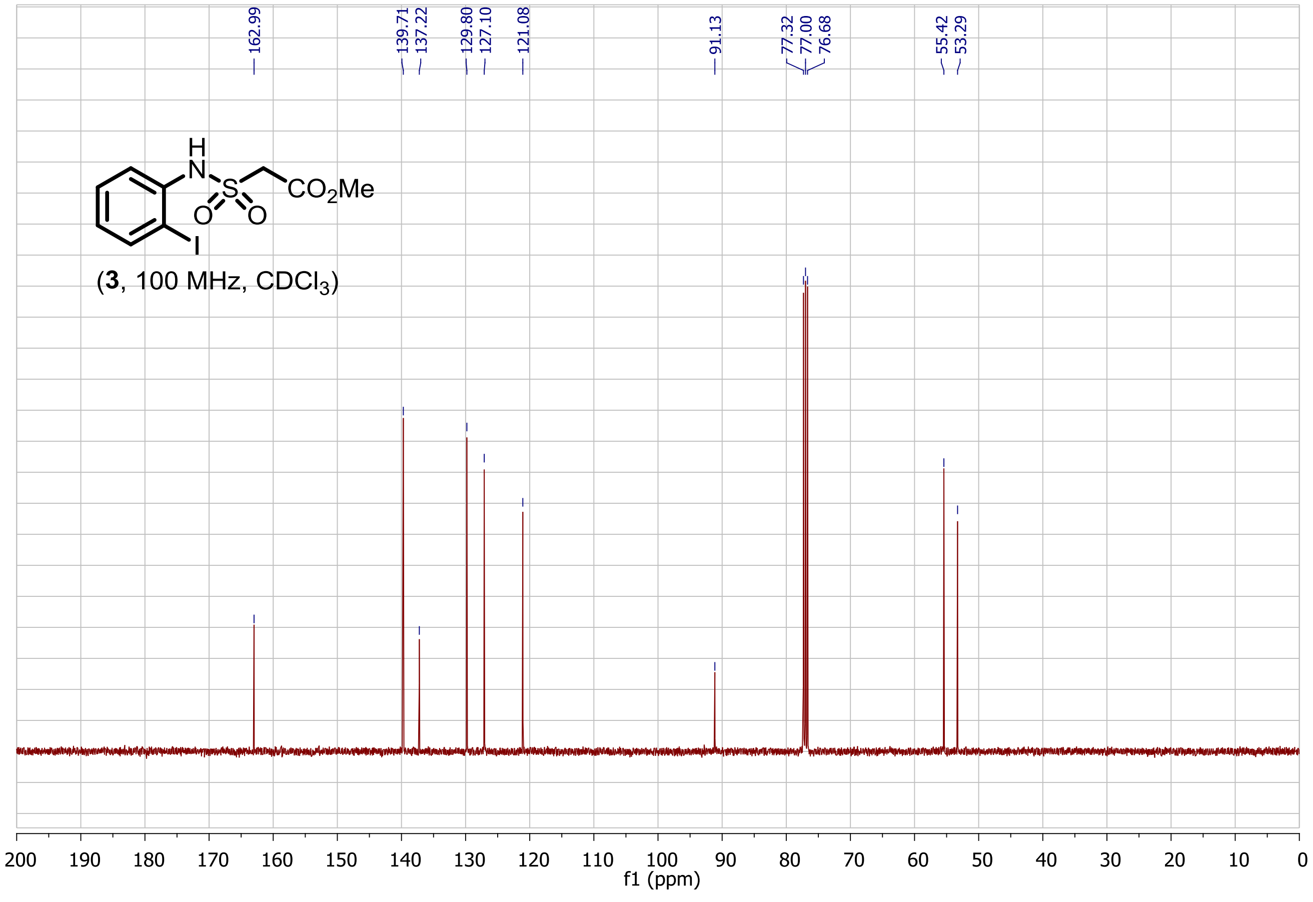

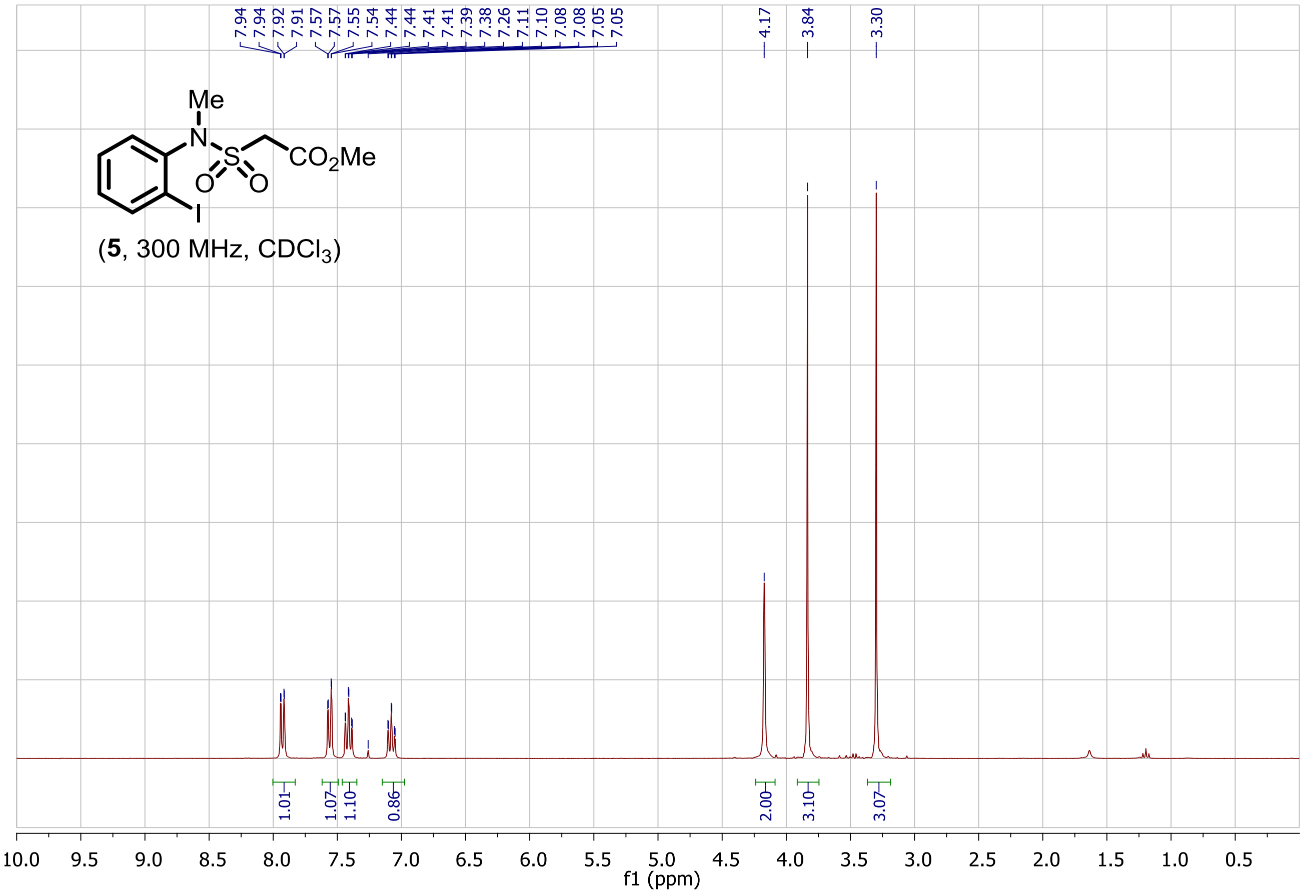
7.94
7.94
7.92
7.91
7.57
7.57
7.55
7.54
7.44
7.44
7.41
7.39
7.38
7.26
7.11
7.10
7.08
7.08
7.05
7.05
4.17
3.84
3.30
Me
N
S
O
O
CO_2Me
I
(**5**, 300 MHz, $CDCl_3$)
1.01
1.07
1.10
0.86
2.00
3.10
3.07
f1 (ppm)

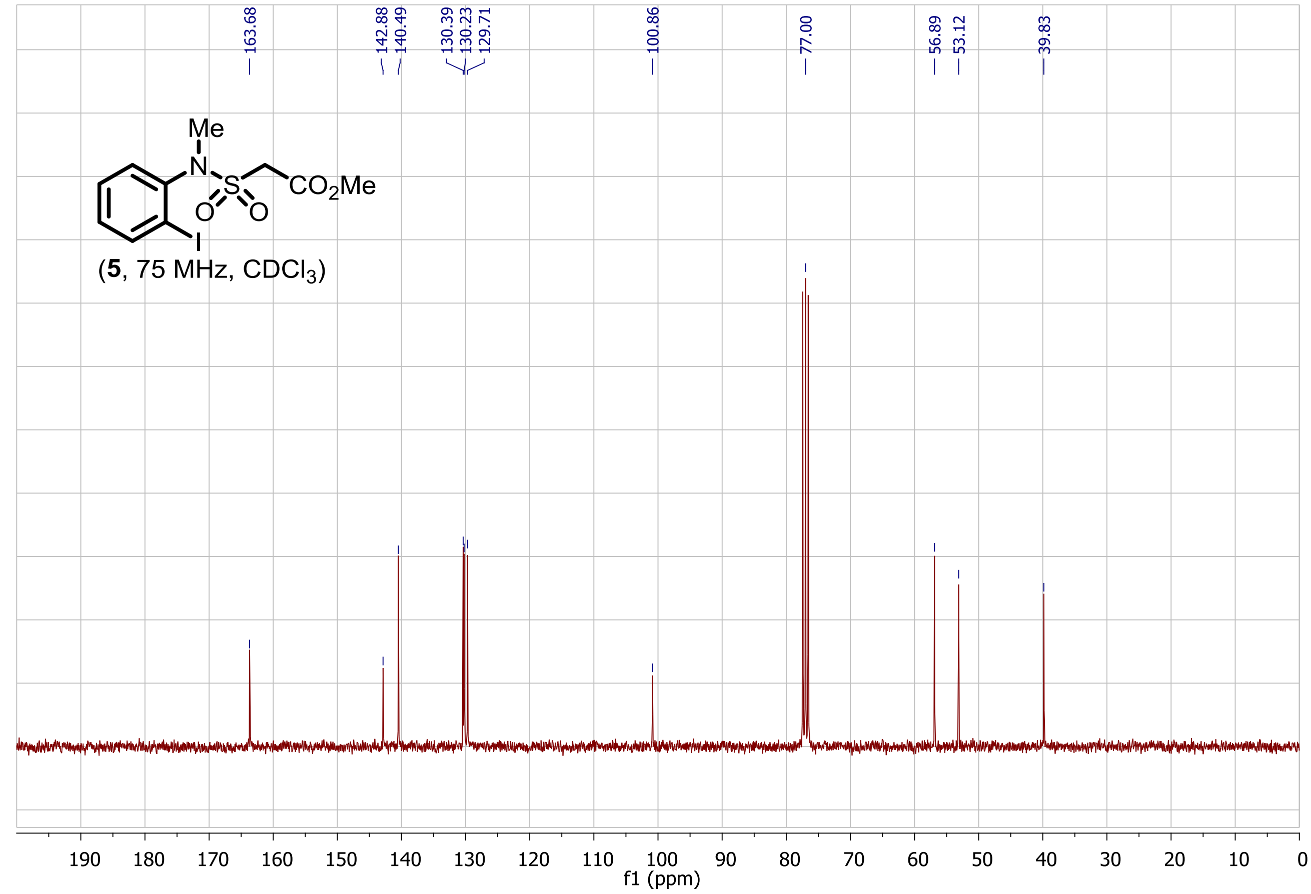
163.68
142.88
140.49
130.39
130.23
129.71
100.86
77.00
56.89
53.12
39.83
Me
N
S
O
O
CO_2Me
I
(**5**, 75 MHz, $CDCl_3$)
f1 (ppm)

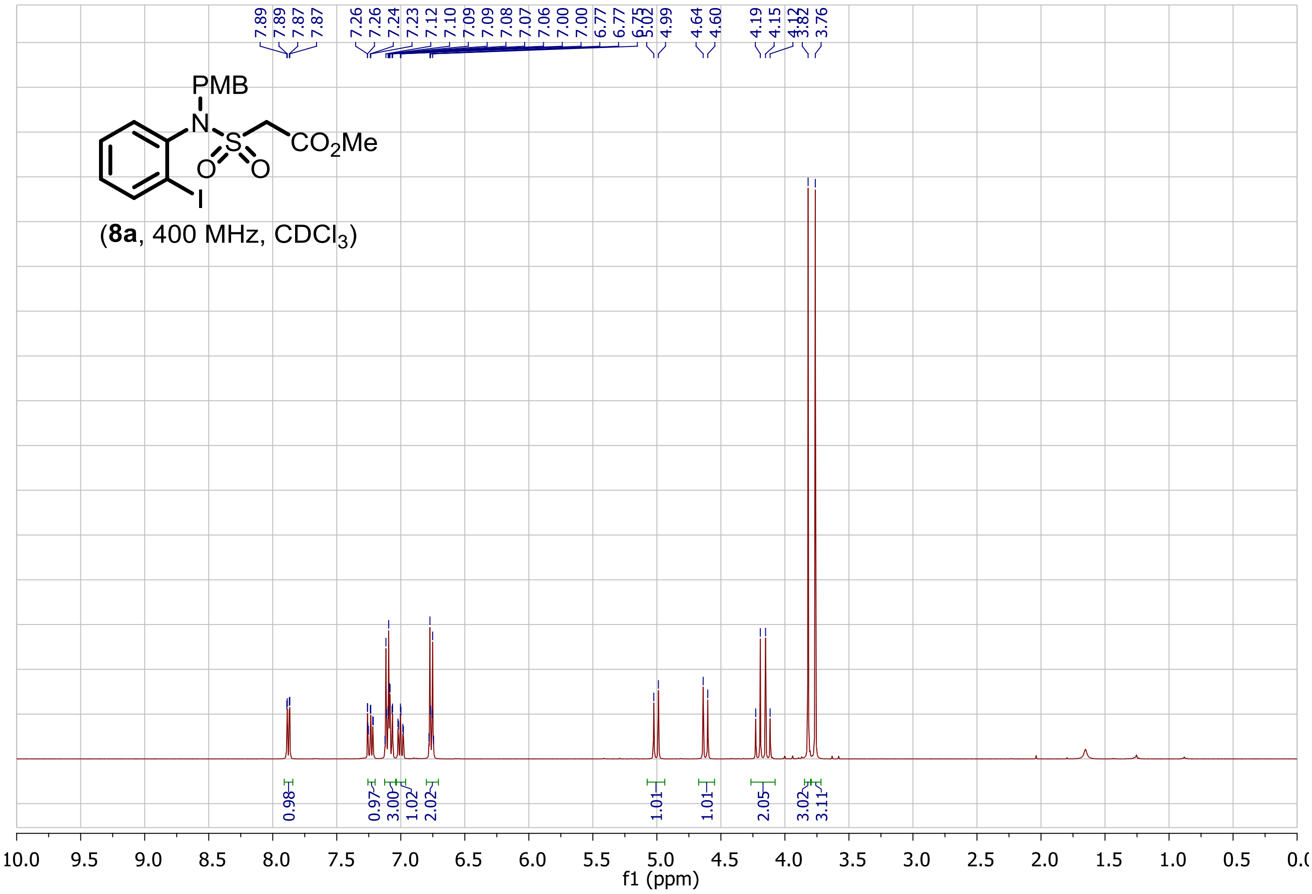
7.89
7.89
7.87
7.87
7.26
7.26
7.24
7.23
7.12
7.10
7.09
7.09
7.08
7.07
7.06
7.00
7.00
6.77
6.75
5.02
4.99
4.64
4.60
4.19
4.15
4.12
3.82
3.76
PMB
N
S
O
O
CO2Me
I
(8a, 400 MHz, CDCl3)
0.98
0.97
3.00
1.02
2.02
1.01
1.01
2.05
3.02
3.11
f1 (ppm)

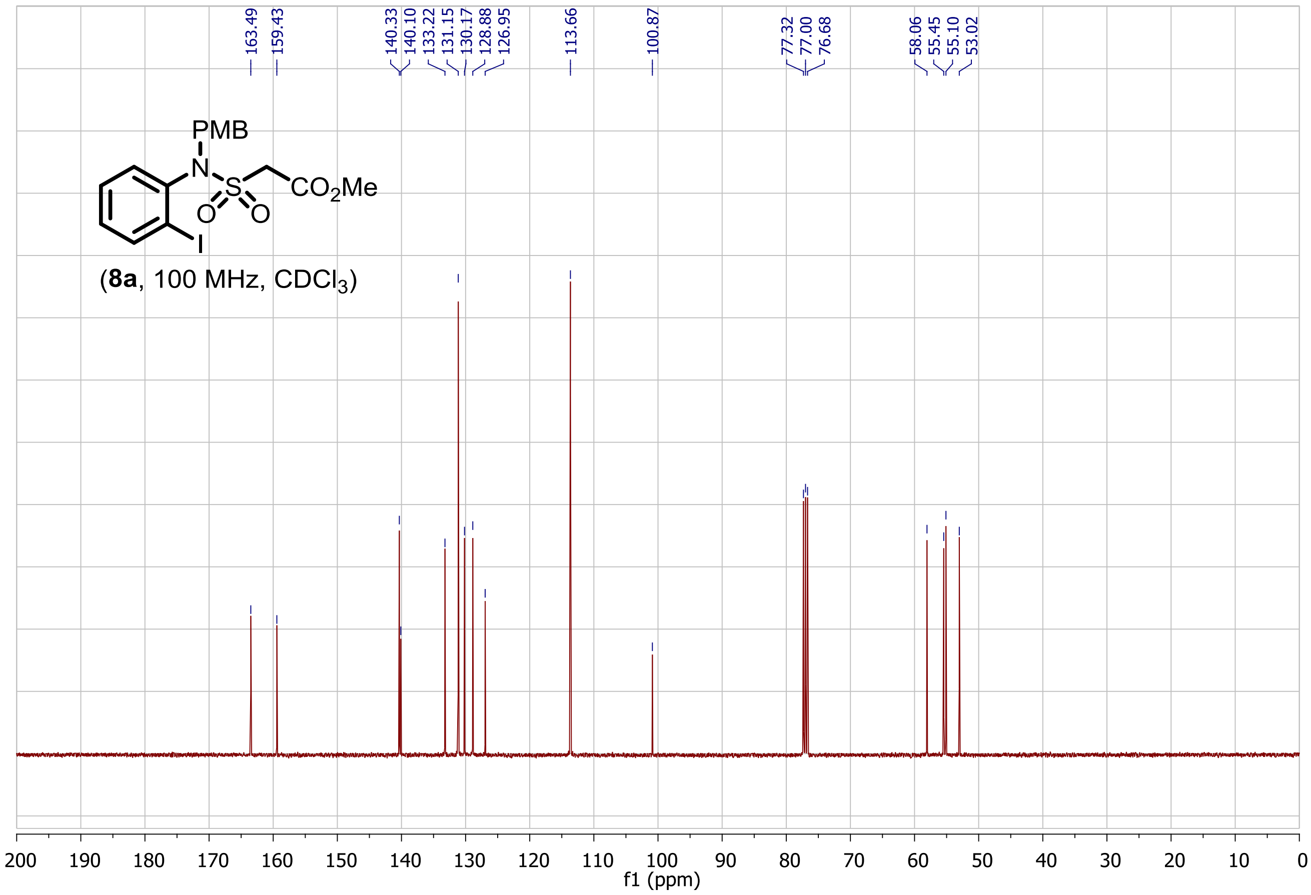
163.49
159.43
140.33
140.10
133.22
131.15
130.17
128.88
126.95
113.66
100.87
77.32
77.00
76.68
58.06
55.45
55.10
53.02
PMB
N
S
O
O
CO2Me
I
(8a, 100 MHz, CDCl3)
f1 (ppm)

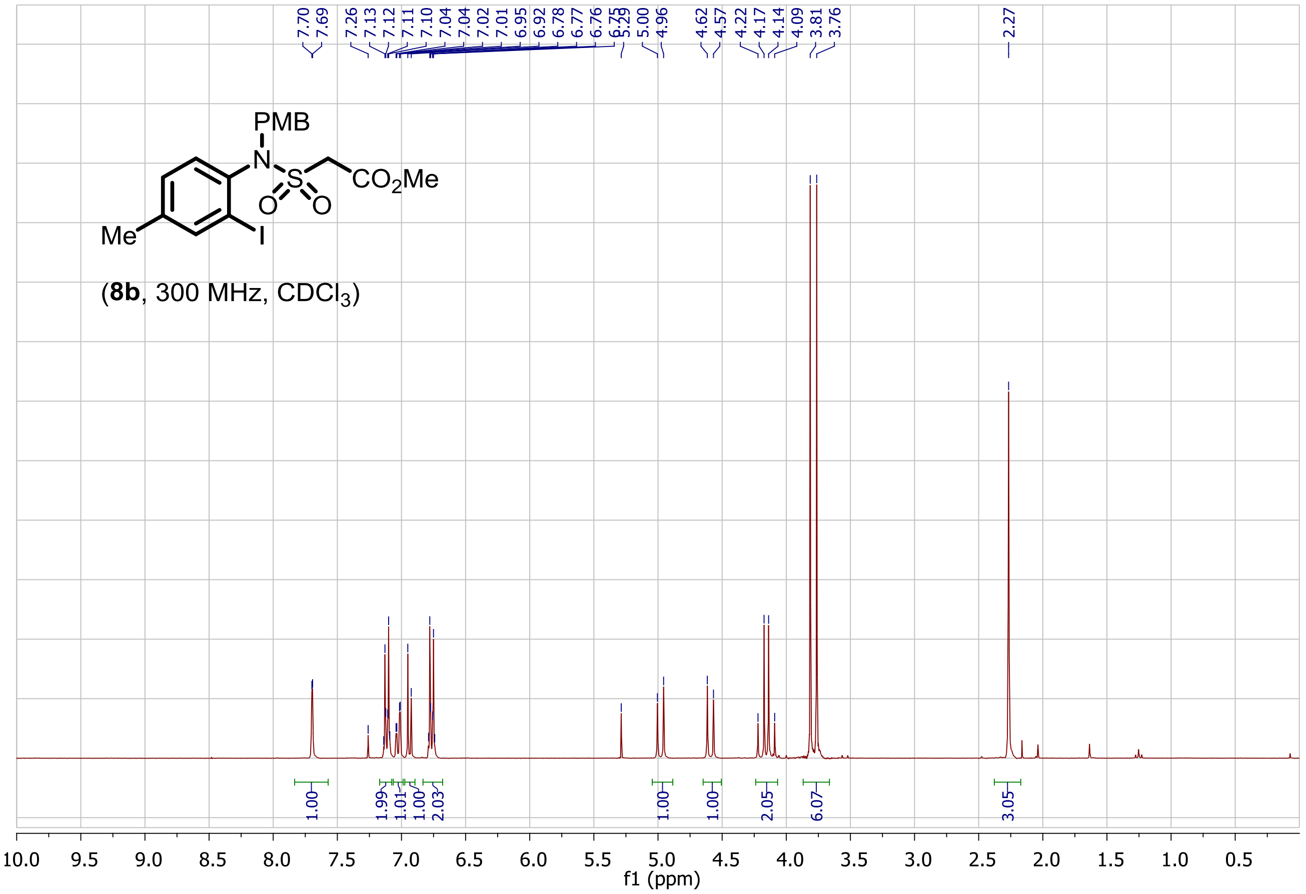
PMB
CO2Me
Me
I
(8b, 300 MHz, CDCl3)
7.70 7.69 7.26 7.13 7.12 7.11 7.10 7.04 7.04 7.02 7.01 6.95 6.92 6.78 6.77 6.76 6.75 5.29 5.00 4.96 4.62 4.57 4.22 4.17 4.14 4.09 3.81 3.76 2.27
1.00 1.99 1.01 1.00 2.03 1.00 1.00 2.05 6.07 3.05
f1 (ppm)

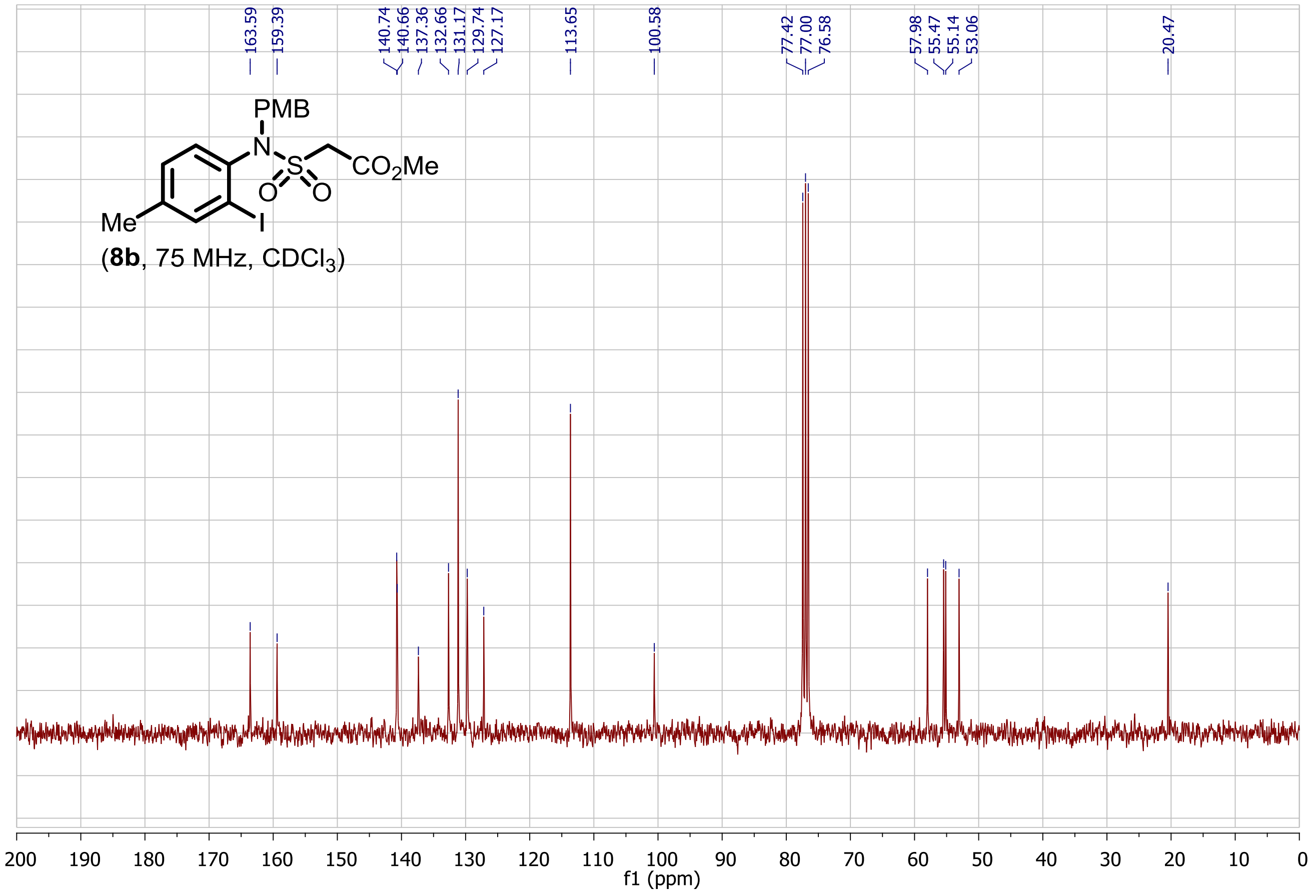
PMB
CO2Me
Me
I
(8b, 75 MHz, CDCl3)
163.59 159.39 140.74 140.66 137.36 132.66 131.17 129.74 127.17 113.65 100.58 77.42 77.00 76.58 57.98 55.47 55.14 53.06 20.47
f1 (ppm)

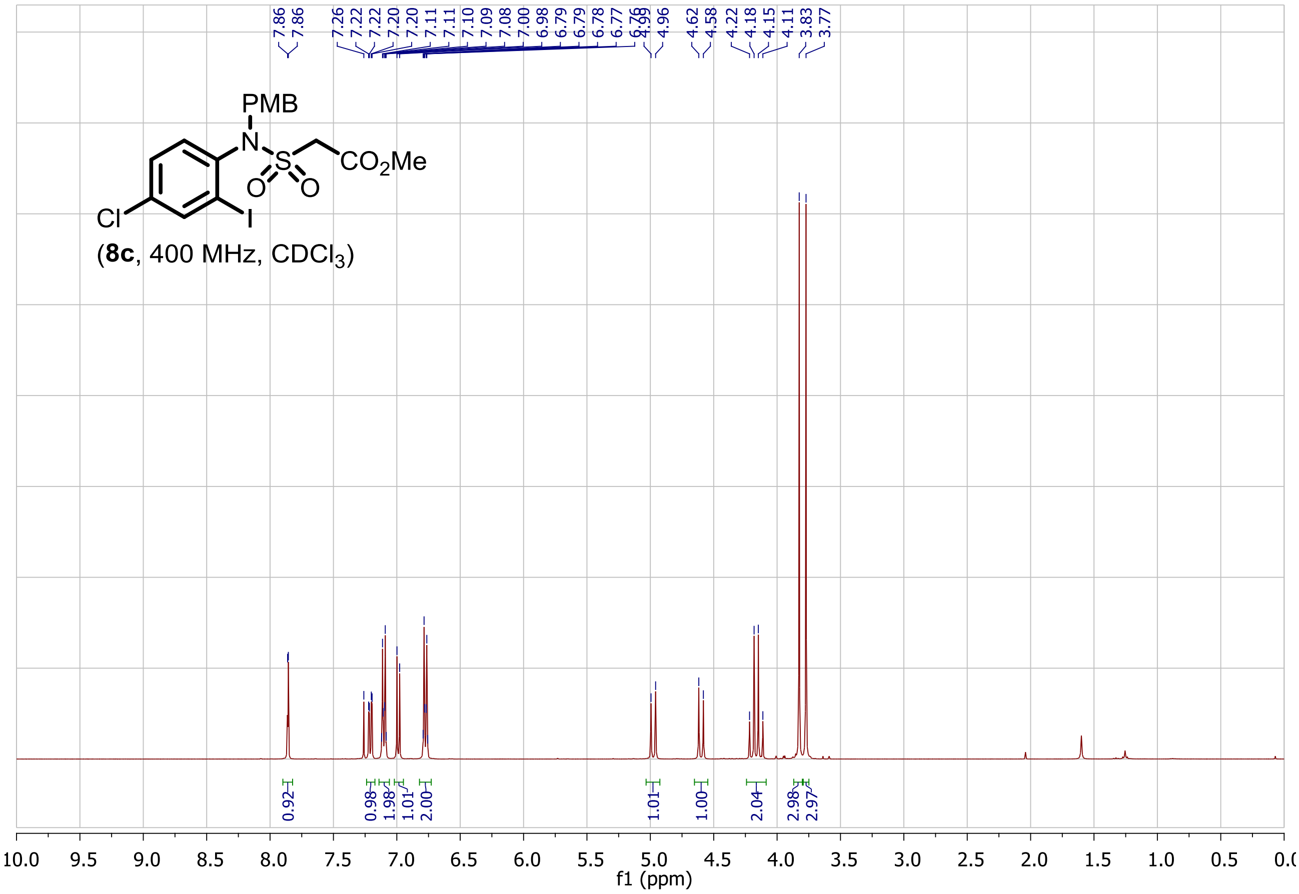
7.86
7.86
7.26
7.22
7.22
7.20
7.20
7.11
7.11
7.10
7.09
7.08
7.00
6.98
6.79
6.79
6.78
6.77
4.96
4.62
4.58
4.22
4.18
4.15
4.11
3.83
3.77
PMB
N
S
O
O
CO2Me
Cl
I
(8c, 400 MHz, CDCl3)
0.92
0.98
1.98
1.01
2.00
1.01
1.00
2.04
2.98
2.97
f1 (ppm)

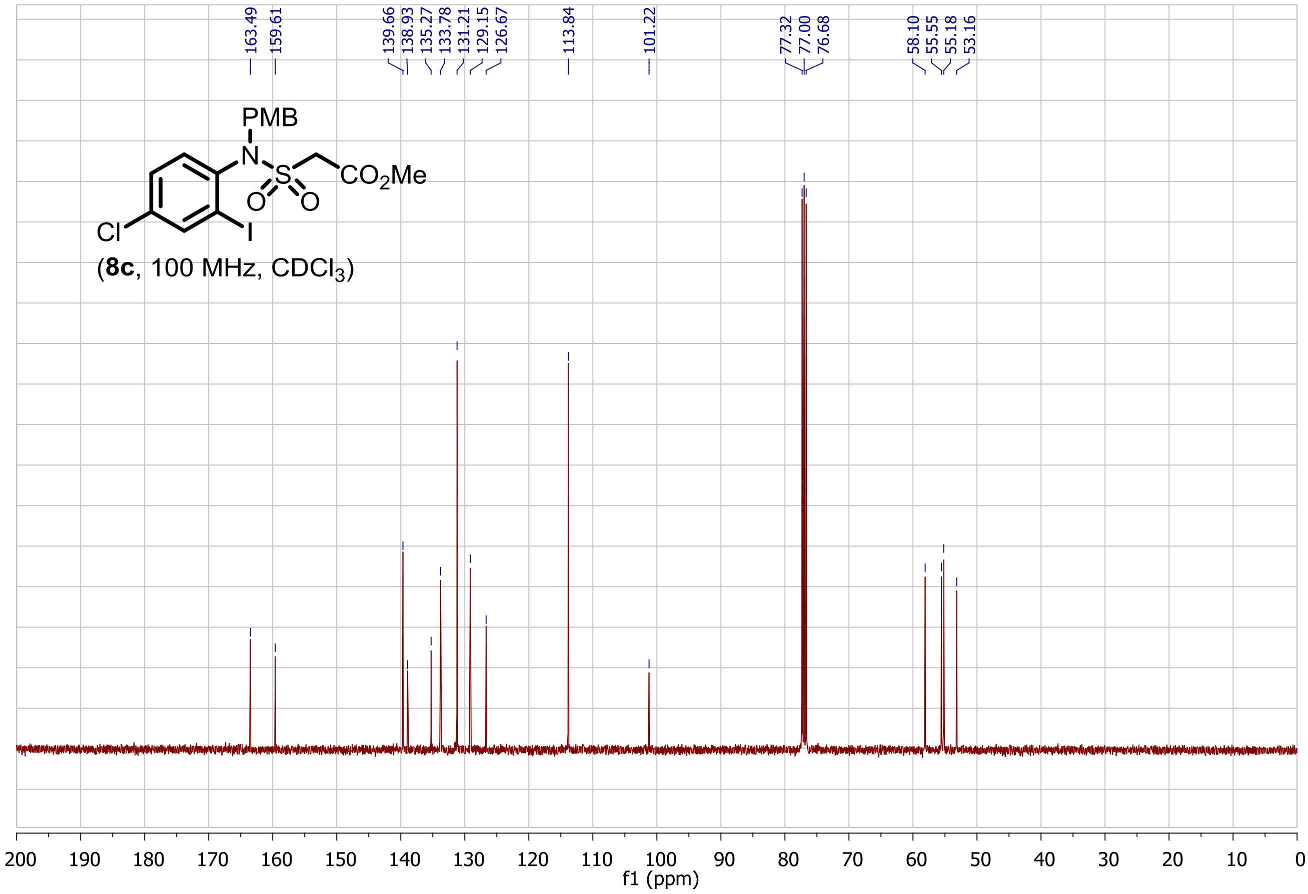
163.49
159.61
139.66
138.93
135.27
133.78
131.21
129.15
126.67
113.84
101.22
77.32
77.00
76.68
58.10
55.55
55.18
53.16
PMB
N
S
O
O
CO2Me
Cl
I
(8c, 100 MHz, CDCl3)
f1 (ppm)

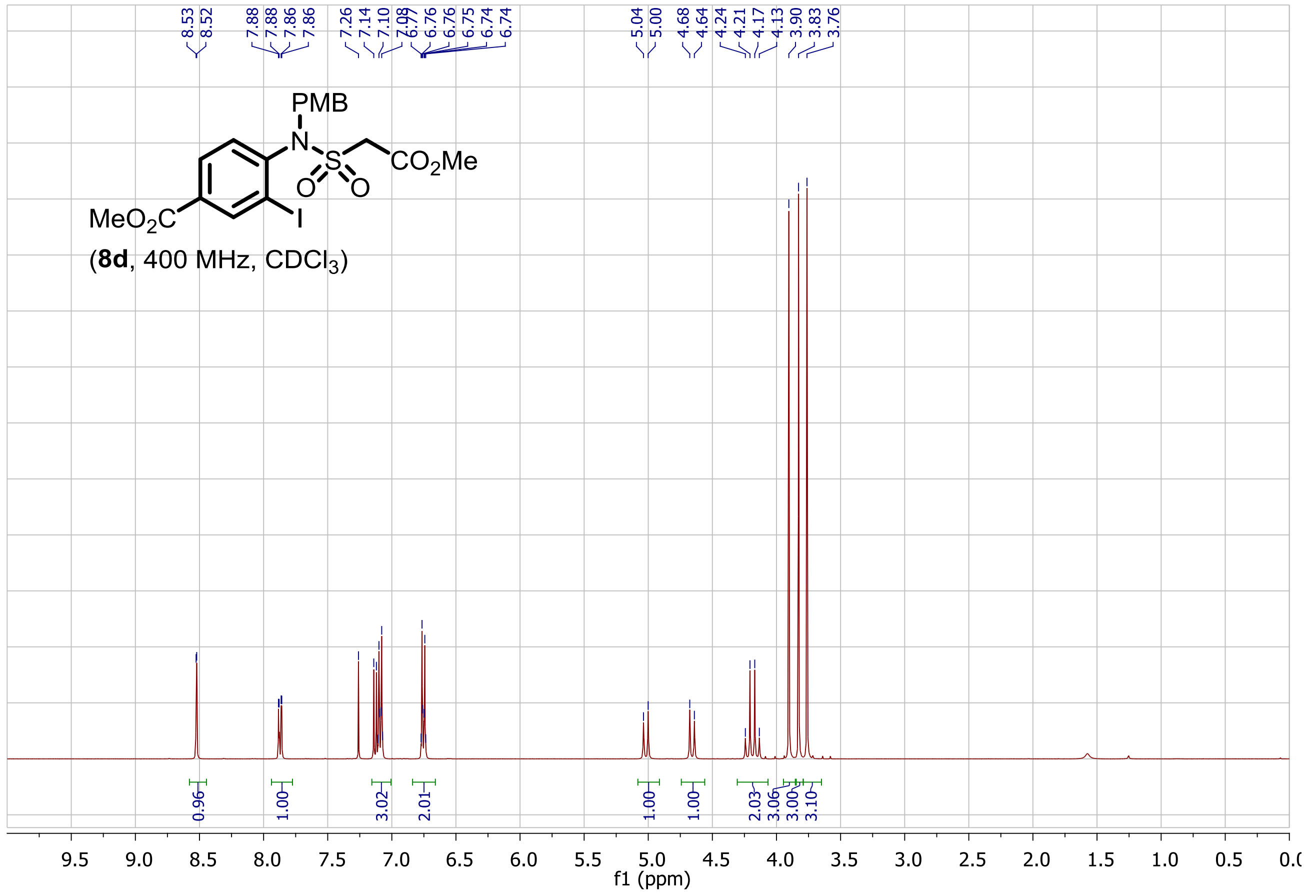
8.53
8.52
7.88
7.88
7.86
7.86
7.26
7.14
7.10
7.08
6.77
6.76
6.76
6.75
6.74
6.74
5.04
5.00
4.68
4.64
4.24
4.21
4.17
4.13
3.90
3.83
3.76
PMB
N
S
O
O
CO2Me
MeO2C
I
(8d, 400 MHz, CDCl3)
0.96
1.00
3.02
2.01
1.00
1.00
2.03
3.06
3.00
3.10
f1 (ppm)

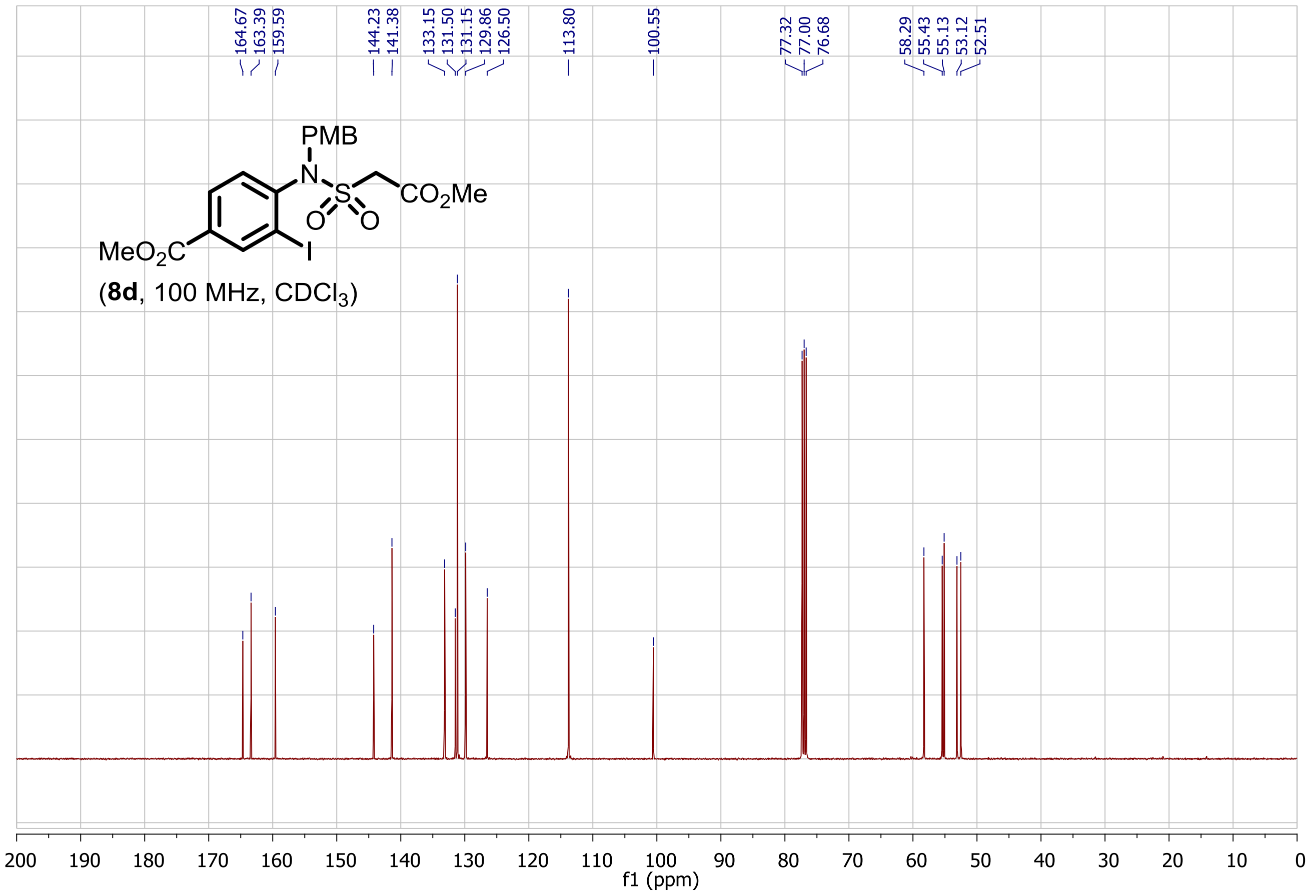
164.67
163.39
159.59
144.23
141.38
133.15
131.50
131.15
129.86
126.50
113.80
100.55
77.32
77.00
76.68
58.29
55.43
55.13
53.12
52.51
PMB
N
S
O
O
CO2Me
MeO2C
I
(8d, 100 MHz, CDCl3)
f1 (ppm)

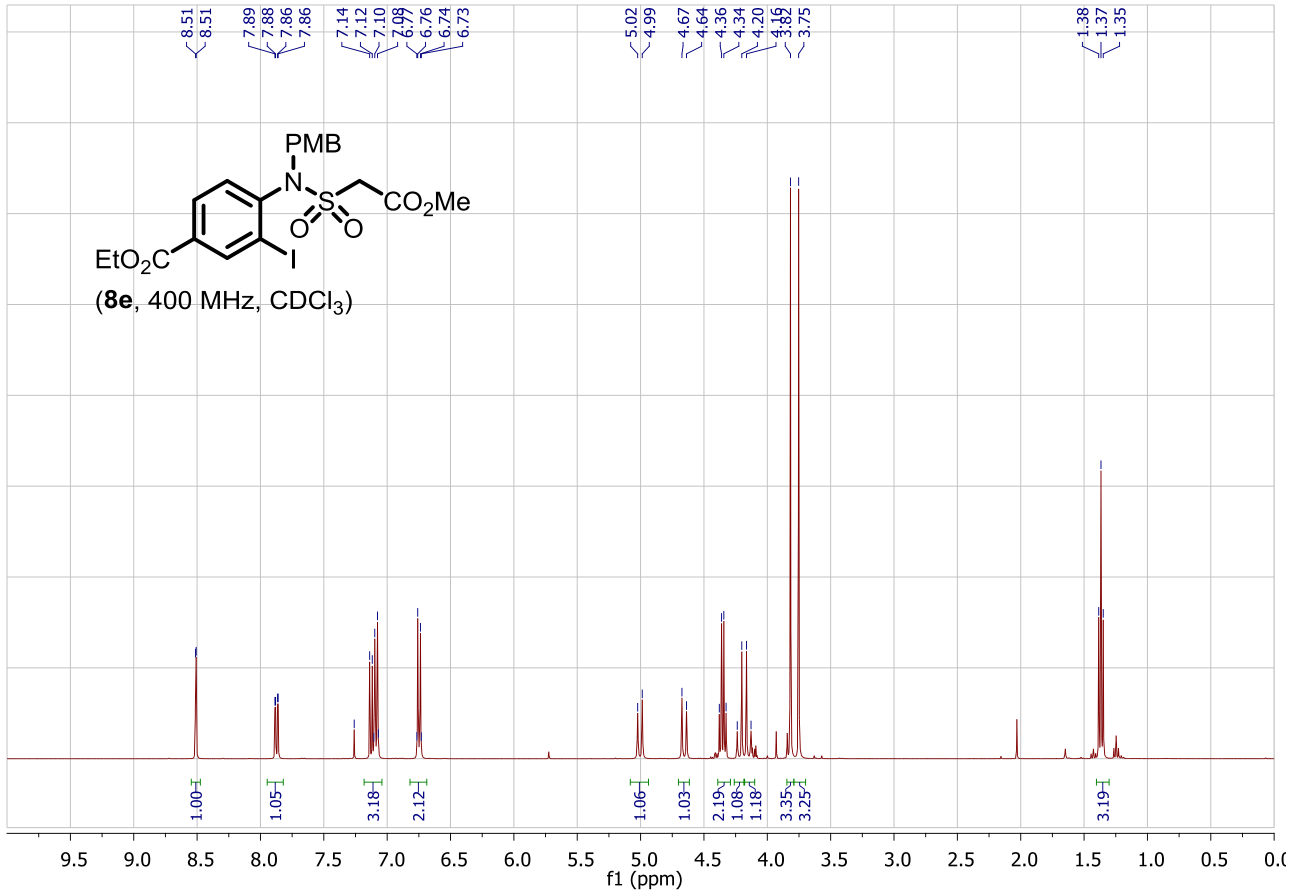
8.51
8.51
7.89
7.88
7.86
7.86
7.14
7.12
7.10
7.08
6.77
6.76
6.74
6.73
5.02
4.99
4.67
4.64
4.36
4.34
4.20
4.16
3.82
3.75
1.38
1.37
1.35
PMB
N
S
O
O
CO2Me
EtO2C
I
(8e, 400 MHz, CDCl3)
1.00
1.05
3.18
2.12
1.06
1.03
2.19
1.08
1.18
3.35
3.25
3.19
f1 (ppm)

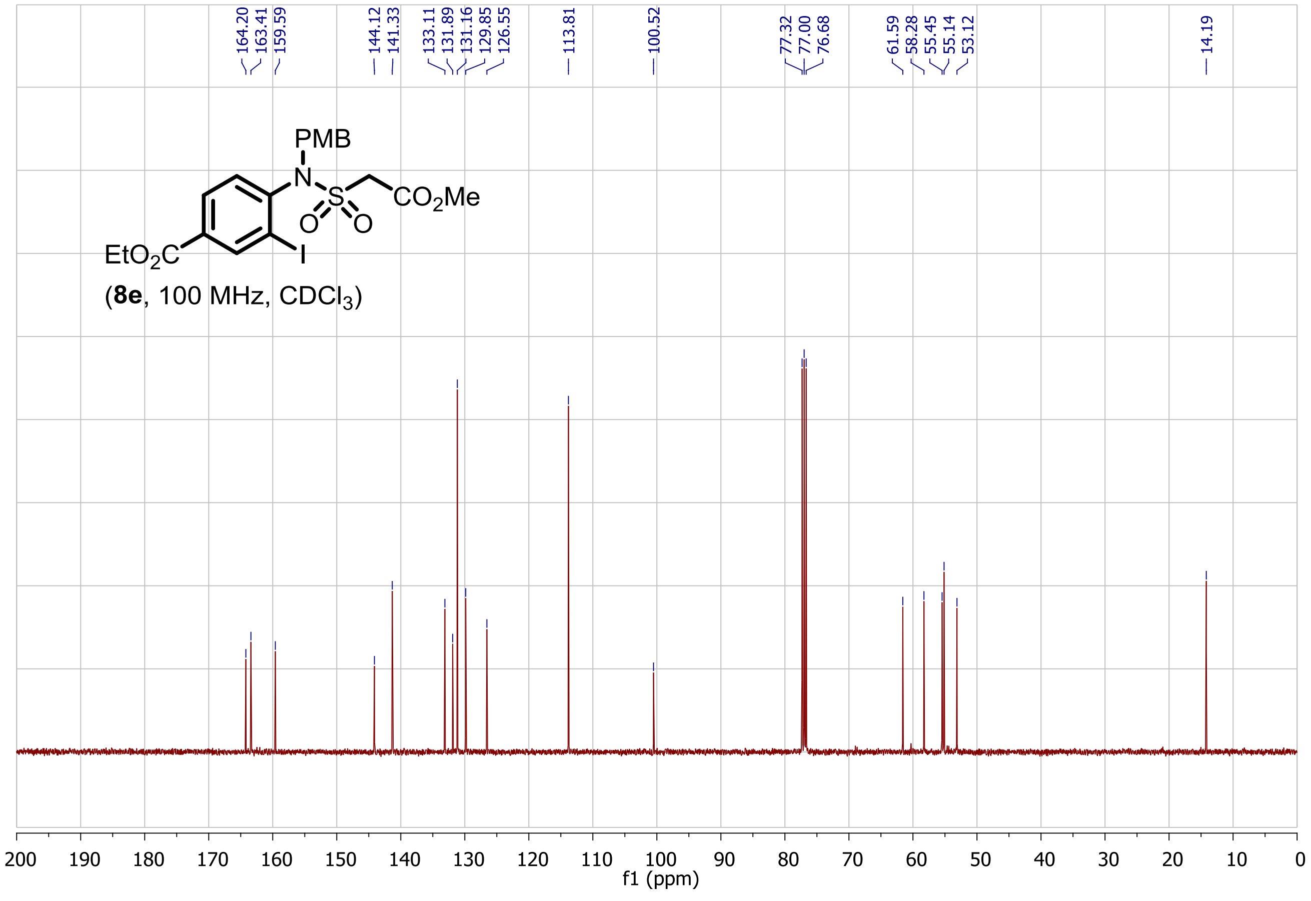
164.20
163.41
159.59
144.12
141.33
133.11
131.89
131.16
129.85
126.55
113.81
100.52
77.32
77.00
76.68
61.59
58.28
55.45
55.14
53.12
14.19
PMB
N
S
O
O
CO2Me
EtO2C
I
(8e, 100 MHz, CDCl3)
f1 (ppm)

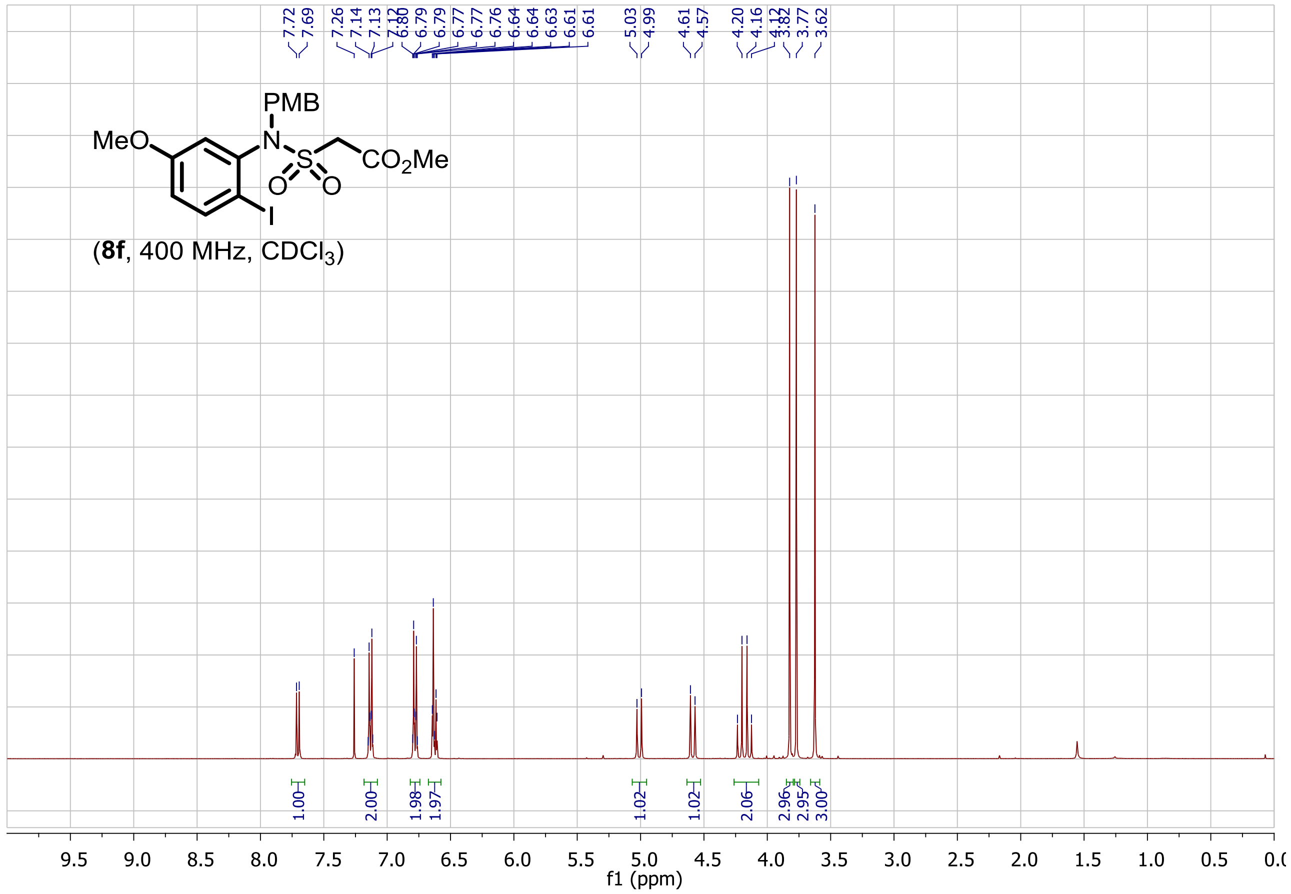
(8f, 400 MHz, CDCl3)
f1 (ppm)

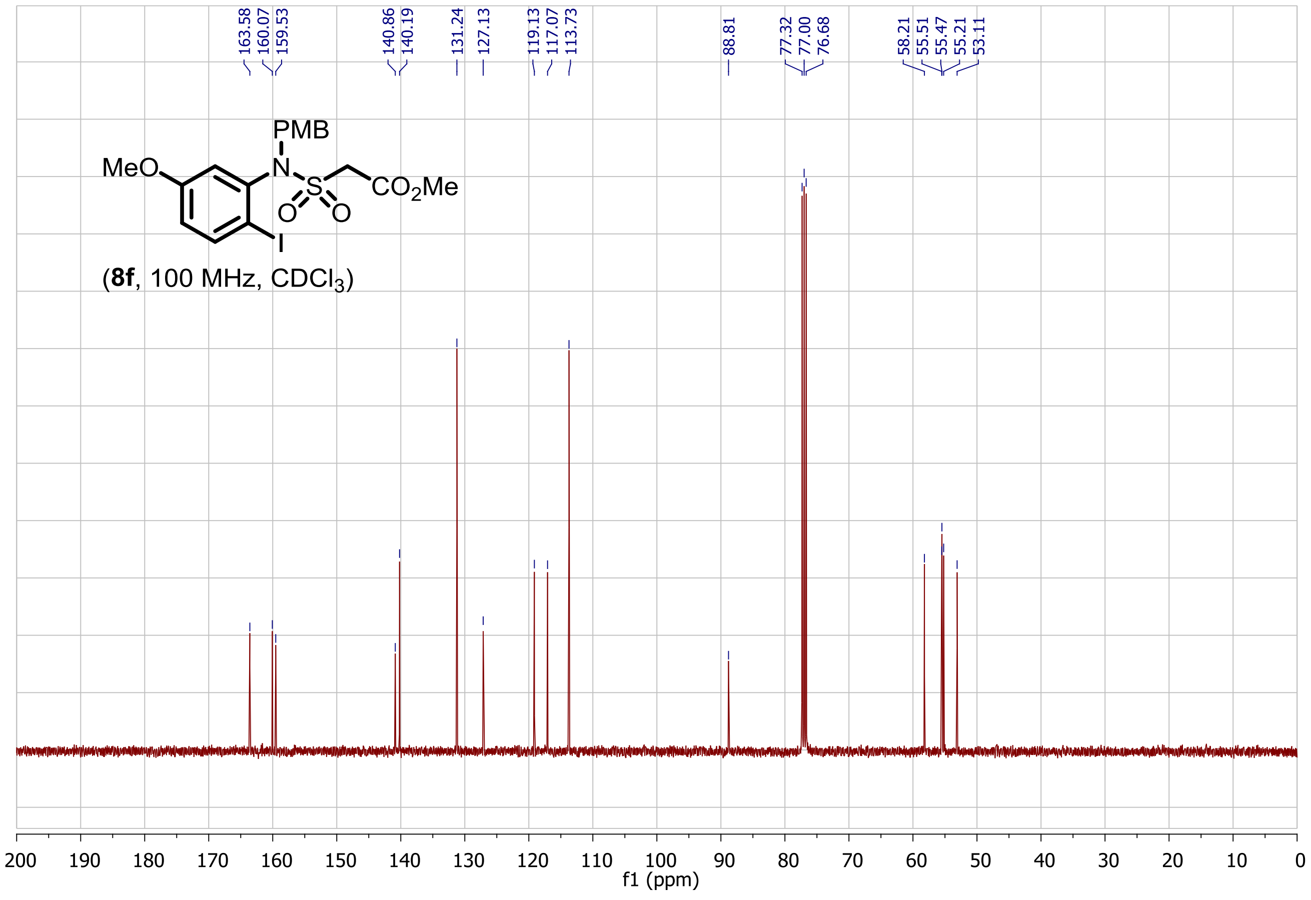
(8f, 100 MHz, CDCl3)
f1 (ppm)

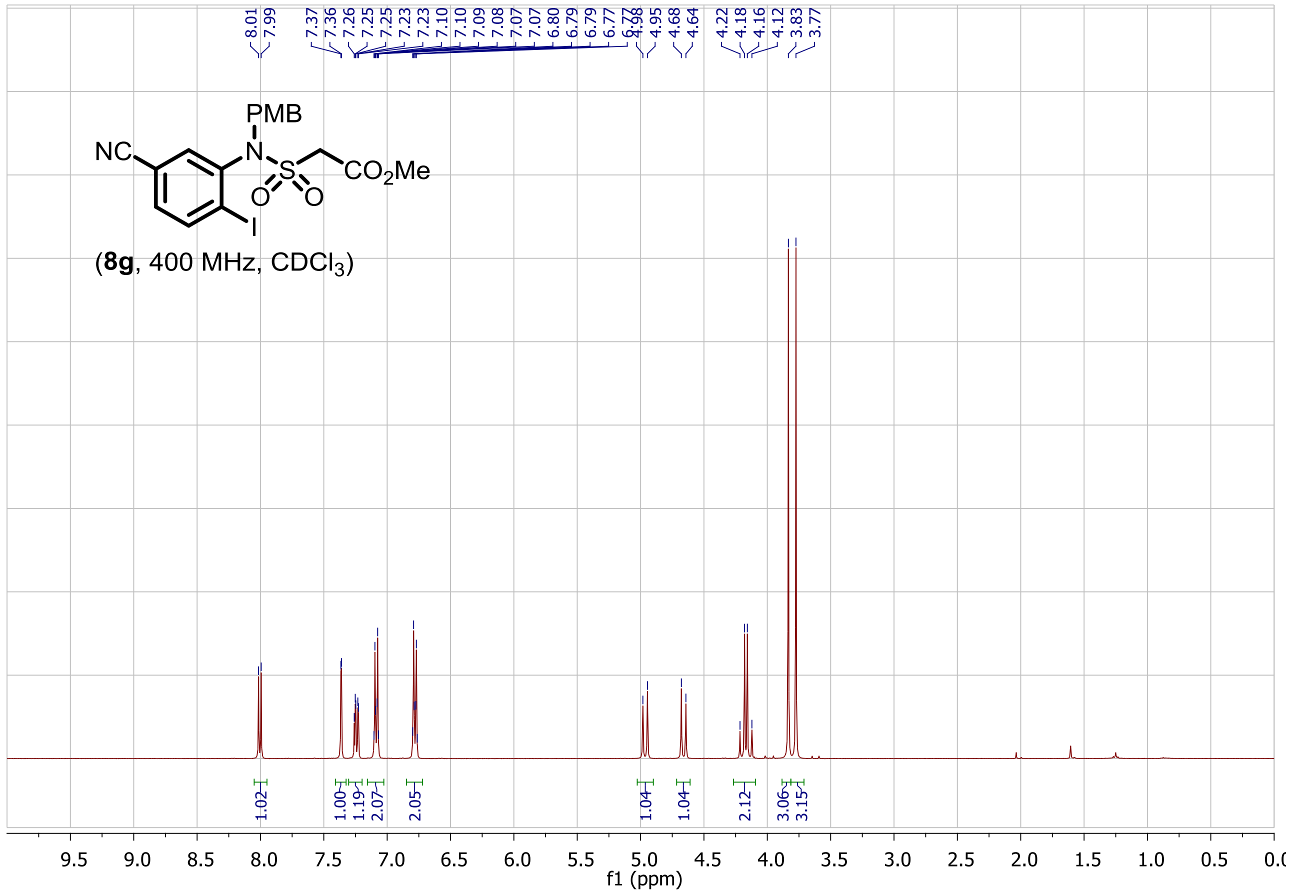
PMB
NC
N
S
CO2Me
O
O
I
(8g, 400 MHz, CDCl3)
f1 (ppm)

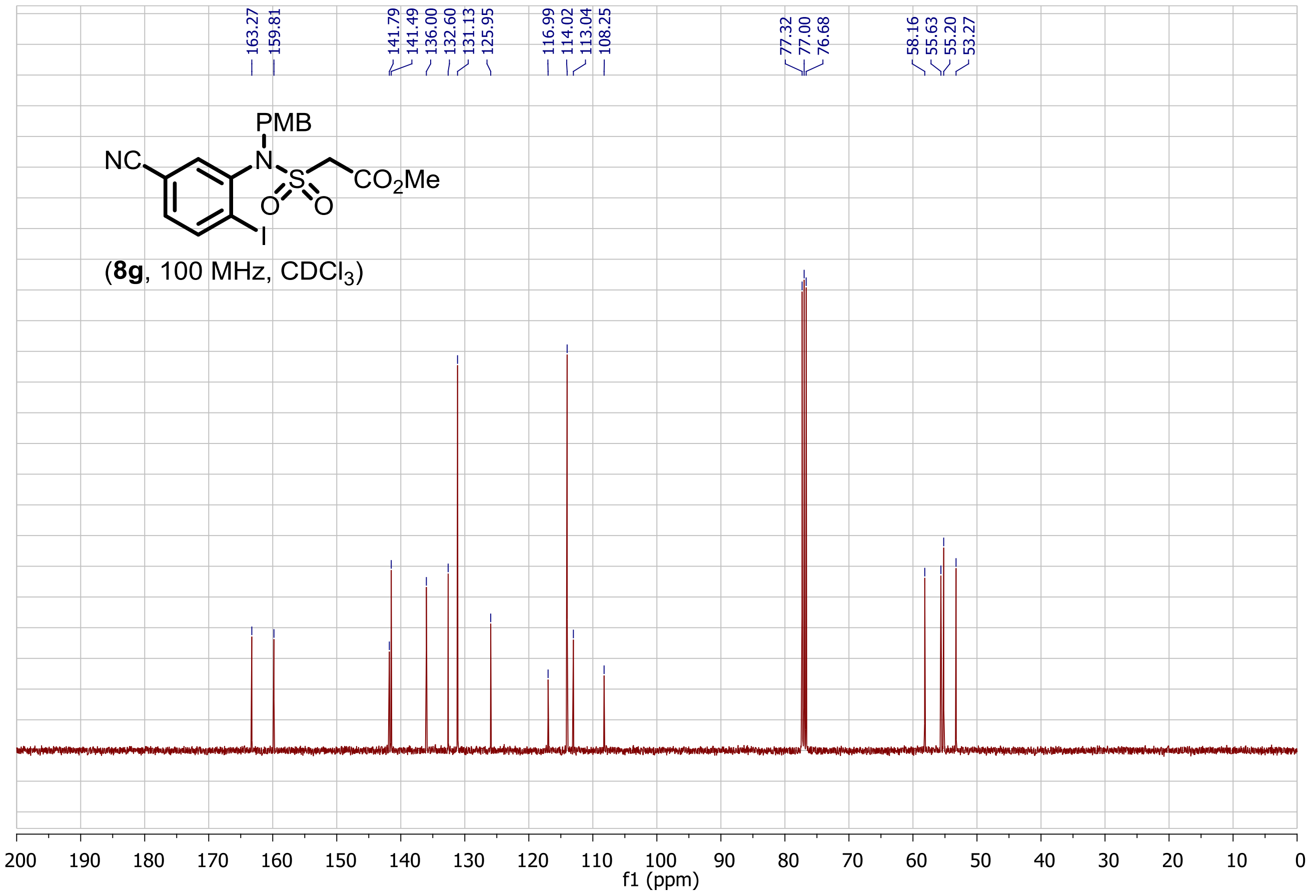
PMB
NC
N
S
CO2Me
O
O
I
(8g, 100 MHz, CDCl3)
f1 (ppm)

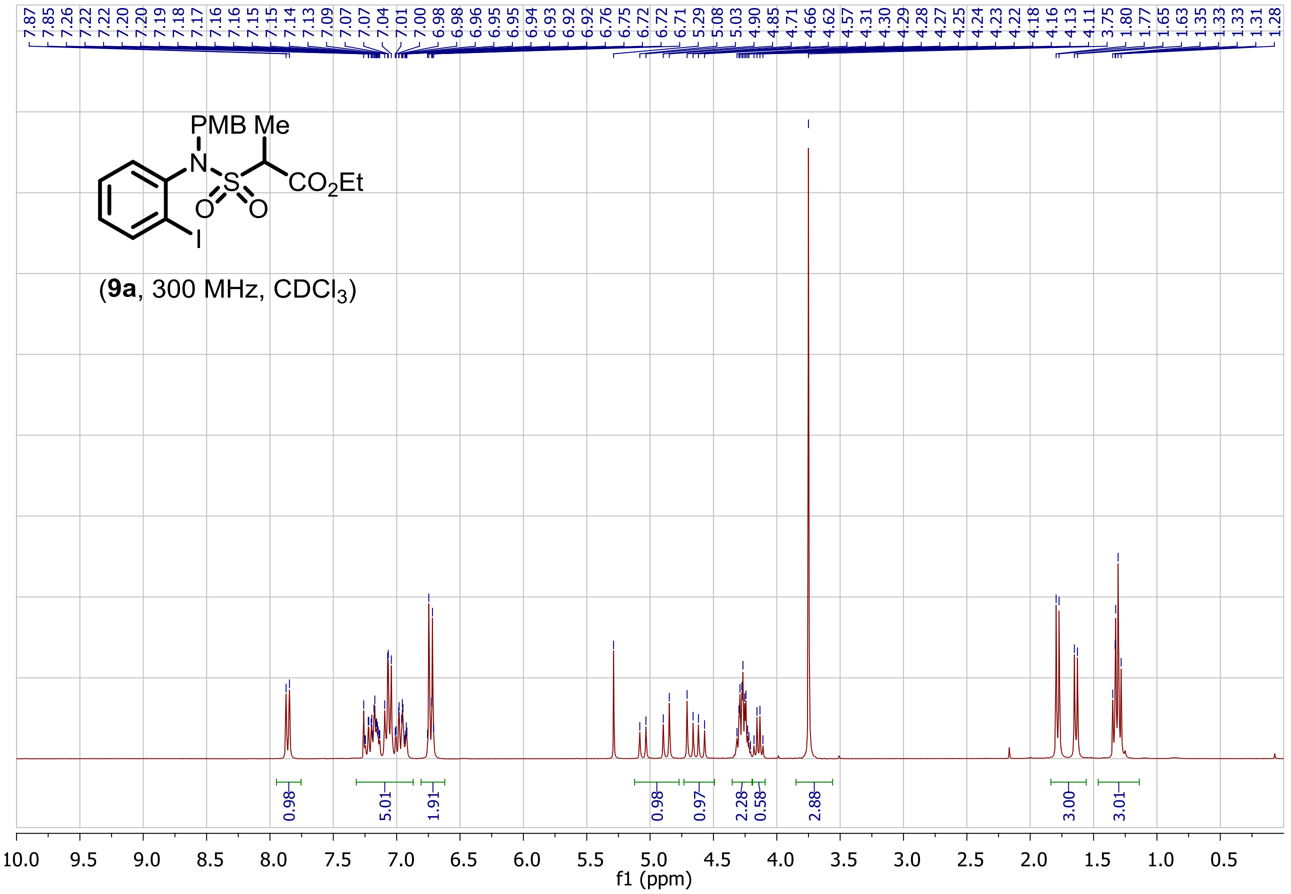
PMB Me
N
S
CO2Et
O O
I
(9a, 300 MHz, CDCl3)
f1 (ppm)

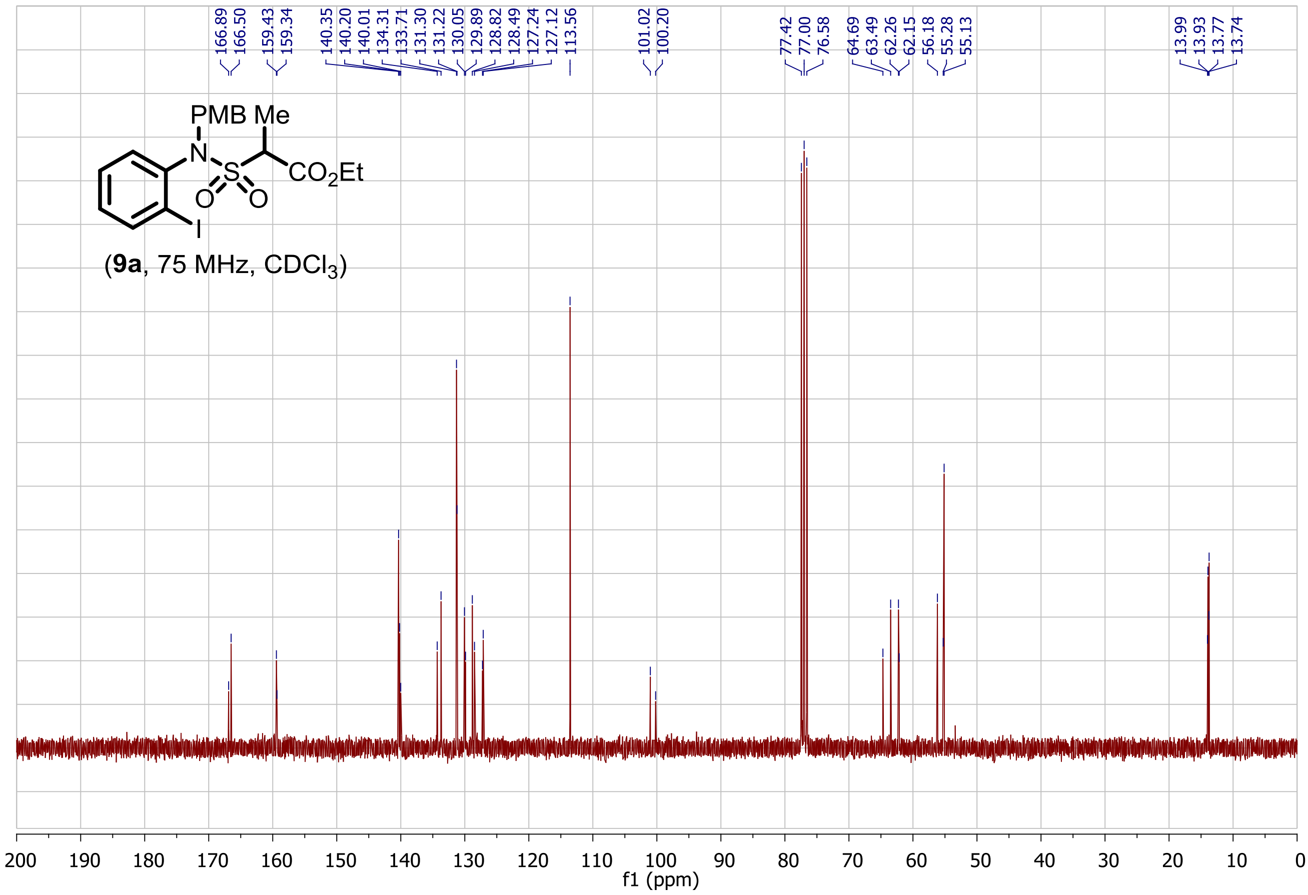
PMB Me
N
S
CO2Et
O O
I
(9a, 75 MHz, CDCl3)
f1 (ppm)

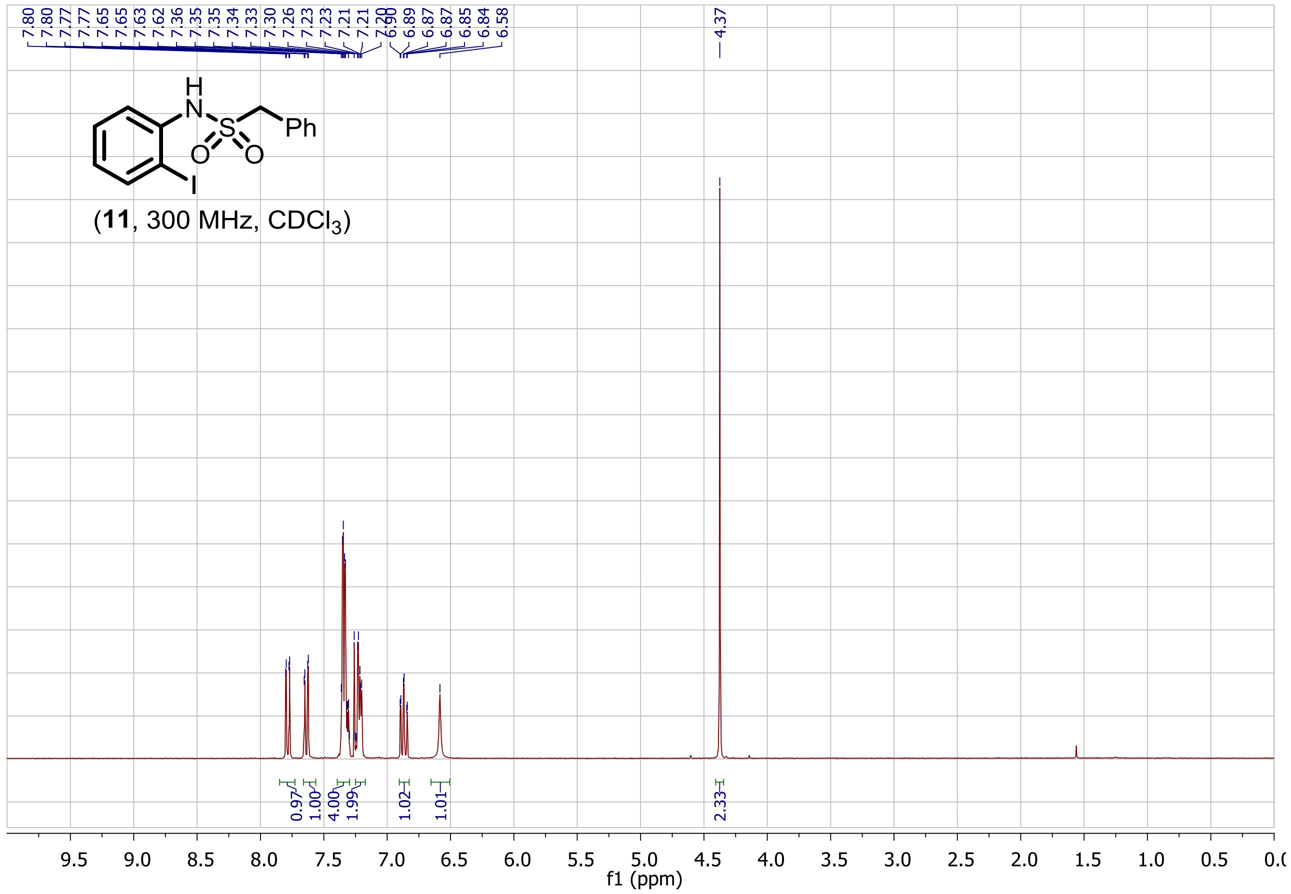
7.80
7.80
7.77
7.77
7.65
7.65
7.63
7.62
7.36
7.35
7.35
7.34
7.33
7.30
7.26
7.23
7.23
7.21
7.21
6.89
6.87
6.87
6.85
6.84
6.58
4.37
H
N
S
Ph
O
O
I
(11, 300 MHz, CDCl3)
0.97
1.00
4.00
1.99
1.02
1.01
2.33
f1 (ppm)

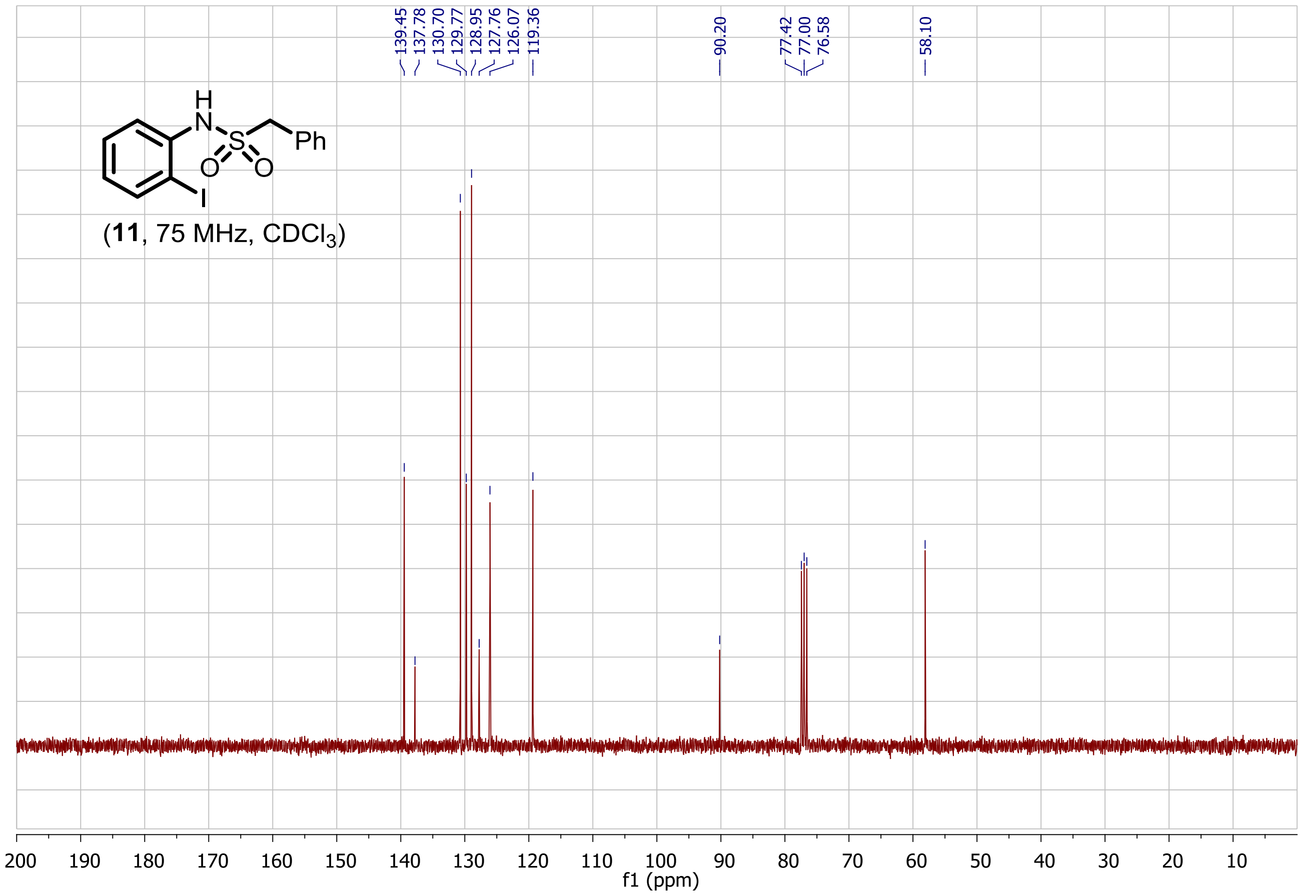
139.45
137.78
130.70
129.77
128.95
127.76
126.07
119.36
90.20
77.42
77.00
76.58
58.10
H
N
S
Ph
O
O
I
(11, 75 MHz, CDCl3)
f1 (ppm)

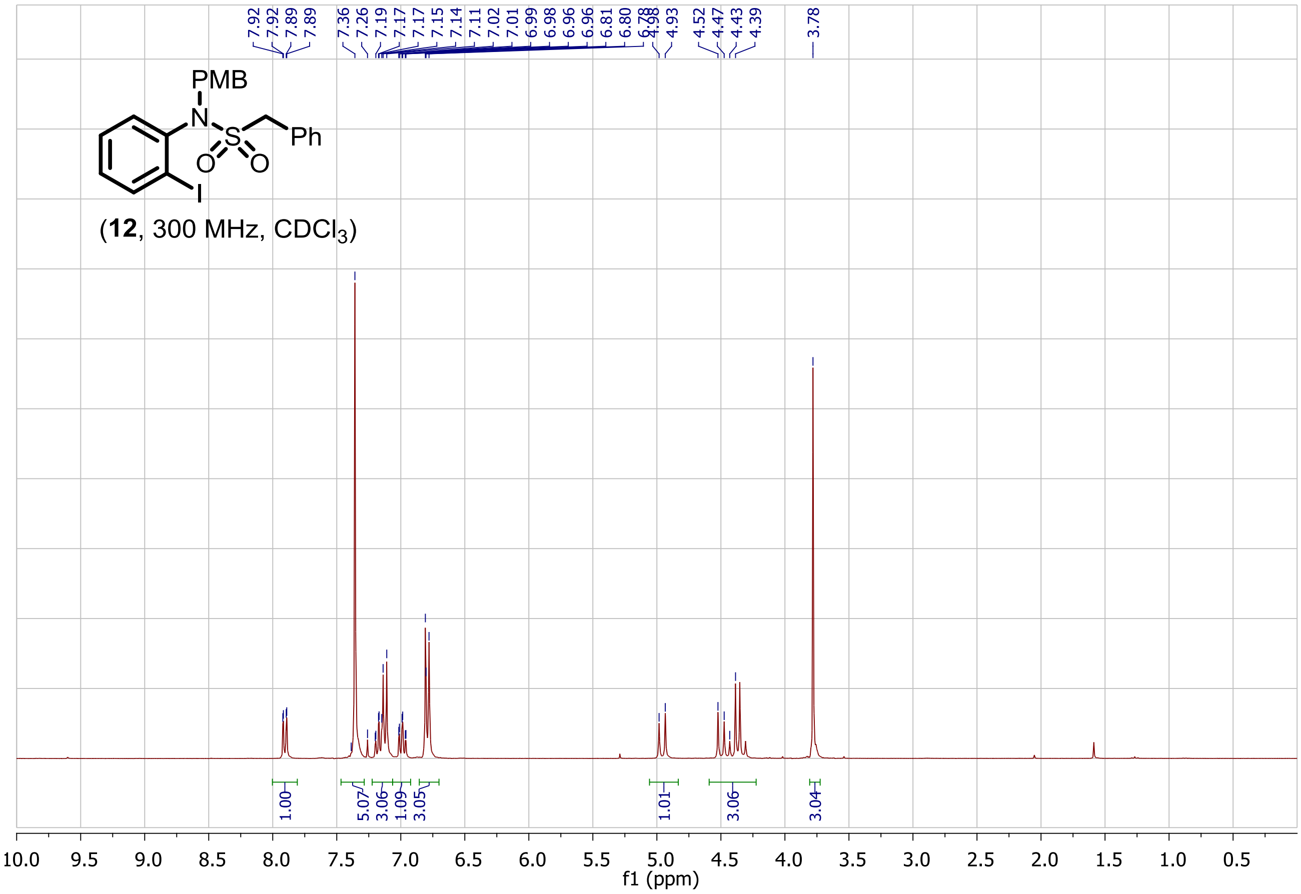
7.92 7.92 7.89 7.89 7.36 7.26 7.19 7.17 7.17 7.15 7.14 7.11 7.02 7.01 6.99 6.98 6.96 6.96 6.81 6.80 6.78 4.98 4.93 4.52 4.47 4.43 4.39 3.78
PMB
N
S
O O
Ph
I
(**12**, 300 MHz, $CDCl_3$)
1.00 5.07 3.06 1.09 3.05 1.01 3.06 3.04
10.0 9.5 9.0 8.5 8.0 7.5 7.0 6.5 6.0 5.5 5.0 4.5 4.0 3.5 3.0 2.5 2.0 1.5 1.0 0.5
f1 (ppm)

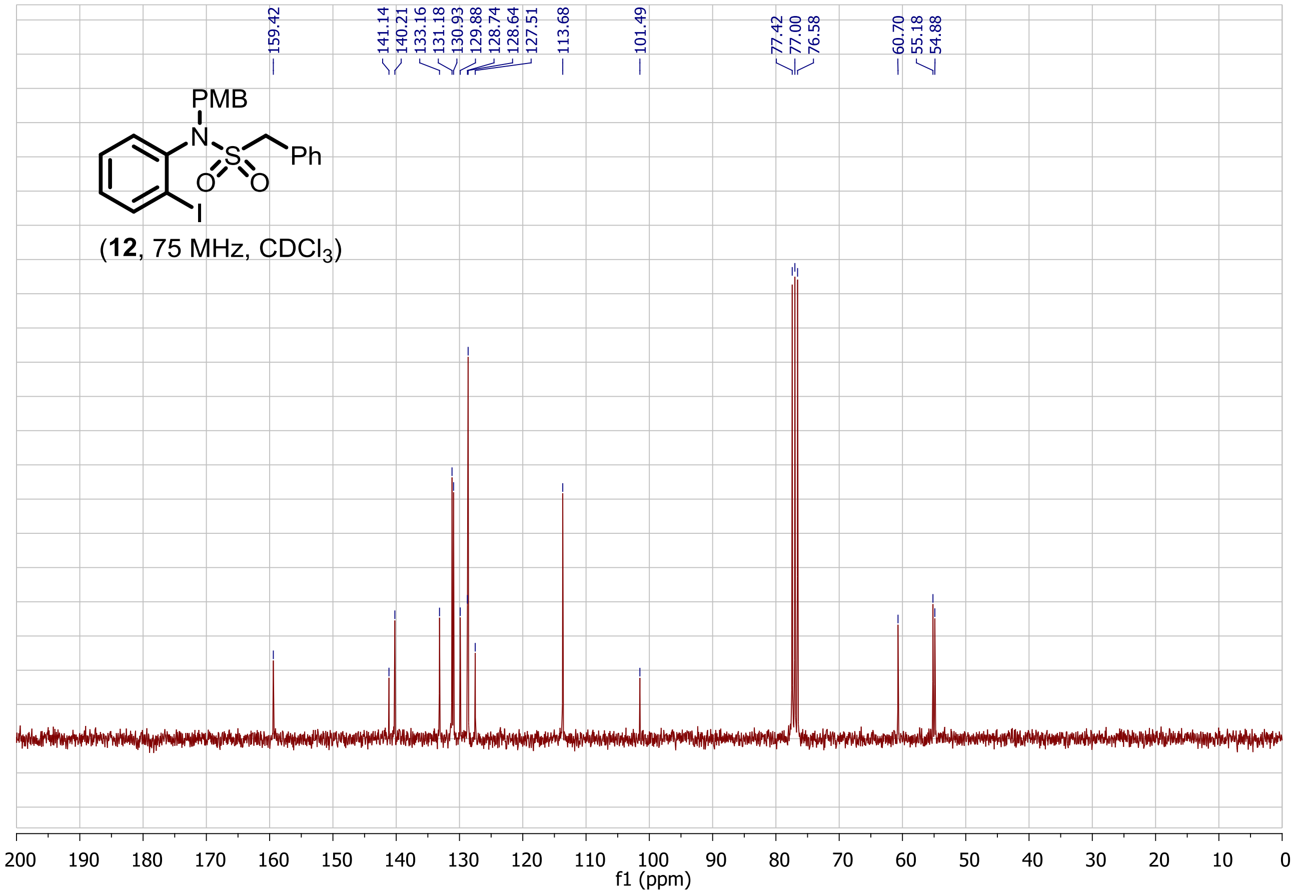
159.42 141.14 140.21 133.16 131.18 130.93 129.88 128.74 128.64 127.51 113.68 101.49 77.42 77.00 76.58 60.70 55.18 54.88
PMB
N
S
O O
Ph
I
(**12**, 75 MHz, $CDCl_3$)
200 190 180 170 160 150 140 130 120 110 100 90 80 70 60 50 40 30 20 10 0
f1 (ppm)

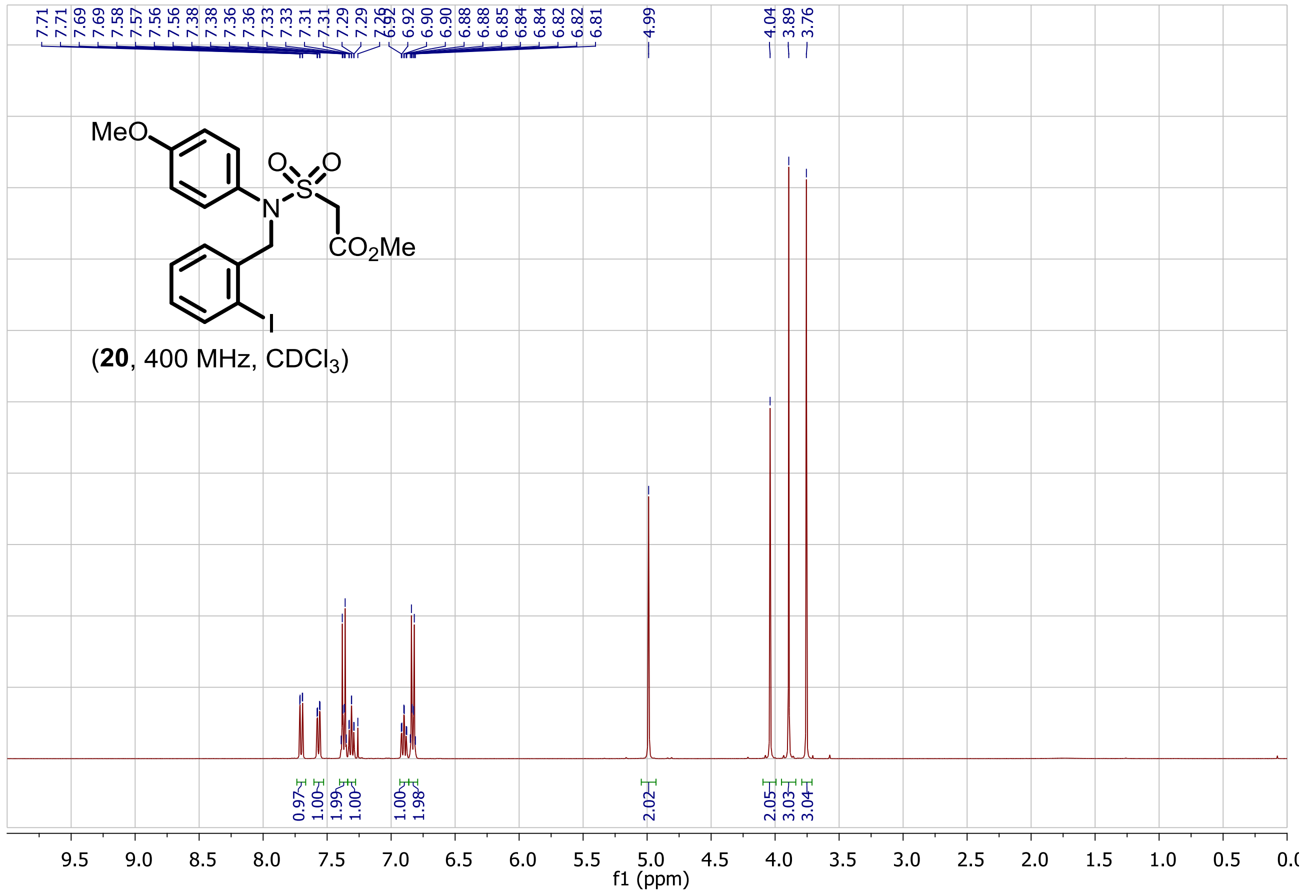
(20, 400 MHz, CDCl3)
f1 (ppm)

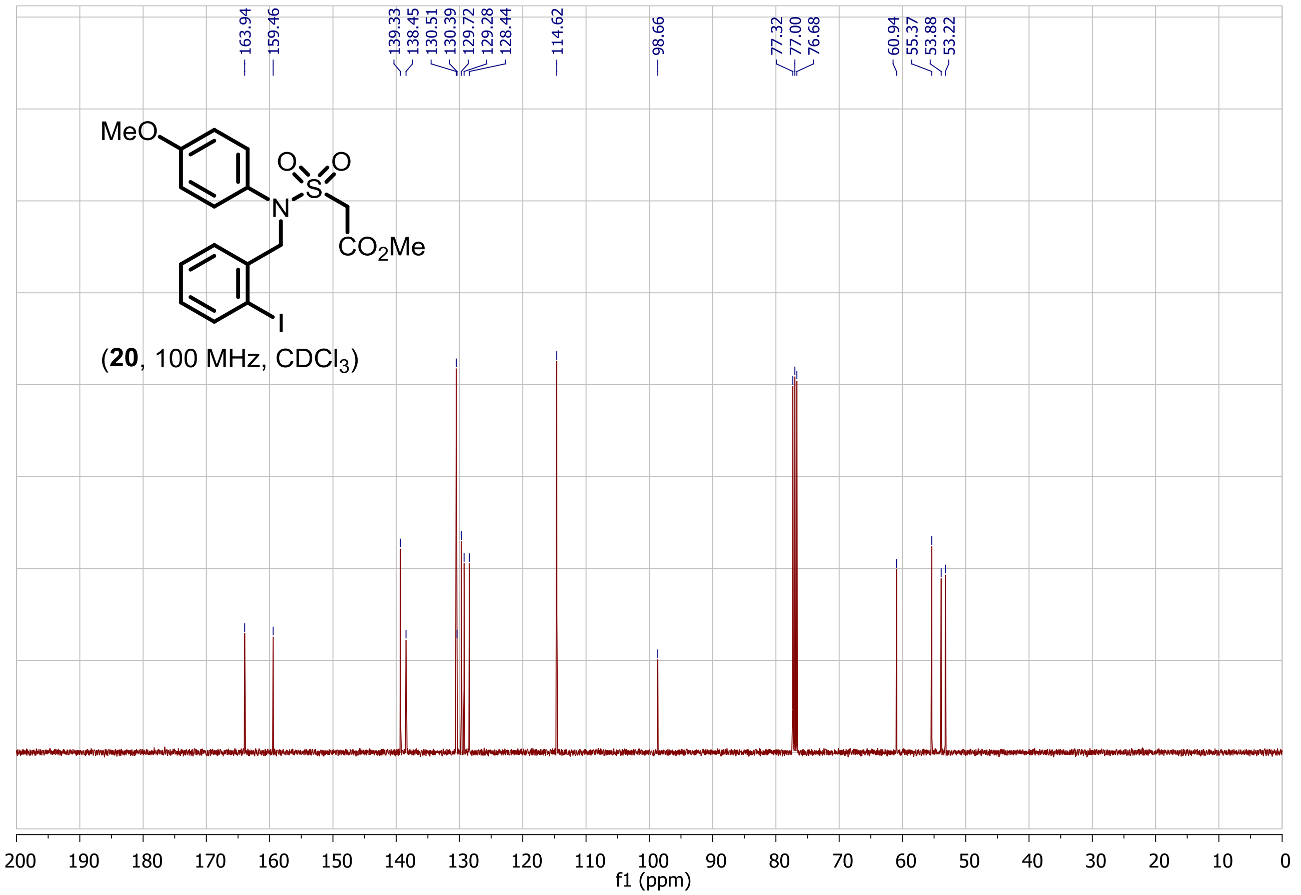
(20, 100 MHz, CDCl3)
f1 (ppm)

3.3. ^{1}H and ^{13}C NMR spectra of sultams

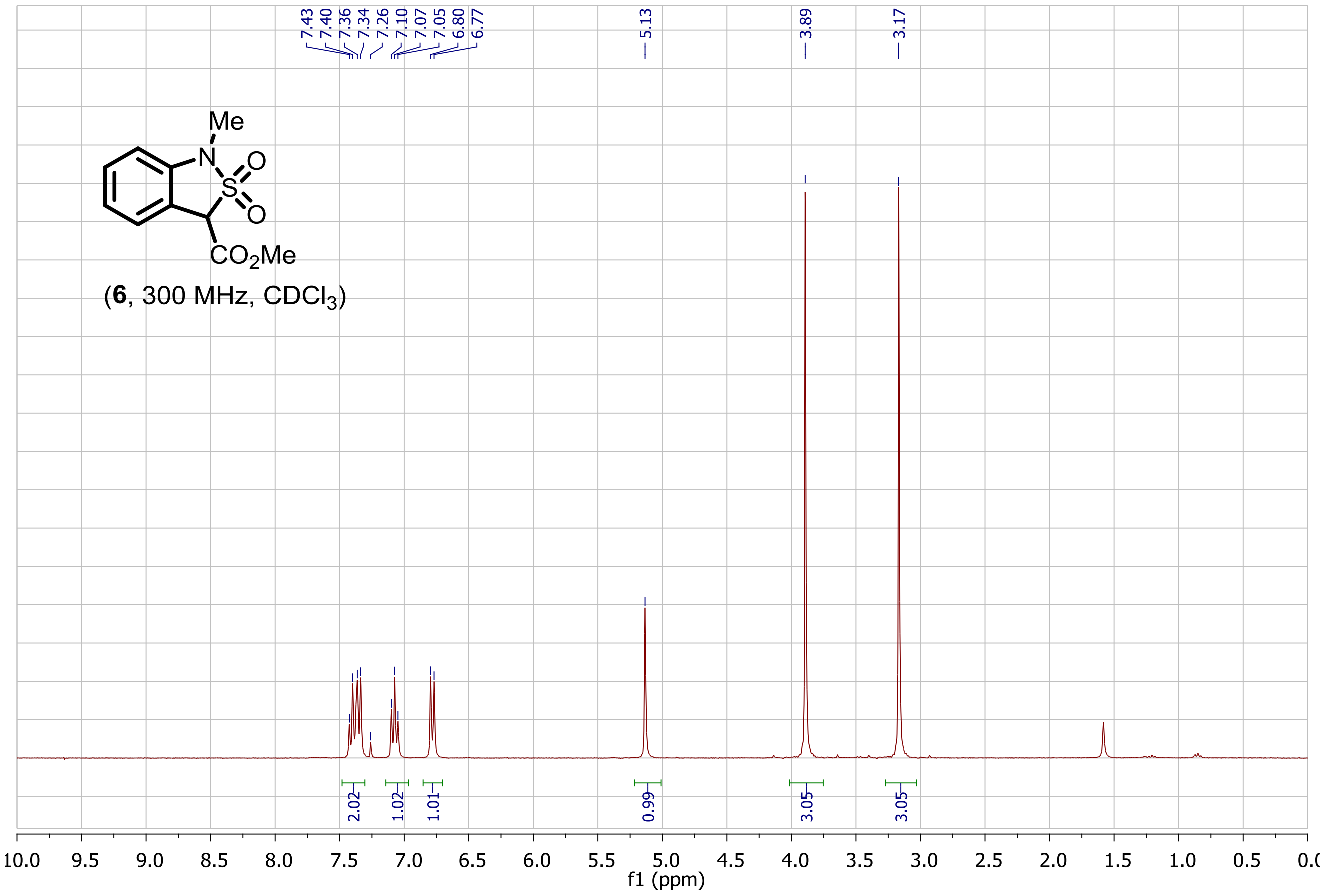

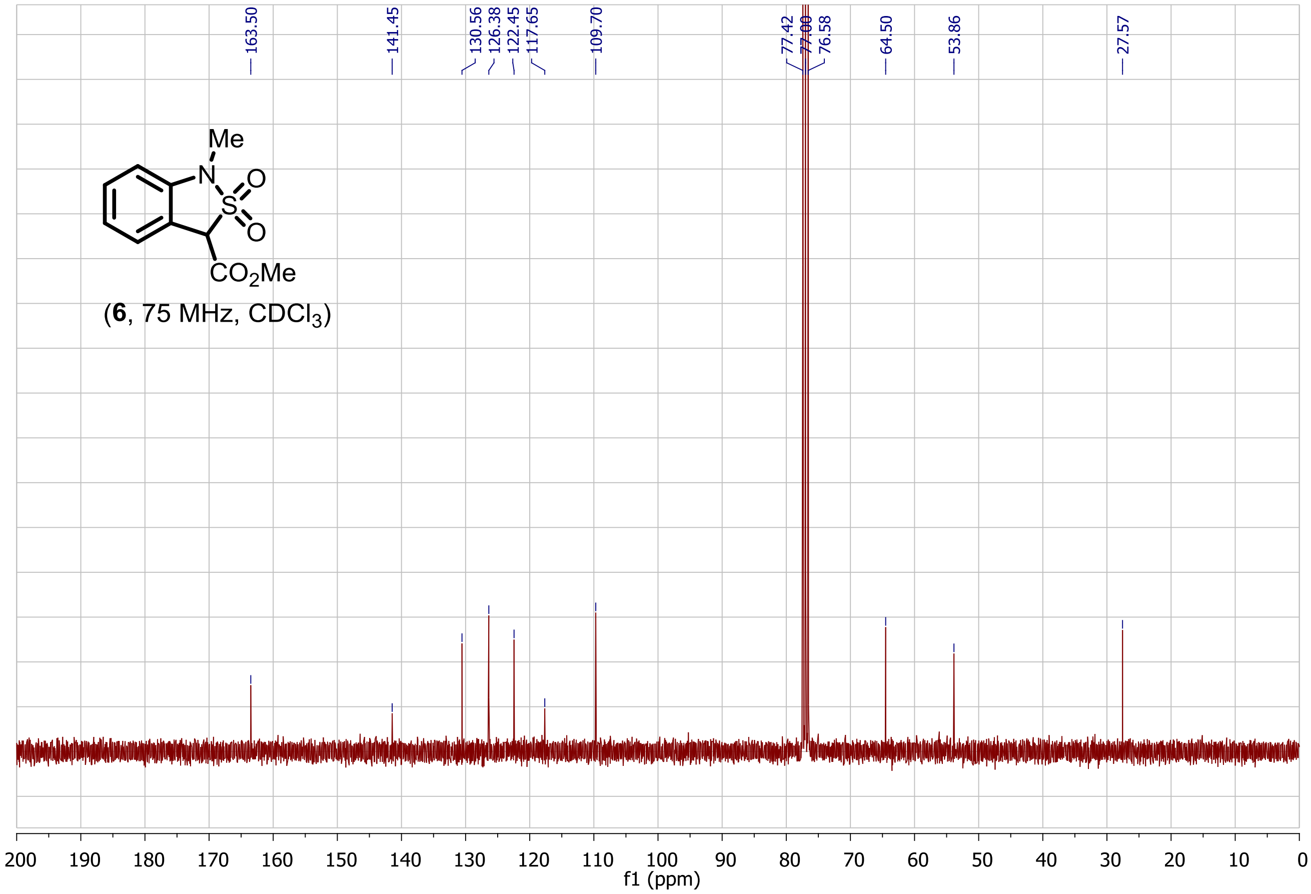

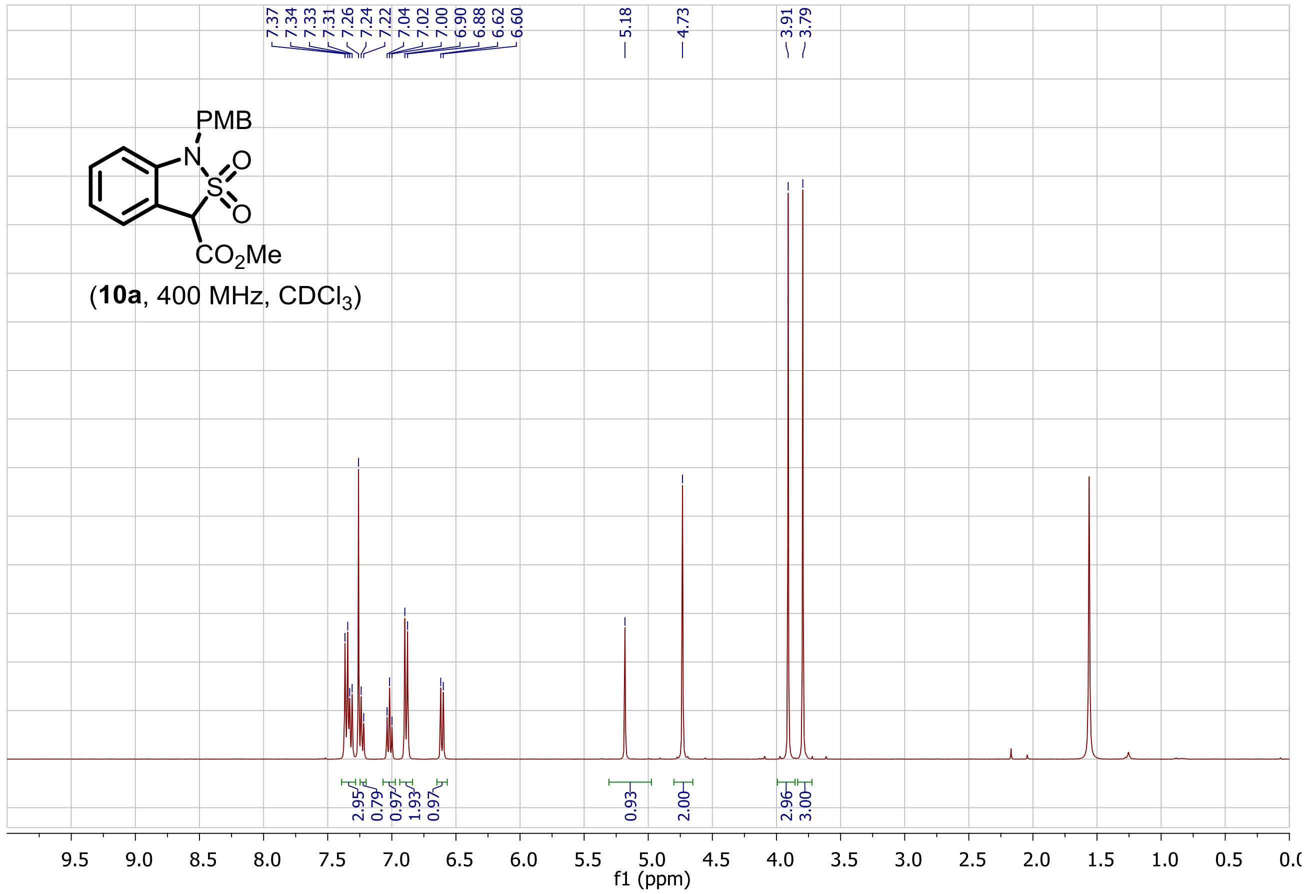
7.37
7.34
7.33
7.31
7.26
7.24
7.22
7.04
7.02
7.00
6.90
6.88
6.62
6.60
5.18
4.73
3.91
3.79
PMB
N
S
O
O
CO2Me
(10a, 400 MHz, CDCl3)
2.95
0.79
0.97
1.93
0.97
0.93
2.00
2.96
3.00
9.5
9.0
8.5
8.0
7.5
7.0
6.5
6.0
5.5
5.0
4.5
4.0
3.5
3.0
2.5
2.0
1.5
1.0
0.5
f1 (ppm)

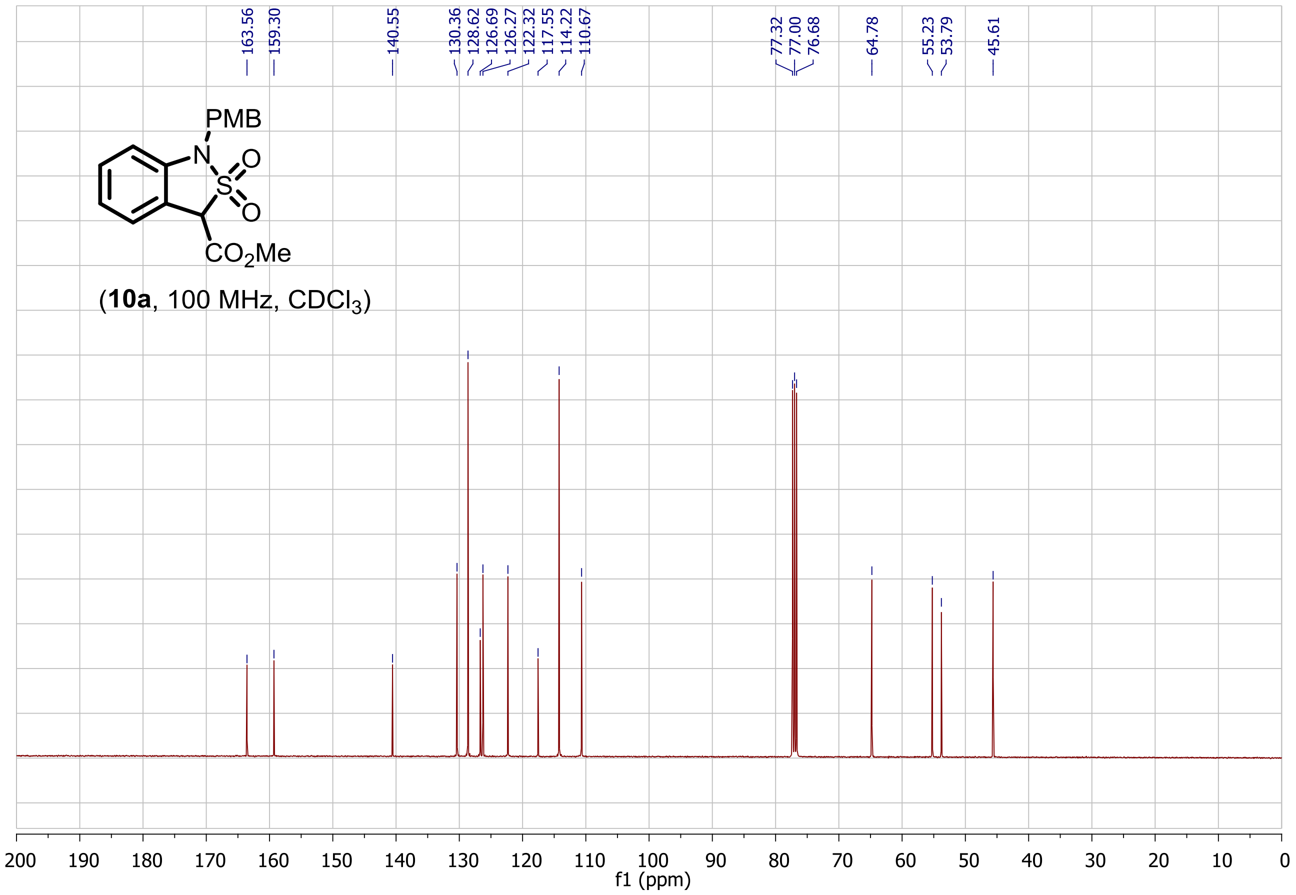
163.56
159.30
140.55
130.36
128.62
126.69
126.27
122.32
117.55
114.22
110.67
77.32
77.00
76.68
64.78
55.23
53.79
45.61
PMB
N
S
O
O
CO2Me
(10a, 100 MHz, CDCl3)
200
190
180
170
160
150
140
130
120
110
100
90
80
70
60
50
40
30
20
10
0
f1 (ppm)

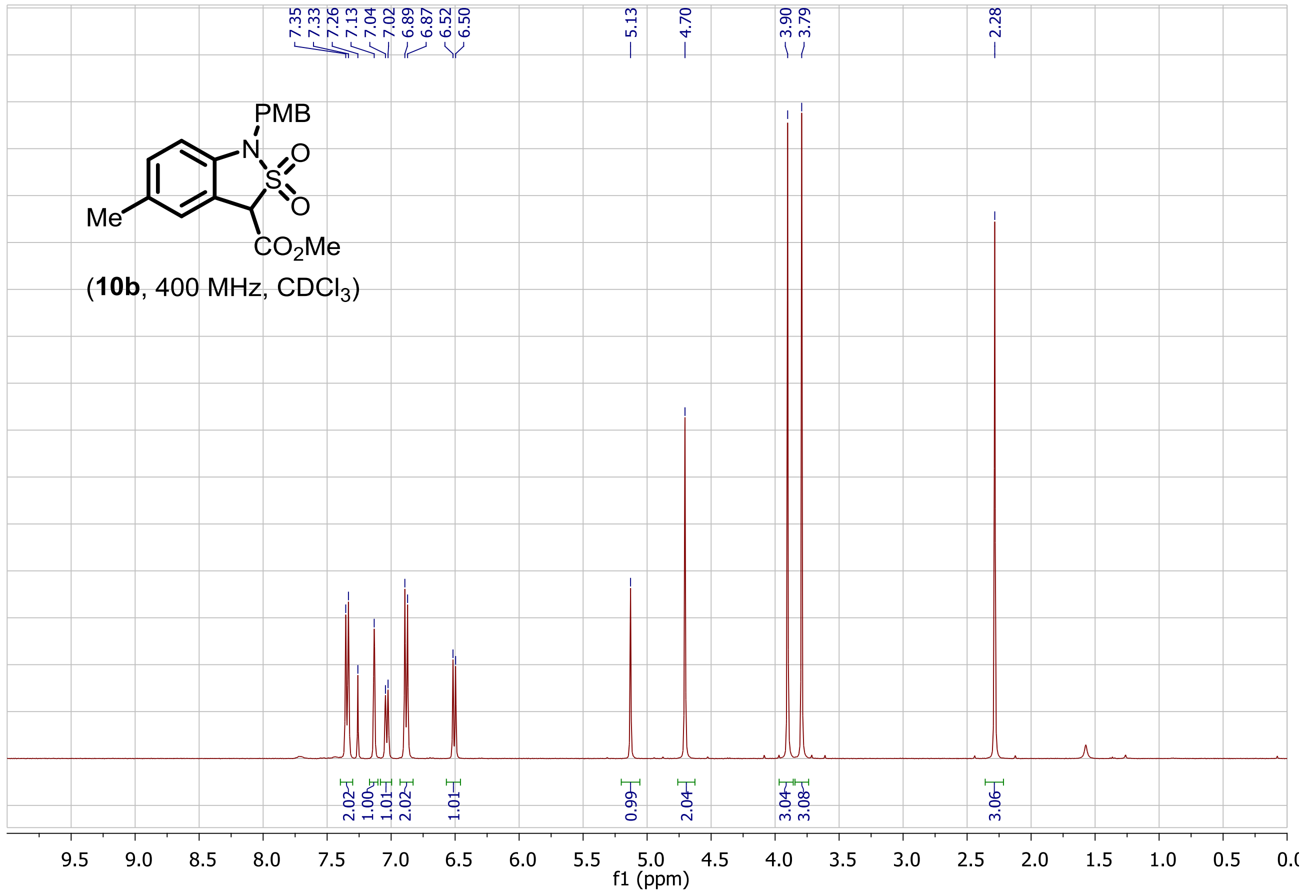

7.35
7.33
7.26
7.13
7.04
7.02
6.89
6.87
6.52
6.50
5.13
4.70
3.90
3.79
2.28
PMB
N
S
O
O
Me
CO2Me
(10b, 400 MHz, CDCl3)
2.02
1.00
1.01
2.02
1.01
0.99
2.04
3.04
3.08
3.06
9.5
9.0
8.5
8.0
7.5
7.0
6.5
6.0
5.5
5.0
4.5
4.0
3.5
3.0
2.5
2.0
1.5
1.0
0.5
f1 (ppm)

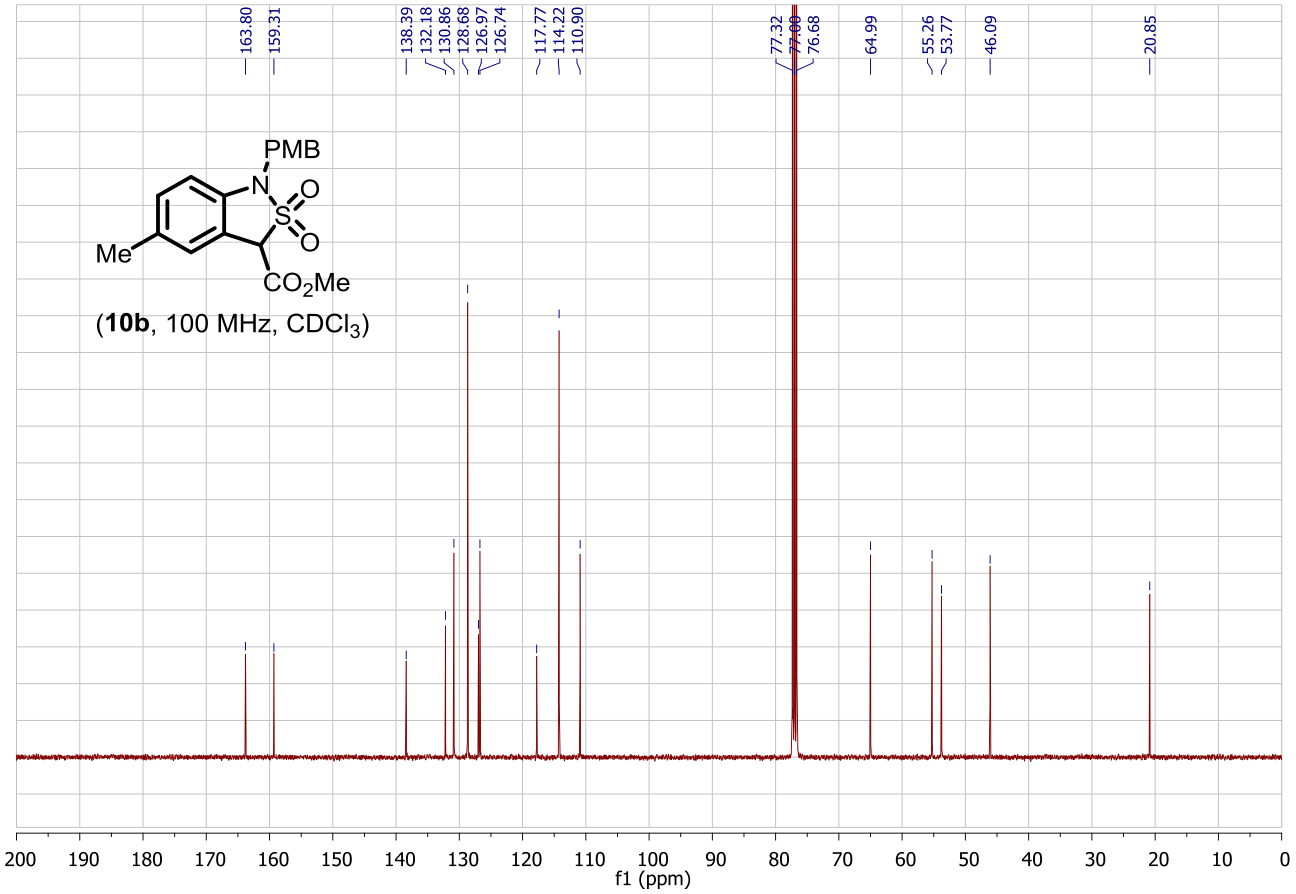

163.80
159.31
138.39
132.18
130.86
128.68
126.97
126.74
117.77
114.22
110.90
77.32
77.00
76.68
64.99
55.26
53.77
46.09
20.85
PMB
N
S
O
O
Me
CO2Me
(10b, 100 MHz, CDCl3)
200
190
180
170
160
150
140
130
120
110
100
90
80
70
60
50
40
30
20
10
0
f1 (ppm)

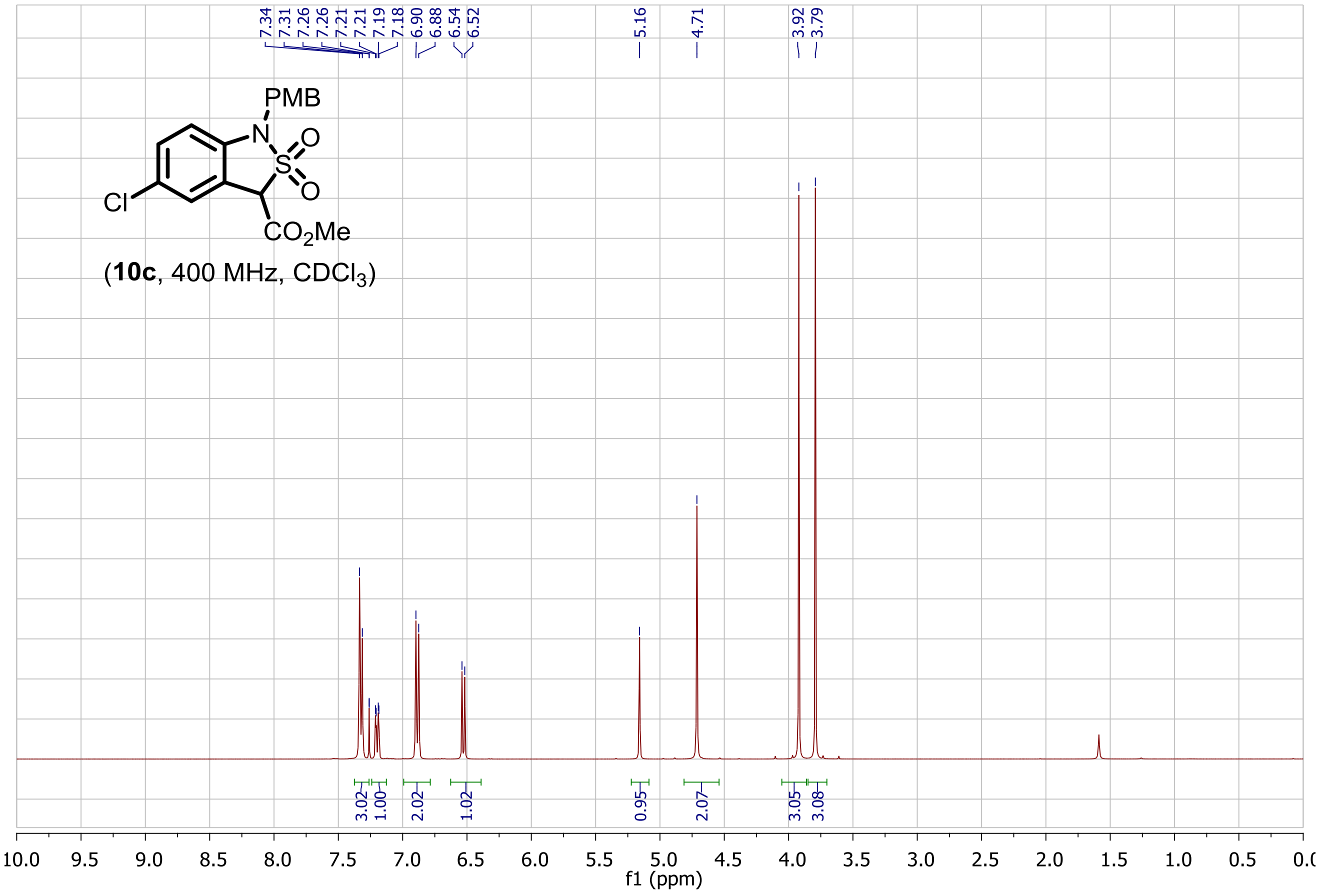
7.34
7.31
7.26
7.26
7.21
7.21
7.19
7.18
6.90
6.88
6.54
6.52
5.16
4.71
3.92
3.79
PMB
N
S
O
O
Cl
CO2Me
(10c, 400 MHz, CDCl3)
3.02
1.00
2.02
1.02
0.95
2.07
3.05
3.08
f1 (ppm)

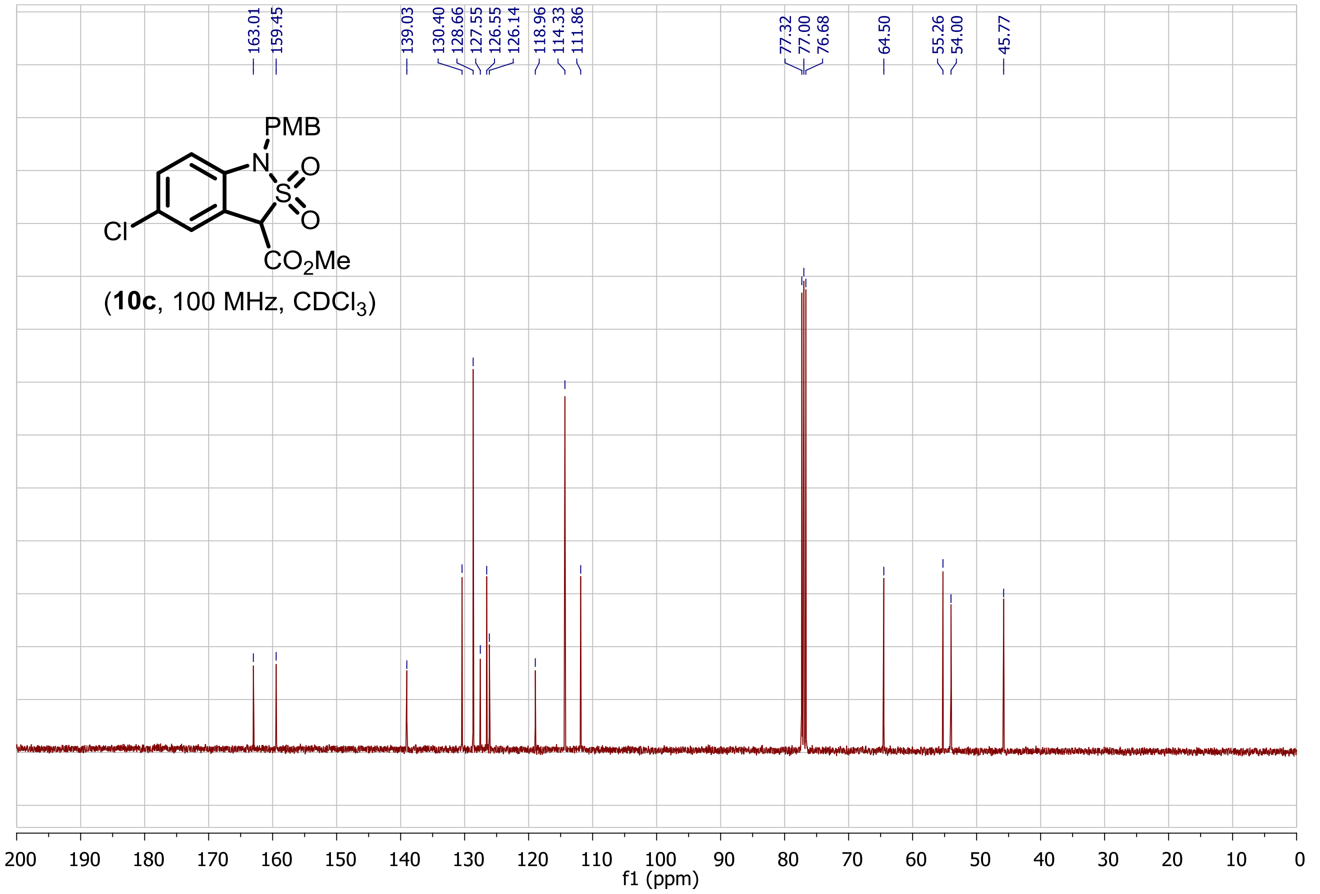
163.01
159.45
139.03
130.40
128.66
127.55
126.55
126.14
118.96
114.33
111.86
77.32
77.00
76.68
64.50
55.26
54.00
45.77
PMB
N
S
O
O
Cl
CO2Me
(10c, 100 MHz, CDCl3)
f1 (ppm)

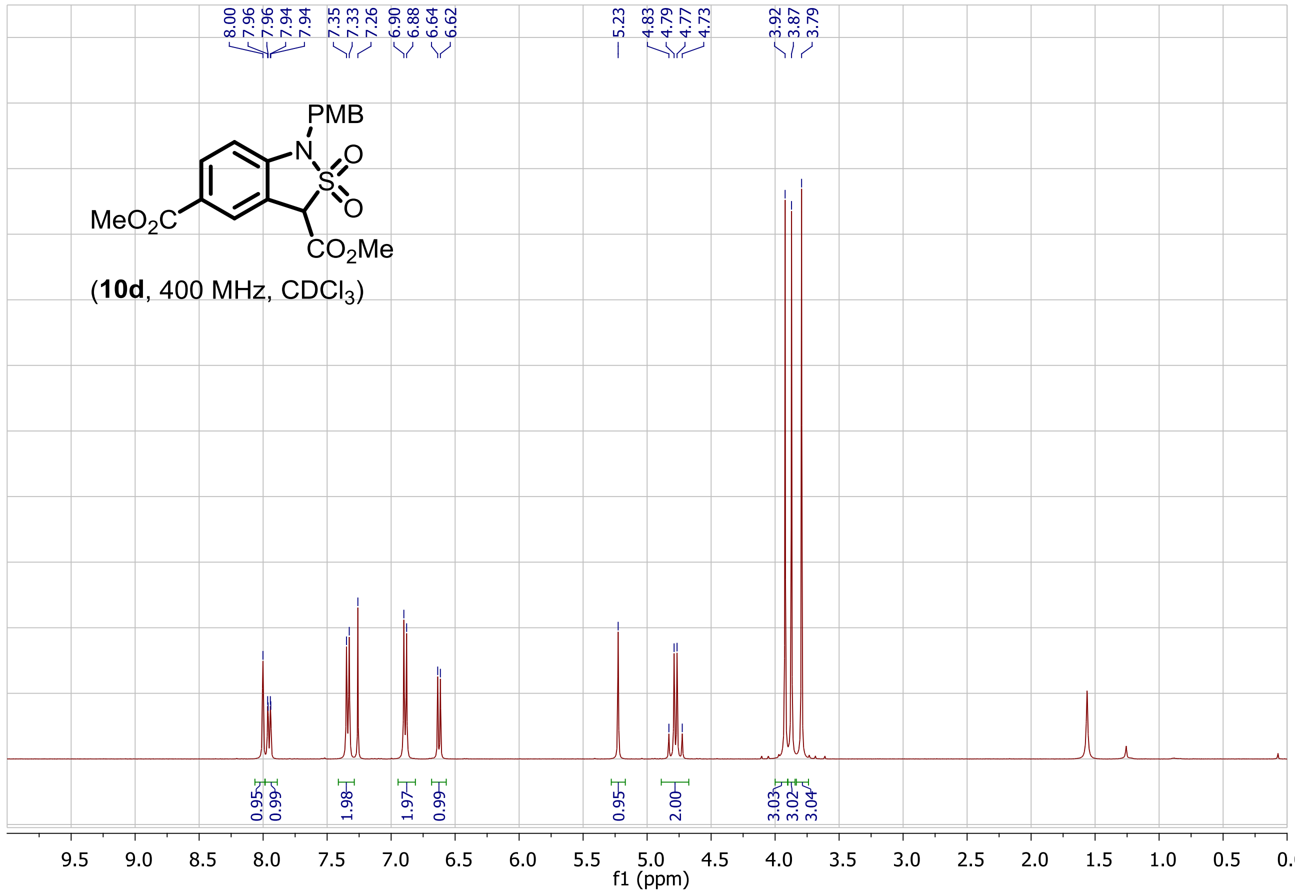
8.00 7.96 7.96 7.94 7.94 7.35 7.33 7.26 6.90 6.88 6.64 6.62
5.23 4.83 4.79 4.77 4.73
3.92 3.87 3.79
PMB
N
S
O
O
MeO2C
CO2Me
(10d, 400 MHz, CDCl3)
0.95 0.99 1.98 1.97 0.99 0.95 2.00 3.03 3.02 3.04
f1 (ppm)

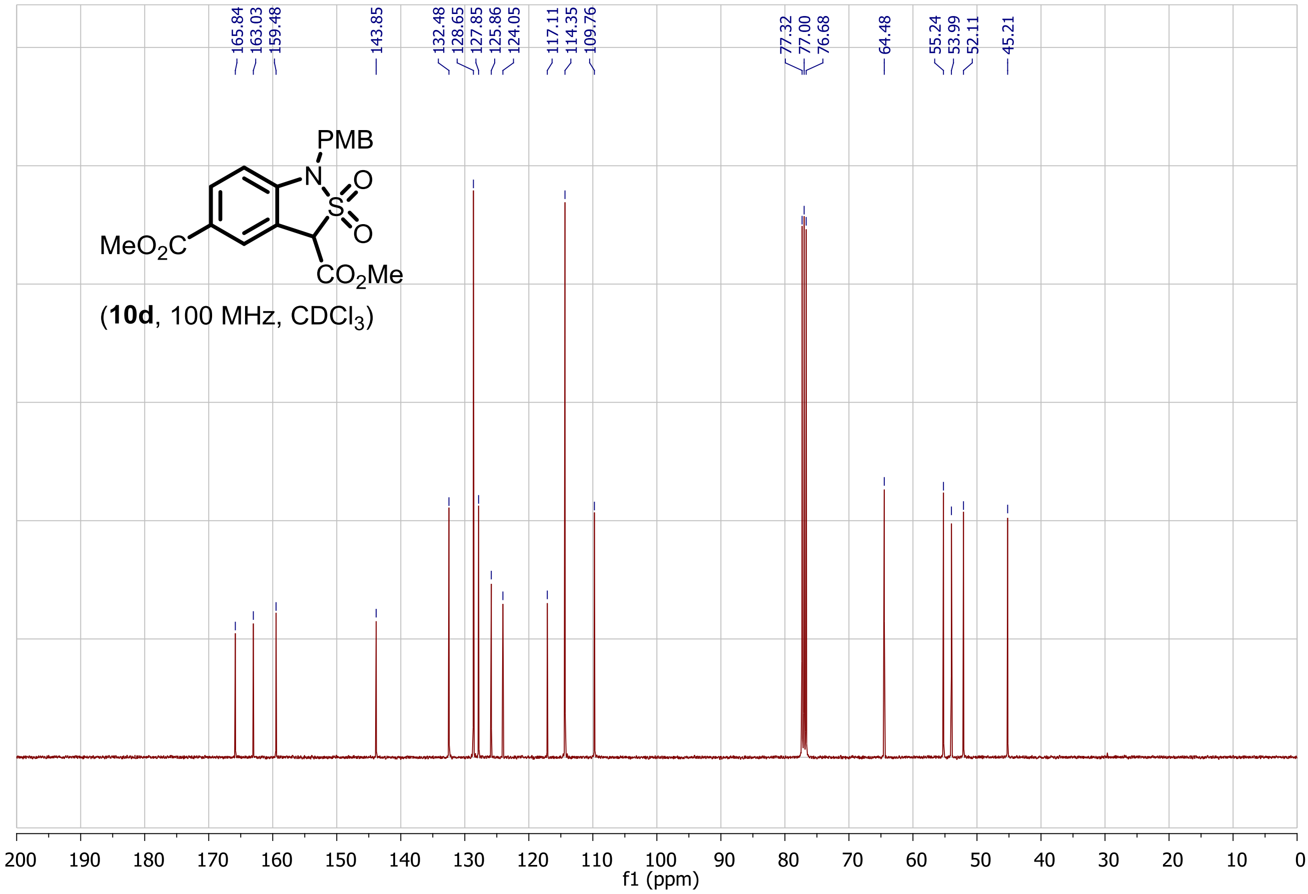
165.84 163.03 159.48 143.85 132.48 128.65 127.85 125.86 124.05 117.11 114.35 109.76
77.32 77.00 76.68 64.48 55.24 53.99 52.11 45.21
PMB
N
S
O
O
MeO2C
CO2Me
(10d, 100 MHz, CDCl3)
f1 (ppm)

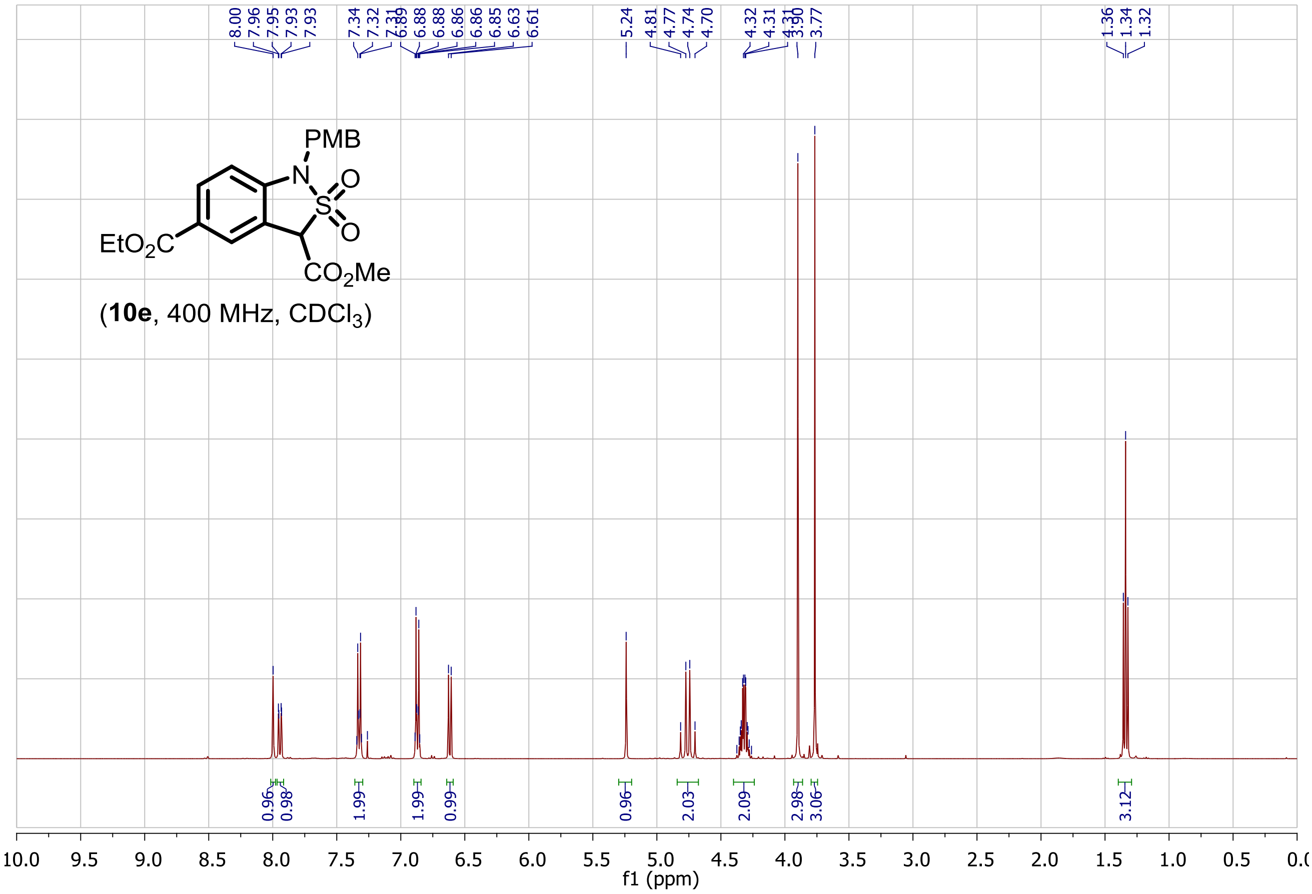
PMB
EtO2C
CO2Me
(10e, 400 MHz, CDCl3)
f1 (ppm)

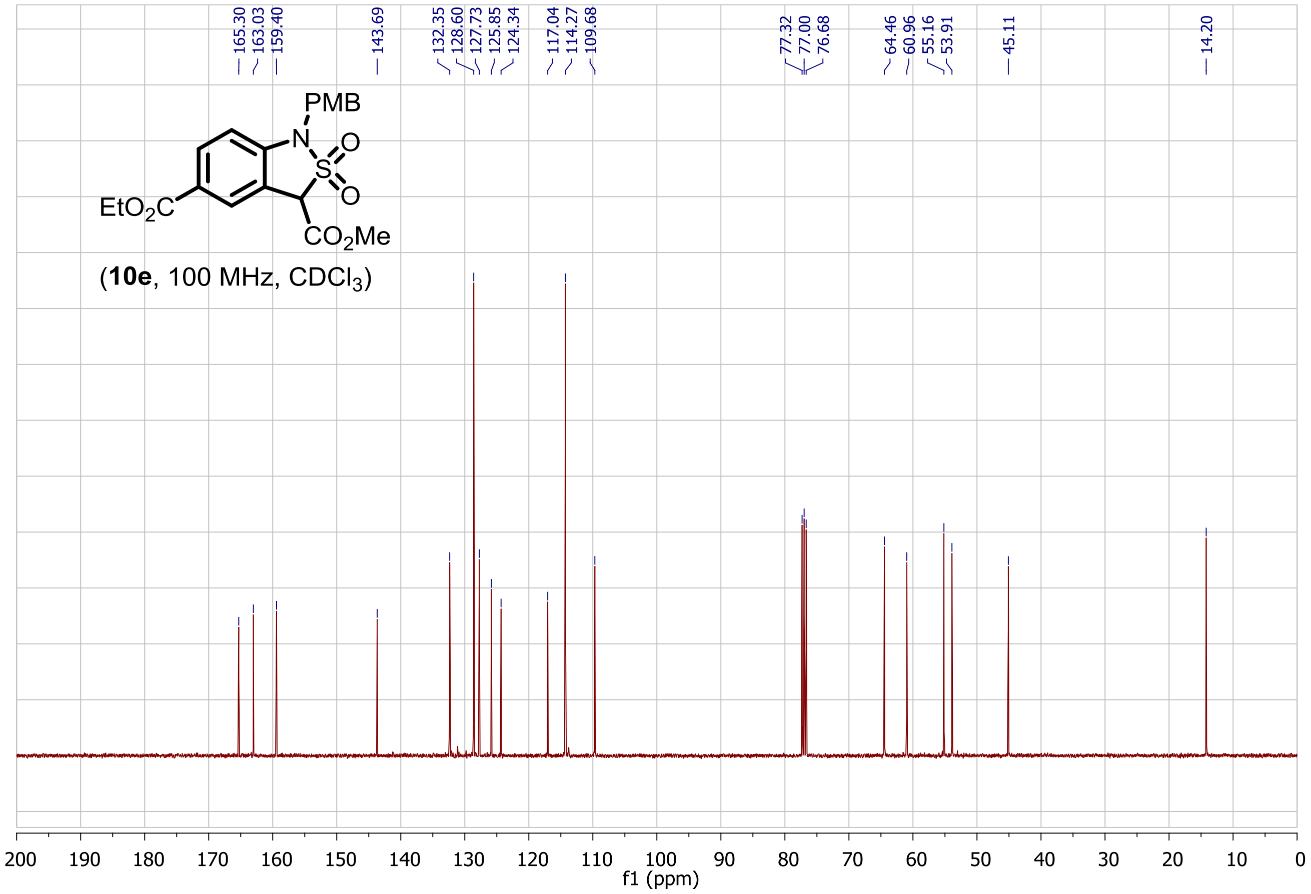
PMB
EtO2C
CO2Me
(10e, 100 MHz, CDCl3)
f1 (ppm)

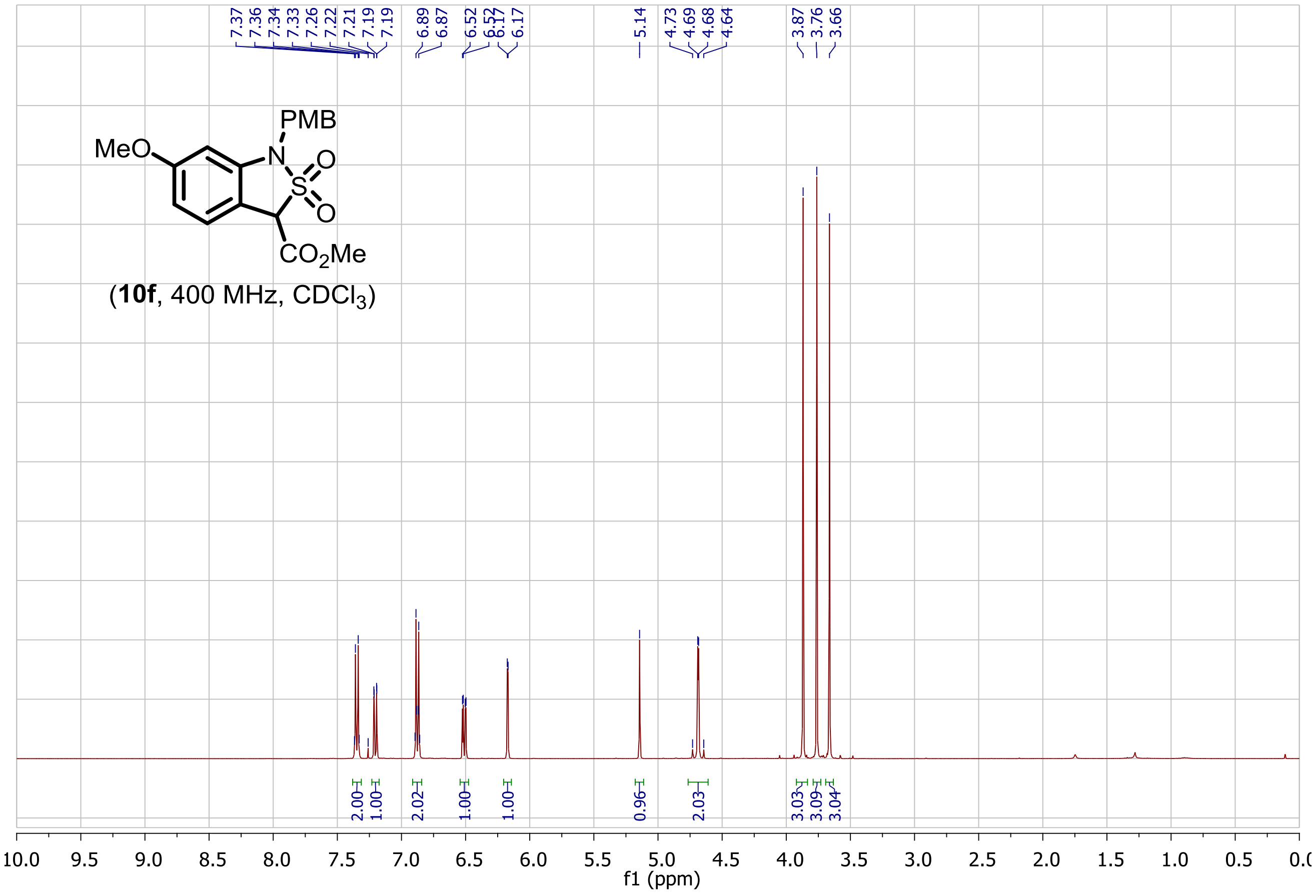

7.37
7.36
7.34
7.33
7.26
7.22
7.21
7.19
7.19
6.89
6.87
6.52
6.52
6.17
6.17
5.14
4.73
4.69
4.68
4.64
3.87
3.76
3.66
PMB
MeO
N
S
O
O
CO2Me
(10f, 400 MHz, CDCl3)
2.00
1.00
2.02
1.00
1.00
0.96
2.03
3.03
3.09
3.04
f1 (ppm)

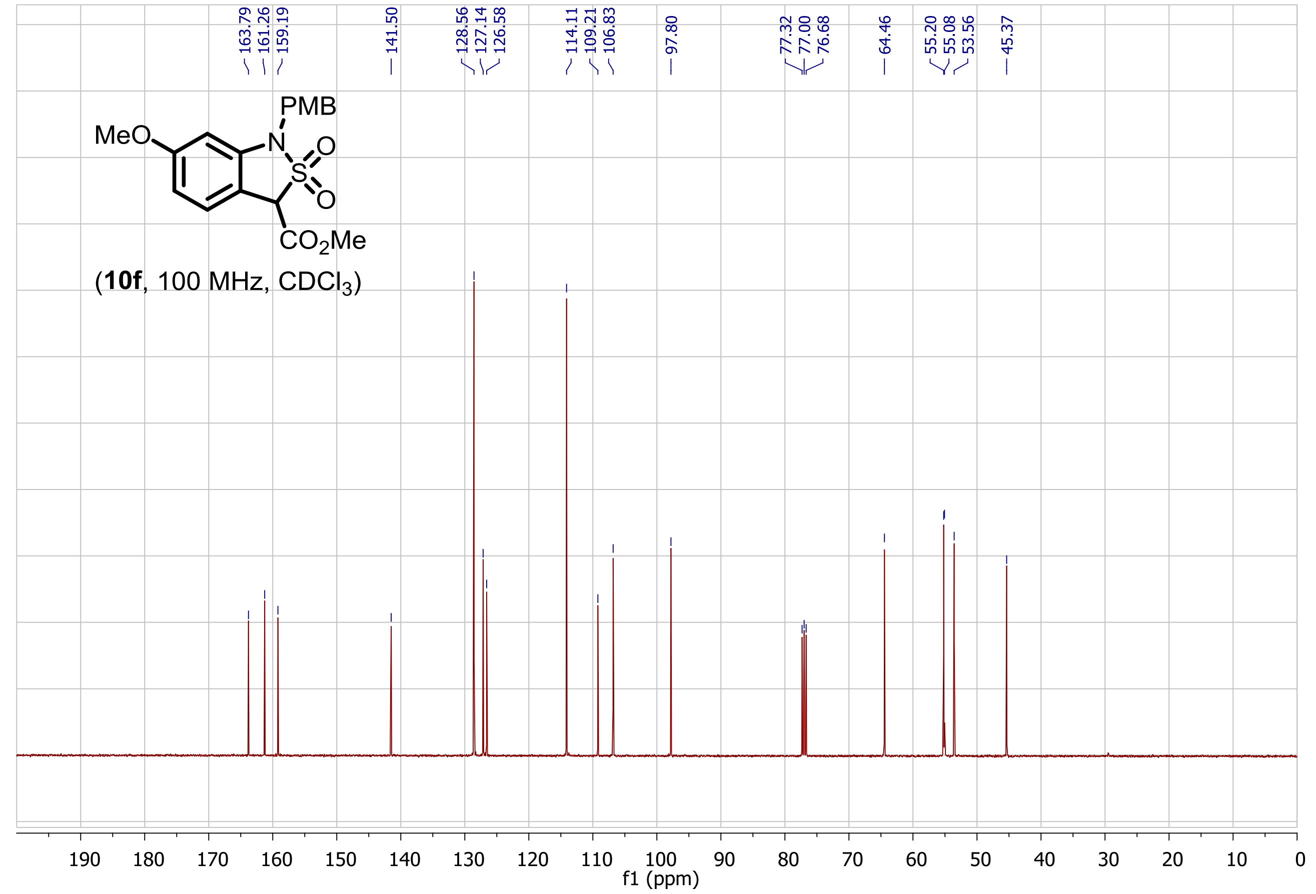

163.79
161.26
159.19
141.50
128.56
127.14
126.58
114.11
109.21
106.83
97.80
77.32
77.00
76.68
64.46
55.20
55.08
53.56
45.37
PMB
MeO
N
S
O
O
CO2Me
(10f, 100 MHz, CDCl3)
f1 (ppm)

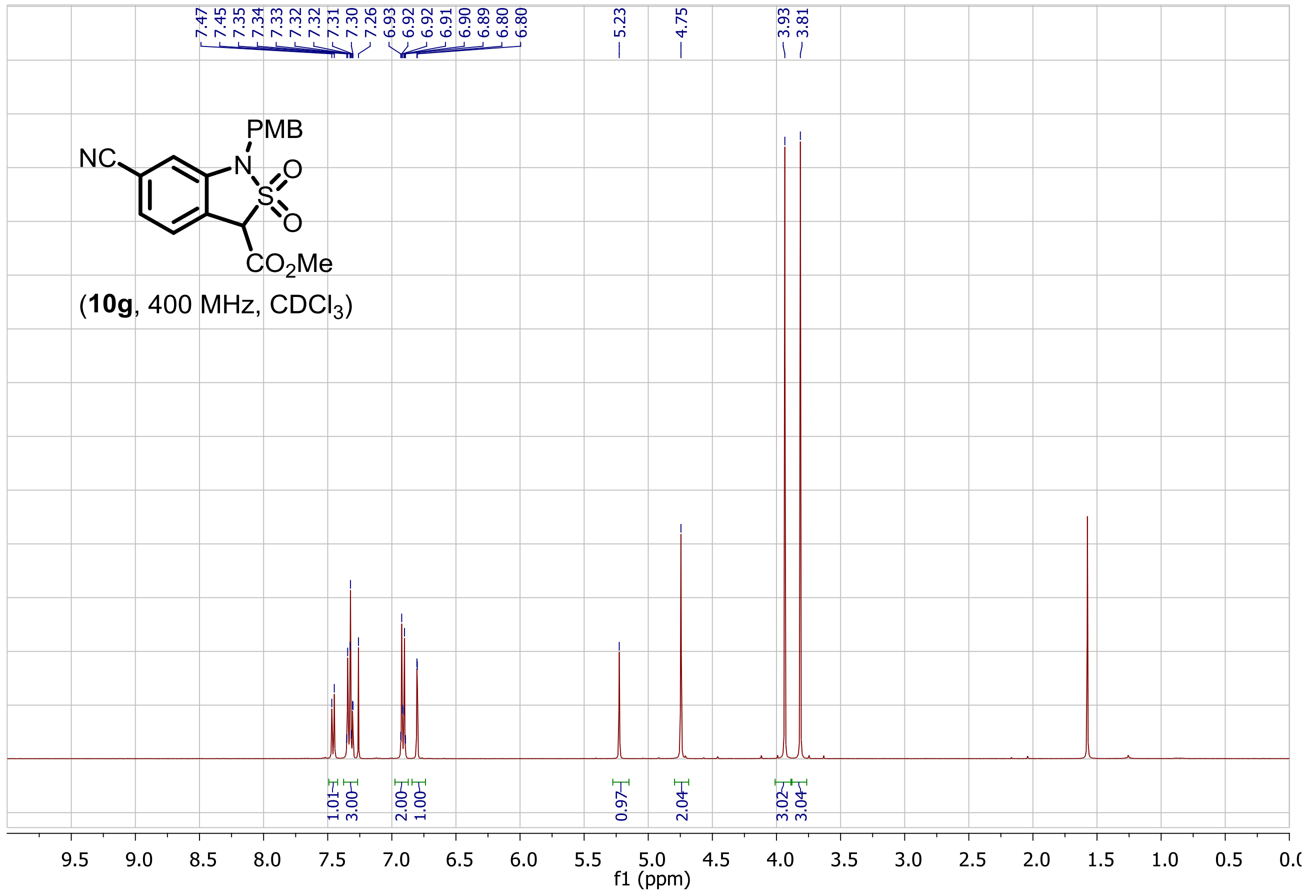
7.47
7.45
7.35
7.34
7.33
7.32
7.32
7.31
7.30
7.26
6.93
6.92
6.92
6.91
6.90
6.89
6.80
6.80
5.23
4.75
3.93
3.81
PMB
NC
N
S
O
O
CO_2Me
(**10g**, 400 MHz, $CDCl_3$)
1.01
3.00
2.00
1.00
0.97
2.04
3.02
3.04
f1 (ppm)

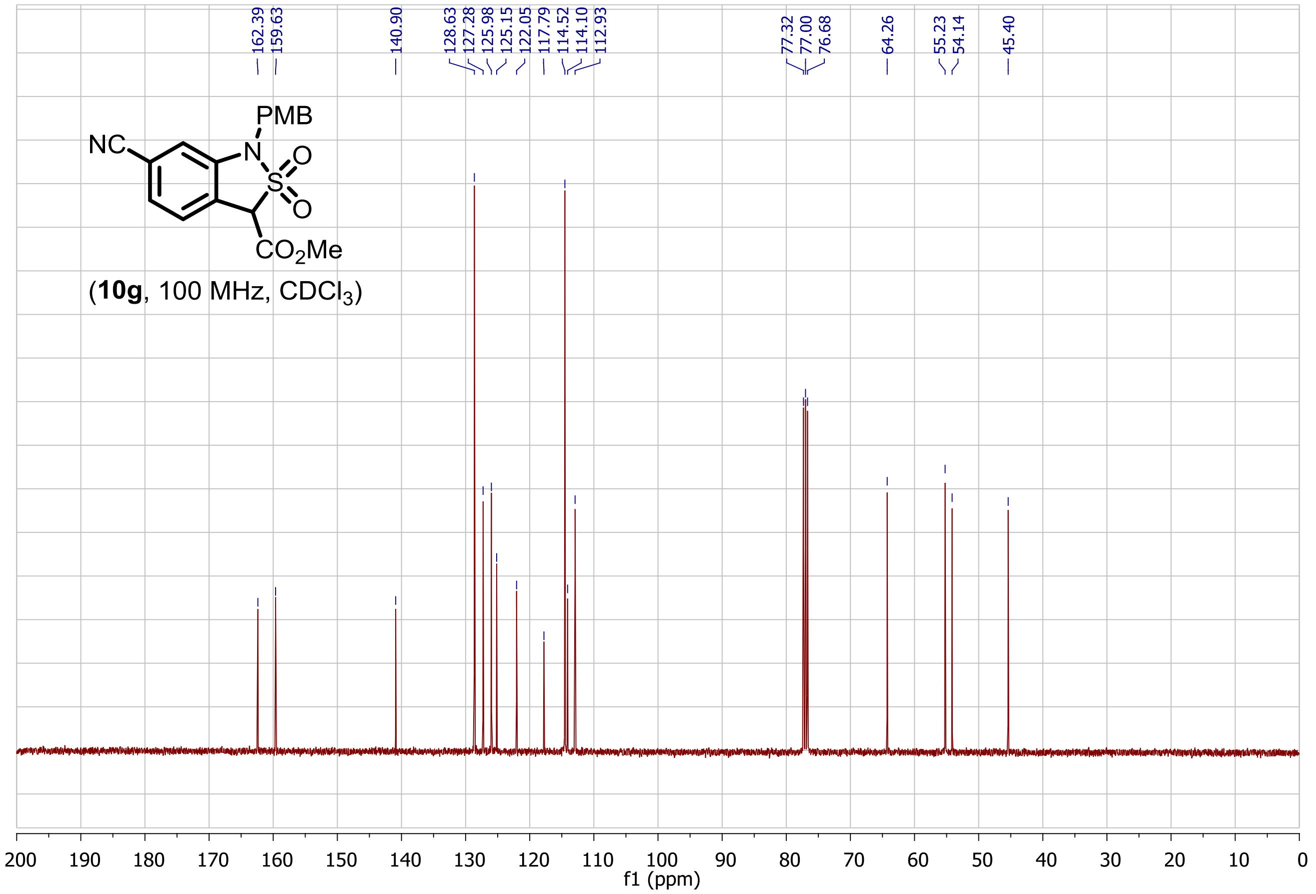
162.39
159.63
140.90
128.63
127.28
125.98
125.15
122.05
117.79
114.52
114.10
112.93
77.32
77.00
76.68
64.26
55.23
54.14
45.40
PMB
NC
N
S
O
O
CO_2Me
(**10g**, 100 MHz, $CDCl_3$)
f1 (ppm)

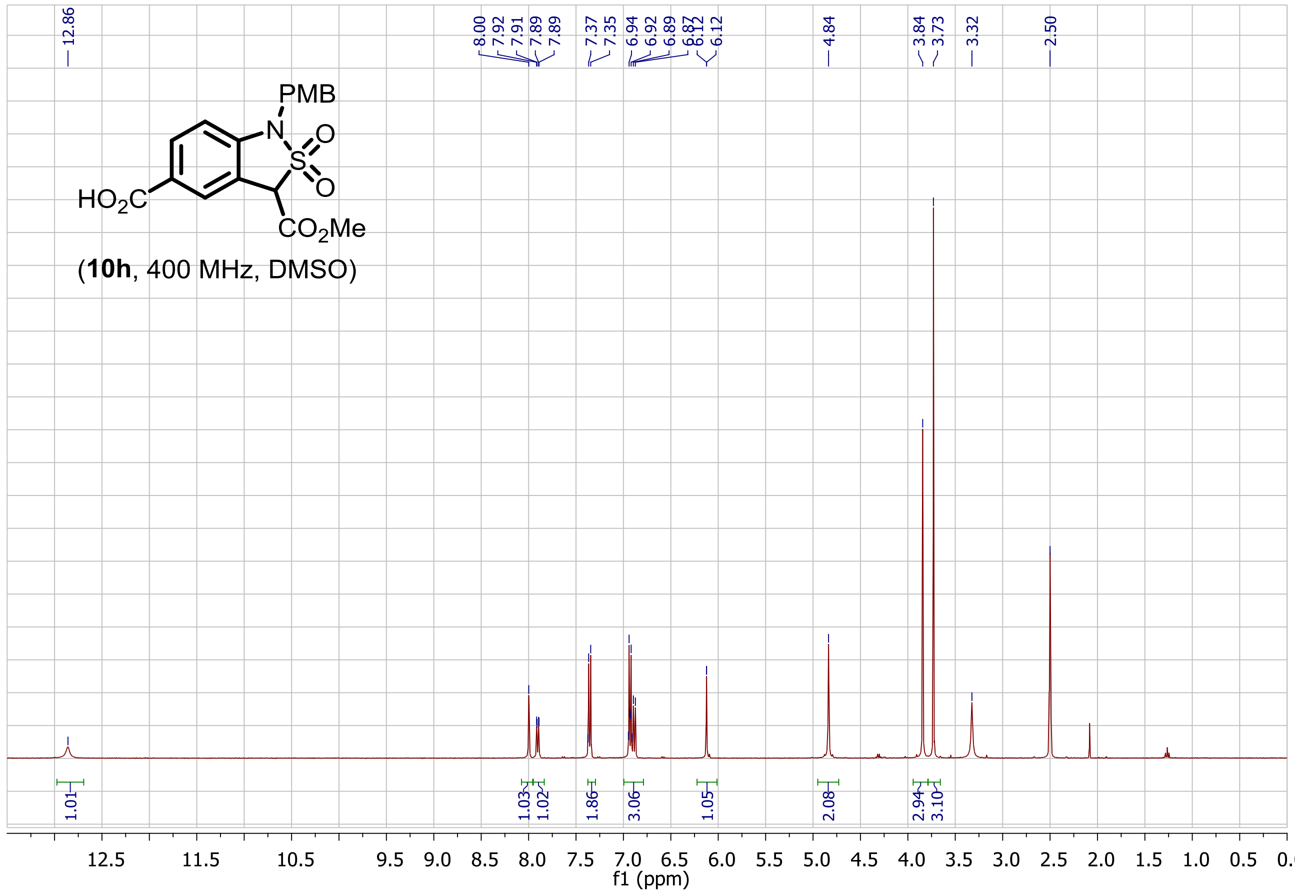
PMB
N
S
O
O
HO_2C
CO_2Me
(**10h**, 400 MHz, DMSO)
f1 (ppm)

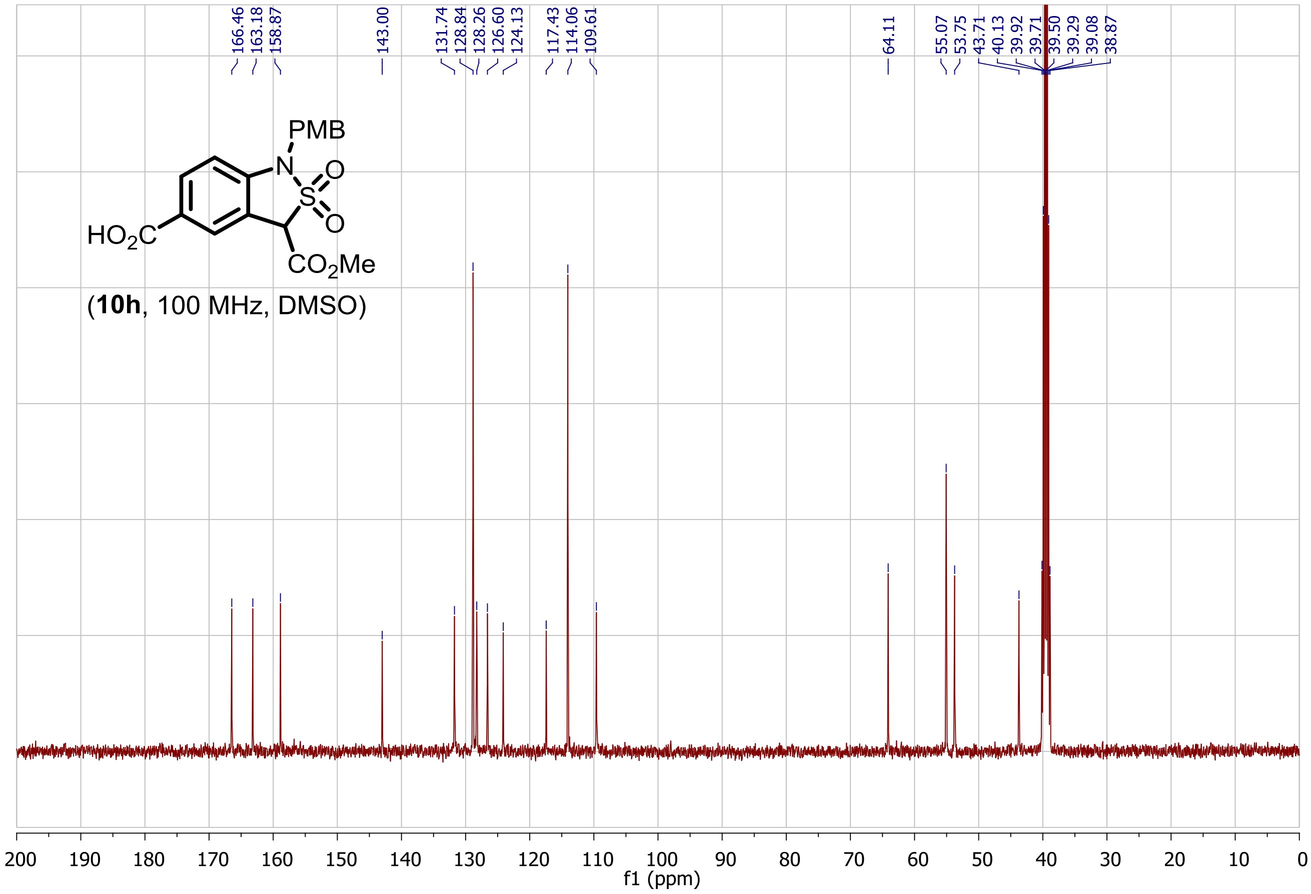
PMB
N
S
O
O
HO_2C
CO_2Me
(**10h**, 100 MHz, DMSO)
f1 (ppm)

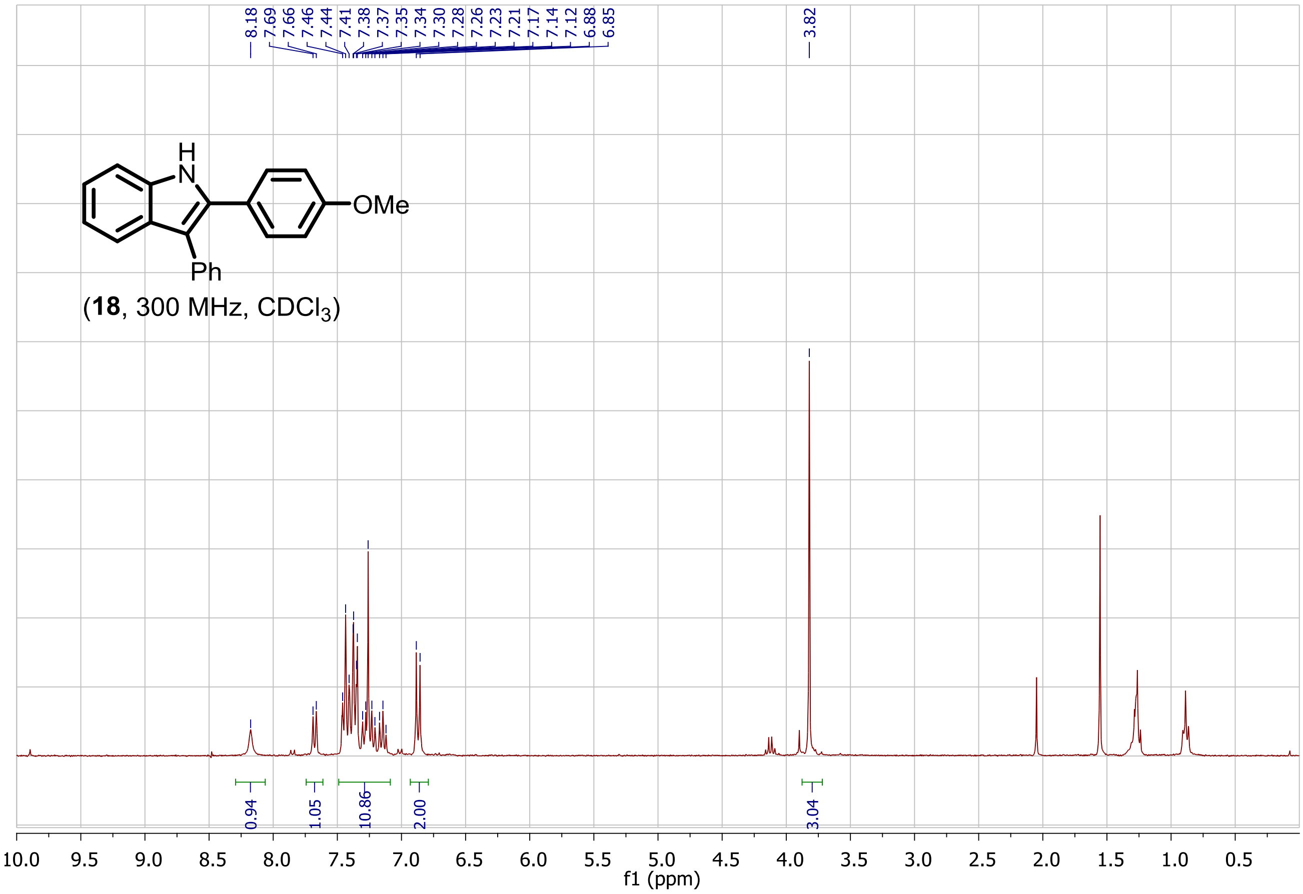
8.18
7.69
7.66
7.46
7.44
7.41
7.38
7.37
7.35
7.34
7.30
7.28
7.26
7.23
7.21
7.17
7.14
7.12
6.88
6.85
3.82
H
N
OMe
Ph
(18, 300 MHz, CDCl3)
0.94
1.05
10.86
2.00
3.04
10.0 9.5 9.0 8.5 8.0 7.5 7.0 6.5 6.0 5.5 5.0 4.5 4.0 3.5 3.0 2.5 2.0 1.5 1.0 0.5
f1 (ppm)

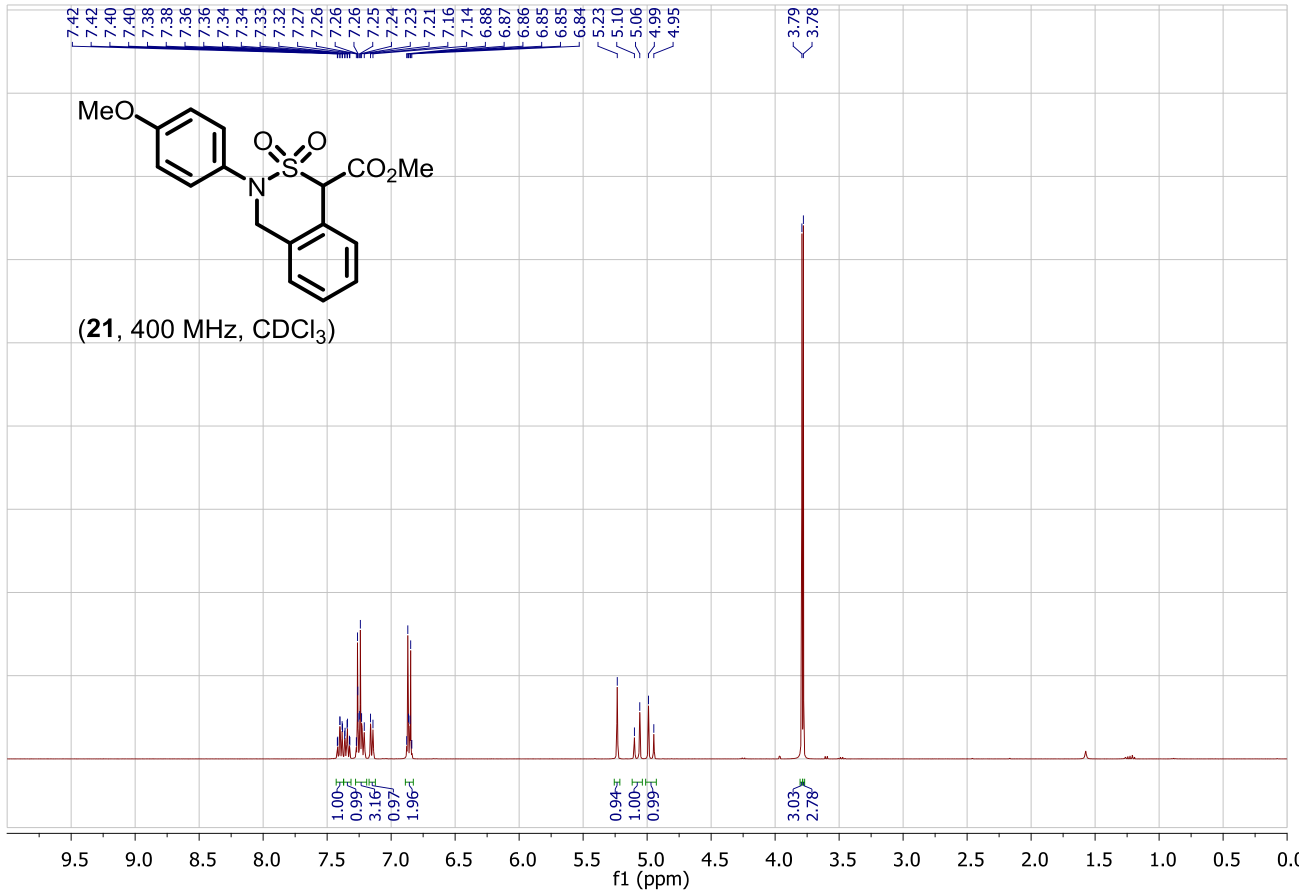
MeO
CO$_2$Me
(**21**, 400 MHz, CDCl$_3$)
f1 (ppm)

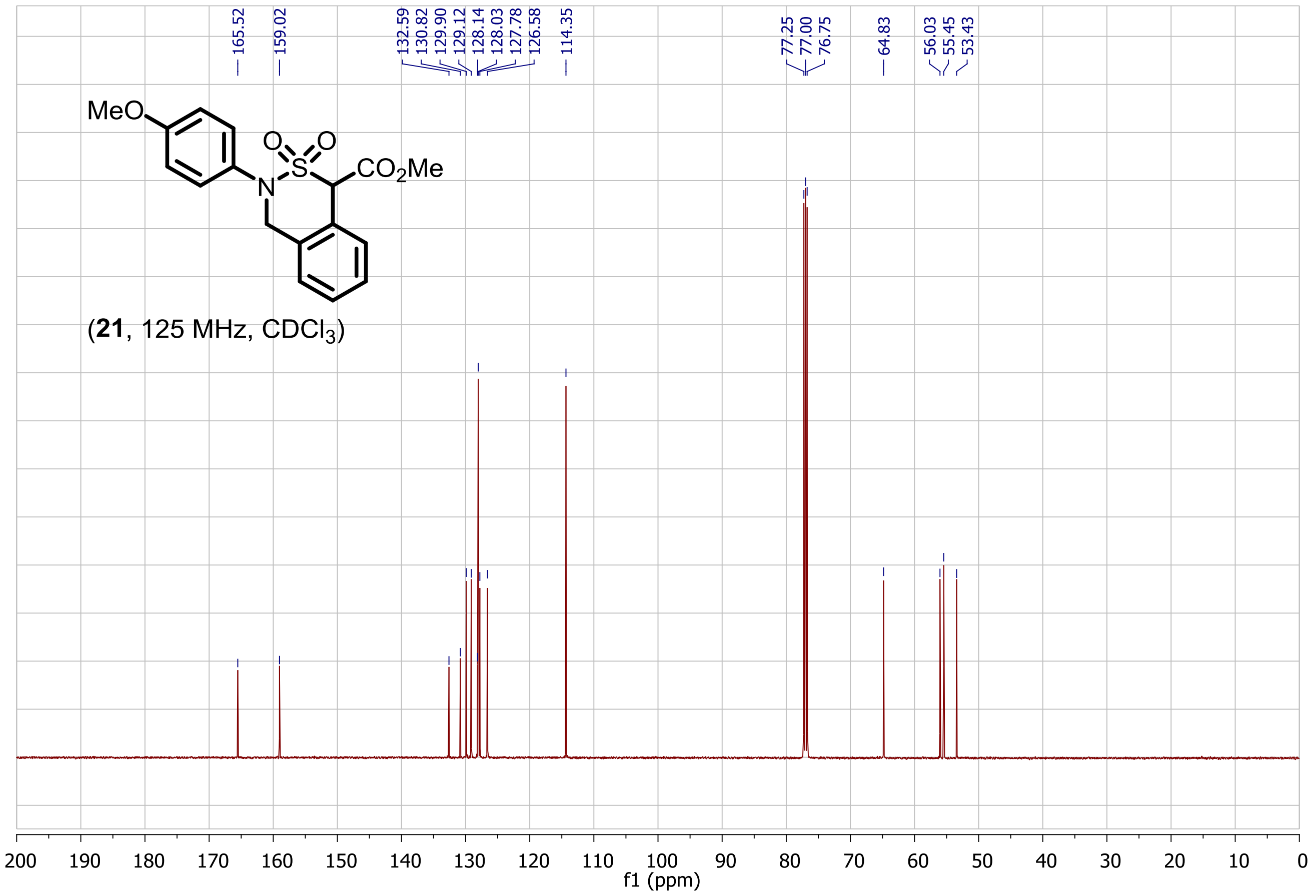
MeO
CO$_2$Me
(**21**, 125 MHz, CDCl$_3$)
f1 (ppm)

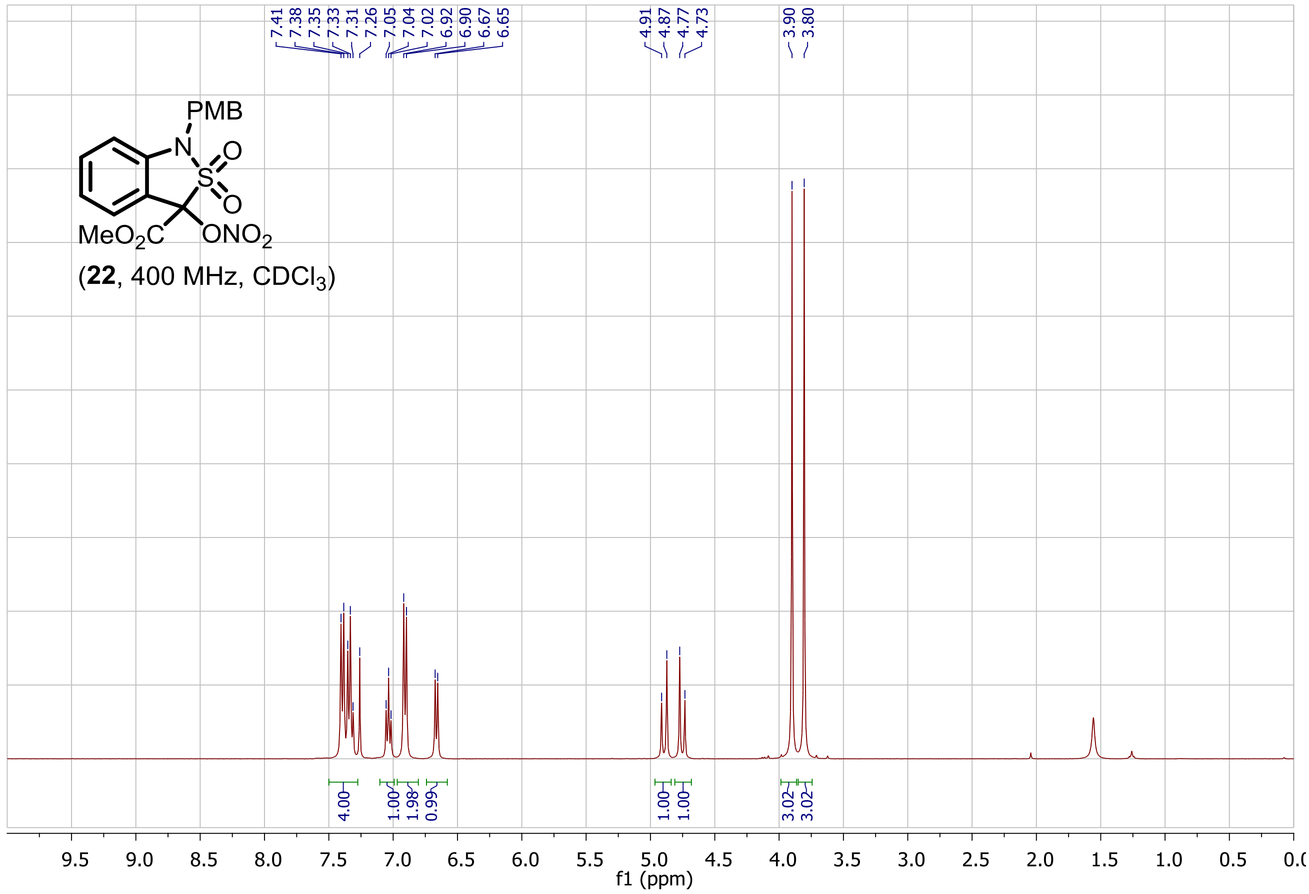
7.41 7.38 7.35 7.33 7.31 7.26 7.05 7.04 7.02 6.92 6.90 6.67 6.65
4.91 4.87 4.77 4.73
3.90 3.80
PMB
N
S
O
O
MeO2C
ONO2
(22, 400 MHz, CDCl3)
4.00
1.00
1.98
0.99
1.00
1.00
3.02
3.02
9.5 9.0 8.5 8.0 7.5 7.0 6.5 6.0 5.5 5.0 4.5 4.0 3.5 3.0 2.5 2.0 1.5 1.0 0.5
f1 (ppm)

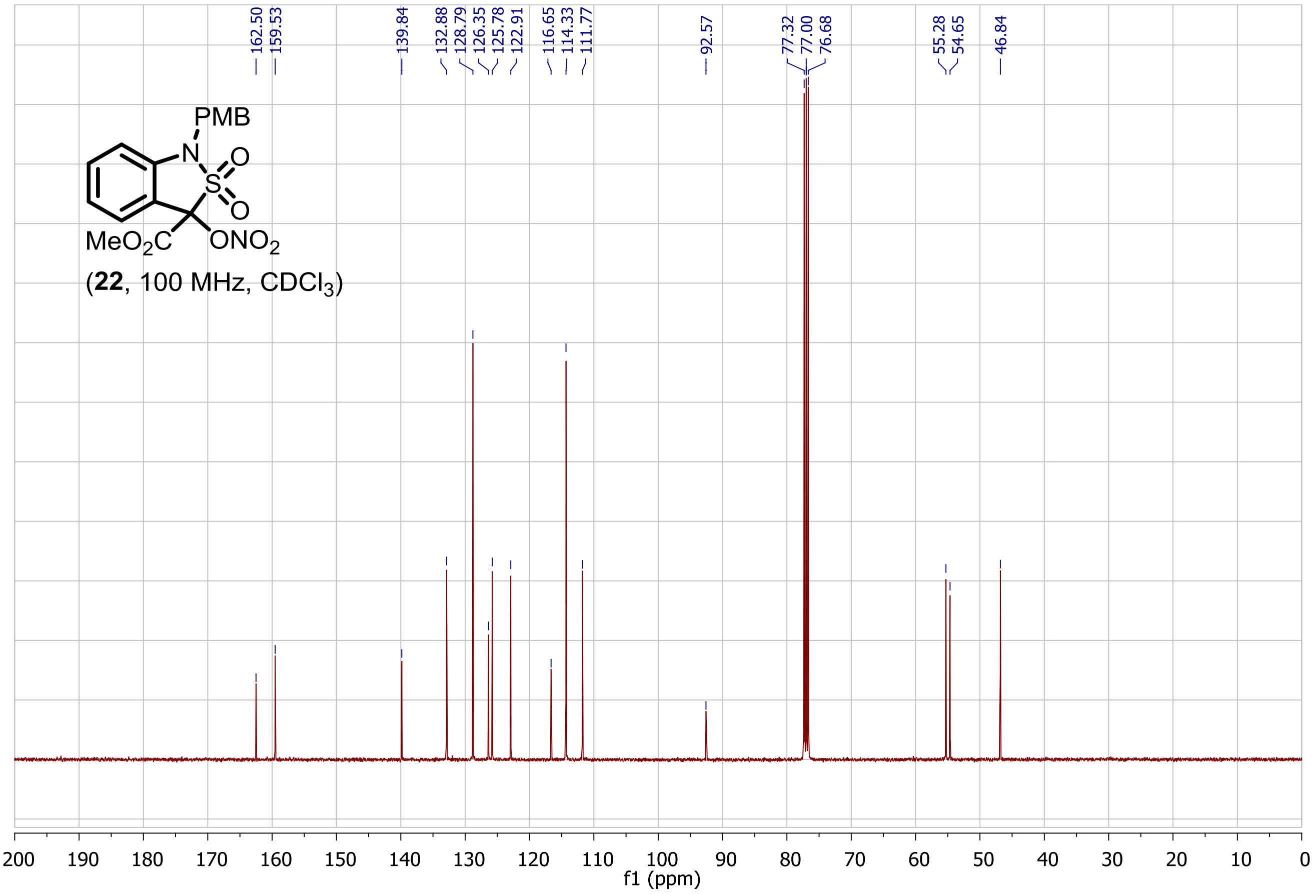
162.50 159.53
139.84
132.88 128.79 126.35 125.78 122.91
116.65 114.33 111.77
92.57
77.32 77.00 76.68
55.28 54.65
46.84
PMB
N
S
O
O
MeO2C
ONO2
(22, 100 MHz, CDCl3)
200 190 180 170 160 150 140 130 120 110 100 90 80 70 60 50 40 30 20 10 0
f1 (ppm)

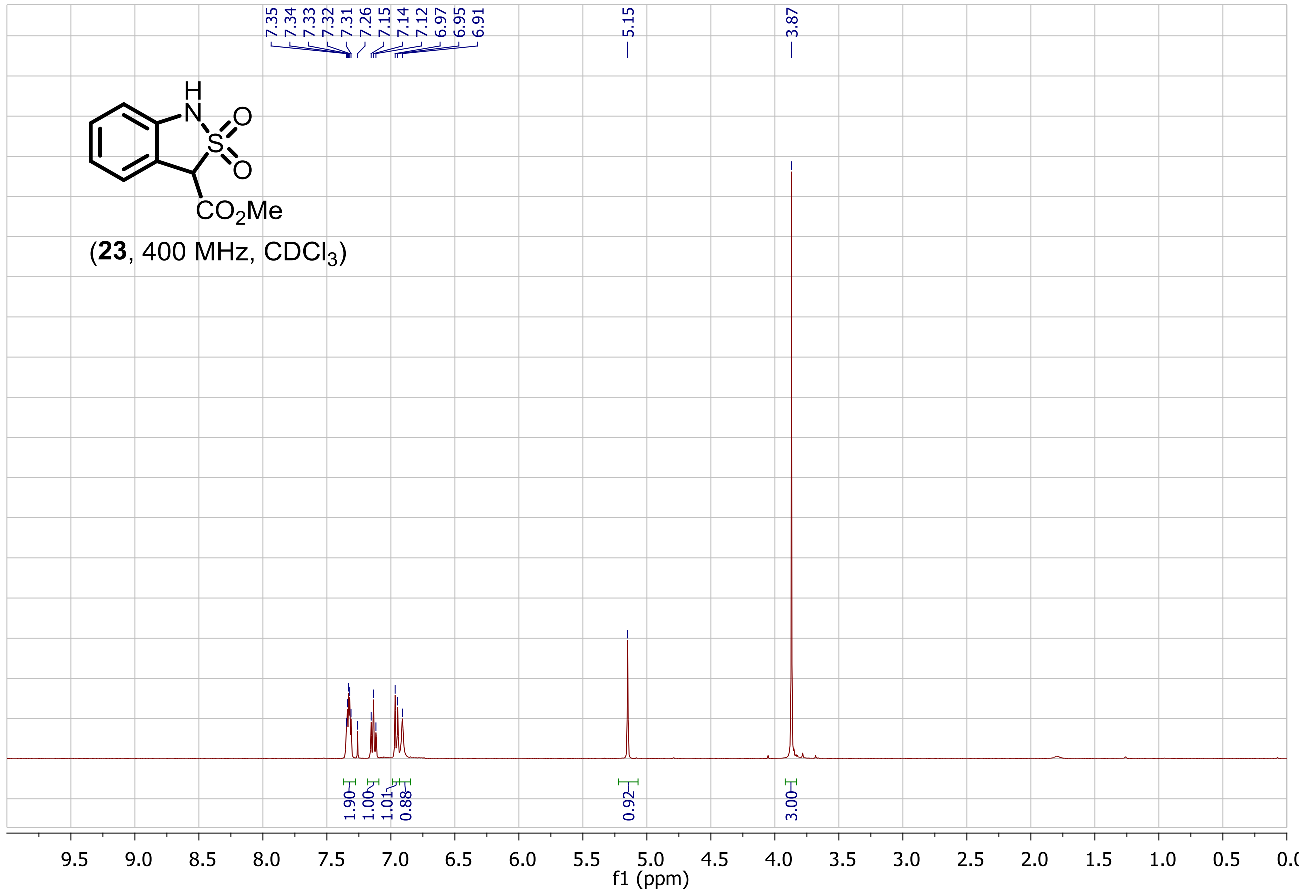
7.35
7.34
7.33
7.32
7.31
7.26
7.15
7.14
7.12
6.97
6.95
6.91
5.15
3.87
(23, 400 MHz, CDCl3)
1.90
1.00
1.01
0.88
0.92
3.00
f1 (ppm)

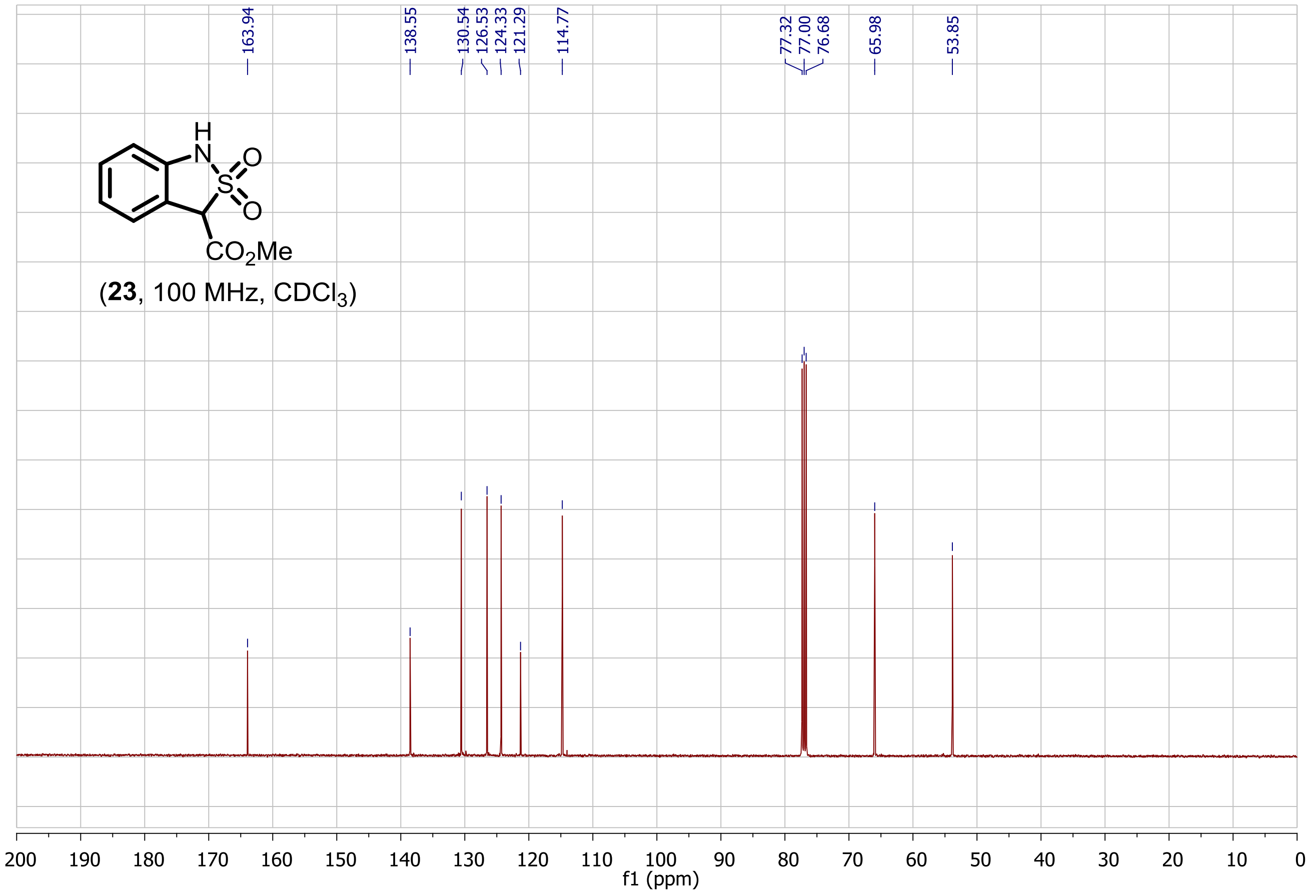
163.94
138.55
130.54
126.53
124.33
121.29
114.77
77.32
77.00
76.68
65.98
53.85
(23, 100 MHz, CDCl3)
f1 (ppm)

4 X-ray crystallographic data

*4.1. Structure analysis of crystal of methyl 5-chloro-1-(4-methoxybenzyl)-1,3-dihydrobenzo[c]isothiazole-3-carboxylate-2,2-dioxide (**10c**)*

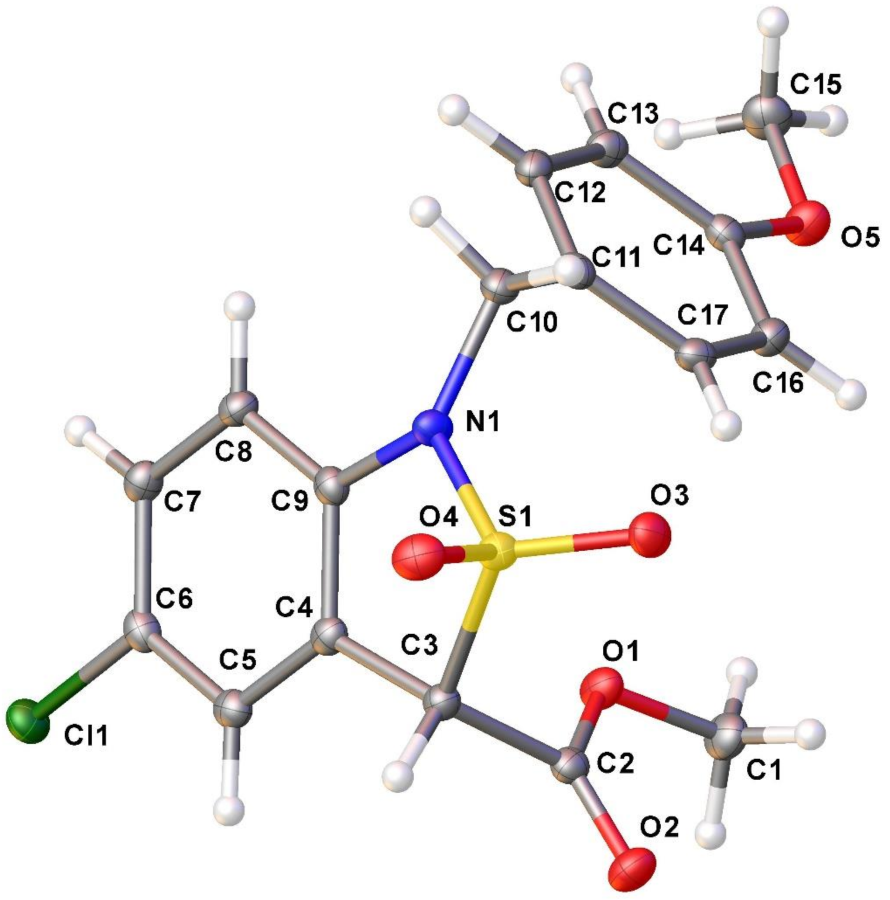

CCDC: **1520988**

Empirical formula	$C_{17}H_{16}ClNO_5S$
Formula weight	381.82
Temperature/K	100.01(10)
Crystal system	triclinic
Space group	P-1
a/Å	9.1882(6)
b/Å	9.7653(6)
c/Å	11.2245(5)
α/°	67.539(5)
β/°	75.412(5)
γ/°	64.377(6)
Volume/Å^3	834.64(10)
Z	2
ρ_{calc}g/cm^3	1.519
μ/mm^{-1}	3.461
F(000)	396.0

Crystal size/mm^3	0.2 × 0.2 × 0.15
Radiation	CuKα (λ = 1.54184)
2Θ range for data collection/°	8.57 to 152.65
Index ranges	-11 ≤ h ≤ 11, -12 ≤ k ≤ 12, -12 ≤ l ≤ 14
Reflections collected	9802
Independent reflections	3483 [R_{int} = 0.0305, R_{sigma} = 0.0319]
Data/restraints/parameters	3483/0/228
Goodness-of-fit on F^2	1.050
Final R indexes [I>=2σ (I)]	R_1 = 0.0324, wR_2 = 0.0815
Final R indexes [all data]	R_1 = 0.0380, wR_2 = 0.0858
Largest diff. peak/hole / e Å^{-3}	0.36/-0.37

*4.2. Structure analysis of crystal of methyl 1-(4-methoxybenzyl)-3-(nitrooxy)-1,3-dihydrobenzo[c]isothiazole-3-carboxylate-2,2-dioxide (**22**)*

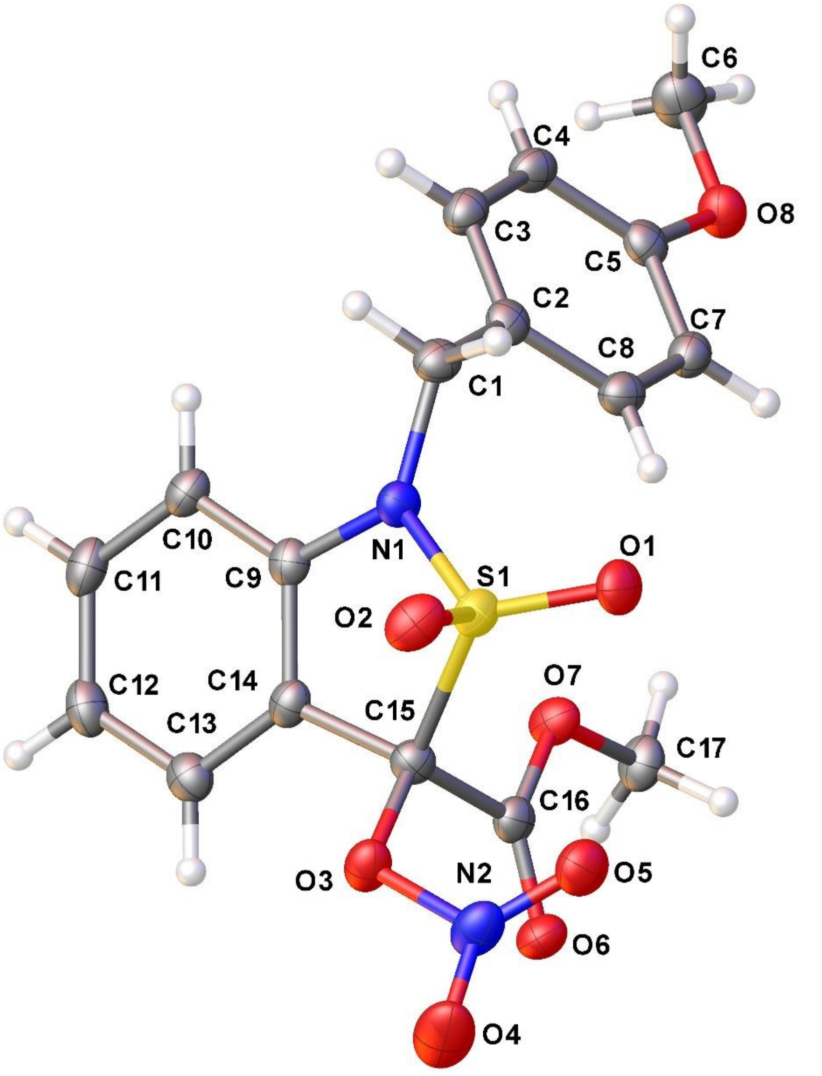

CCDC: **1520989**

Empirical formula	$C_{17}H_{16}N_2O_8S$
Formula weight	408.38
Temperature/K	99.99(10)
Crystal system	triclinic
Space group	P-1
a/Å	9.0077(5)
b/Å	10.7469(5)
c/Å	11.1011(4)
α/°	106.476(4)
β/°	102.298(4)
γ/°	114.630(5)
Volume/Å^3	866.51(8)
Z	2
ρ_{calc}g/cm^3	1.565

μ/mm^{-1}	2.143
F(000)	424.0
Crystal size/mm^3	0.1 × 0.09 × 0.06
Radiation	CuKα (λ = 1.54184)
2Θ range for data collection/°	8.98 to 153.206
Index ranges	$-11 \leq h \leq 11$, $-13 \leq k \leq 13$, $-13 \leq l \leq 13$
Reflections collected	18962
Independent reflections	3615 [R_{int} = 0.0623, R_{sigma} = 0.0310]
Data/restraints/parameters	3615/0/255
Goodness-of-fit on F^2	1.047
Final R indexes [I>=2σ (I)]	R_1 = 0.0407, wR_2 = 0.1092
Final R indexes [all data]	R_1 = 0.0451, wR_2 = 0.1135
Largest diff. peak/hole / e $Å^{-3}$	0.41/-0.48
